# The Hippo Pathway Targets Rae1 to Regulate Mitosis and Organ Size and to Feed Back to Regulate Upstream Components Merlin, Hippo, and Warts

**DOI:** 10.1371/journal.pgen.1006198

**Published:** 2016-08-05

**Authors:** Maryam Jahanshahi, Kuangfu Hsiao, Andreas Jenny, Cathie M. Pfleger

**Affiliations:** 1 Department of Oncological Sciences, The Icahn School of Medicine at Mount Sinai, New York, New York, United States of America; 2 The Graduate School of Biomedical Sciences, The Icahn School of Medicine at Mount Sinai, New York, New York, United States of America; 3 Department of Neuroscience, The Icahn School of Medicine at Mount Sinai, New York, New York, United States of America; 4 Department of Developmental and Molecular Biology and Department of Genetics, Albert Einstein College of Medicine, Bronx, New York, New York, United States of America; HHMI/Rutgers University, UNITED STATES

## Abstract

Hippo signaling acts as a master regulatory pathway controlling growth, proliferation, and apoptosis and also ensures that variations in proliferation do not alter organ size. How the pathway coordinates restricting proliferation with organ size control remains a major unanswered question. Here we identify Rae1 as a highly-conserved target of the Hippo Pathway integrating proliferation and organ size. Genetic and biochemical studies in *Drosophila* cells and tissues and in mammalian cells indicate that Hippo signaling promotes Rae1 degradation downstream of Warts/Lats. In proliferating cells, Rae1 loss restricts cyclin B levels and organ size while Rae1 over-expression increases cyclin B levels and organ size, similar to Hippo Pathway over-activation or loss-of-function, respectively. Importantly, Rae1 regulation by the Hippo Pathway is crucial for its regulation of cyclin B and organ size; reducing Rae1 blocks cyclin B accumulation and suppresses overgrowth caused by Hippo Pathway loss. Surprisingly, in addition to suppressing overgrowth, reducing Rae1 also compromises survival of epithelial tissue overgrowing due to loss of Hippo signaling leading to a tissue “synthetic lethality” phenotype. Excitingly, Rae1 plays a highly conserved role to reduce the levels and activity of the Yki/YAP oncogene. Rae1 increases activation of the core kinases Hippo and Warts and plays a post-transcriptional role to increase the protein levels of the Merlin, Hippo, and Warts components of the pathway; therefore, in addition to Rae1 coordinating organ size regulation with proliferative control, we propose that Rae1 also acts in a feedback circuit to regulate pathway homeostasis.

## Author Summary

Exquisite control of organ size is critical during animal development and its loss results in pathological conditions. The Hippo Tumor Suppressor Pathway coordinates regulation of proliferation, growth, apoptosis, and autophagy to determine and maintain precise control of organ size. However, the genes responsible for Hippo-mediated regulation of mitosis or coordination of proliferation within organ size control have evaded characterization. Here, we describe Rae1, an essential WD-repeat containing protein, as a new organ size regulator. By genetic analysis, we show that Rae1 acts downstream of the Hippo Pathway to regulate mitotic cyclins and organ size. In contexts where organ size control is lost by compromised Hippo signaling, we show that there is a requirement for Rae1 that is distinct from the requriement for Yki: reducing Yki levels causes suppression of overgrowth, while reducing Rae1 levels dramatically compromises the survival of Hippo-deficient tissue. Lastly, our studies of Rae1 uncovered a potential post-transcriptional feedback loop that reinforces Yorkie-mediated transcriptional feedback for the Hippo Pathway.

## Introduction

The Hippo Pathway (also called the Salvador-Warts-Hippo Pathway) plays a well-appreciated and strongly conserved developmental role in establishing and maintaining organ size. Aberrations in signaling pathways can increase rates of cellular growth or proliferation, but once appropriate organ size is reached, what is coming to be called an “organ size checkpoint” blocks further growth and proliferation; organs do not overgrow unless these aberrations also bypass the “organ size checkpoint” [1]. The precise nature of the signal that restricts cell division in response to organ size checkpoint activation remains unknown. Given that loss of Hippo signaling (1) results in both tissue and organ overgrowth in *Drosophila* and vertebrates and (2) is implicated in a range of cancers including colorectal cancer, liver cancer, melanoma, lung cancer, leukemia, and ovarian cancer [2–11; for review see 12–19], elucidating this link between proliferation control and organ size control within the Hippo Pathway has important implications for development and disease.

The Hippo Pathway consists of a core cassette: Hippo (Hpo), Warts (Wts), Salvador (Sav) and Mats [19–25]. Hpo (homologous to mammalian Mst1 and Mst2), the upstream serine/threonine kinase in the cassette, phosphorylates the scaffold protein Sav (hWW45 or SAV1 in mammals), the downstream kinase Wts (Lats1 and Lats2 in mammals), and Wts co-activator Mats (Mob1 in mammals). Activated Wts then phosphorylates transcriptional co-activator Yorkie (Yki) (YAP and TAZ in humans) [26] promoting its cytoplasmic retention where it cannot regulate transcription of cell death, cell division, and cell growth regulators such as *DIAP1*, *cyclin E*, and others [27–28]. The pathway is also subject to feedback through Yki/YAP-dependent transcription of upstream regulators such as *Merlin* (*Mer*) and *expanded* (*ex*) in *Drosophila* tissues [26, 29], and Lats2 and NF2 in mammalian cultured cells [30]. The core components and Yki/YAP thus play a crucial role in the Hippo Pathway’s global regulation of organ homeostasis.

Early characterization of Hippo Pathway mutants uncovered a role for the pathway in regulating mitotic progression, consistent with a role for yeast homologs in the mitotic exit/septation initiation networks. Hpo depletion in *Drosophila* S2 cells causes mitotic and central spindle defects [31]. Similarly, *mats* mutant embryos show chromosome segregation defects [32] and Mats over-expression has been shown to regulate cytokinesis [33], suggesting a role for *mats* in mitotic exit in *Drosophila*. Importantly, *mats* mutant imaginal discs show increased cyclin A (cycA) and cyclin B (cycB) levels [32] and *wts* mutant discs show increased cycA levels [34]. The restriction of cycA is functionally important in restricting organ size downstream of Wts [32]. Mutations in the mammalian tumor suppressor components of the pathway have also been extensively characterized for their regulation of centrosomal dynamics [35–36], mitotic exit/cytokinesis [37–39], and tetraploidy checkpoint [40]. Together, the data suggest that Hippo Pathway components control appropriate mitotic cyclin levels in *Drosophila* cells and also have more specific roles regulating the mitotic spindle and genome integrity. YAP and TAZ have not been characterized as regulators of mitotic exit and cytokinesis, so it remains unclear how the pathway regulates cyclin levels and mitotic progression. Understanding this process will shed light on the complicated mechanism by which Hippo signaling coordinates proliferation and organ homeostasis.

Here we identify Rae1 as an important and highly conserved regulator of proliferation and organ size. Rae1 is a WD repeat protein first identified for a role in RNA export from the nucleus in yeast [41] and now with reported roles in spindle assembly [42], regulation of the Anaphase Promoting Complex/Cyclosome (APCC) [43–45], regulation of the E3 Highwire [46], and spermatogenesis [47].

In this work, we present genetic and biochemical studies showing that Hippo signaling promotes Rae1 degradation downstream of Wts/Lats, and parallel to the pathway’s regulation of Yki/YAP. Importantly, Rae1 is epistatic to Wts in its regulation of cycB, and Hippo signaling regulation of Rae1 is functionally relevant to its organ size functions. Instead of an “on/off” switch for organ growth, our data implicates Rae1 as a molecular rheostat for organ size control. Complementing Yki/YAP’s role to transcriptionally regulate upstream Hippo Pathway components, we also define a role for Rae1 to regulate the levels and activity of Hippo Pathway components post-transcriptionally in a proposed feedback circuit to ensure Hippo Pathway homeostasis.

## Results

### The Hippo Pathway promotes Rae1 degradation downstream of Wts/Lats and parallel to Yki/YAP *in vitro* and *in vivo*

**Fig 1:**
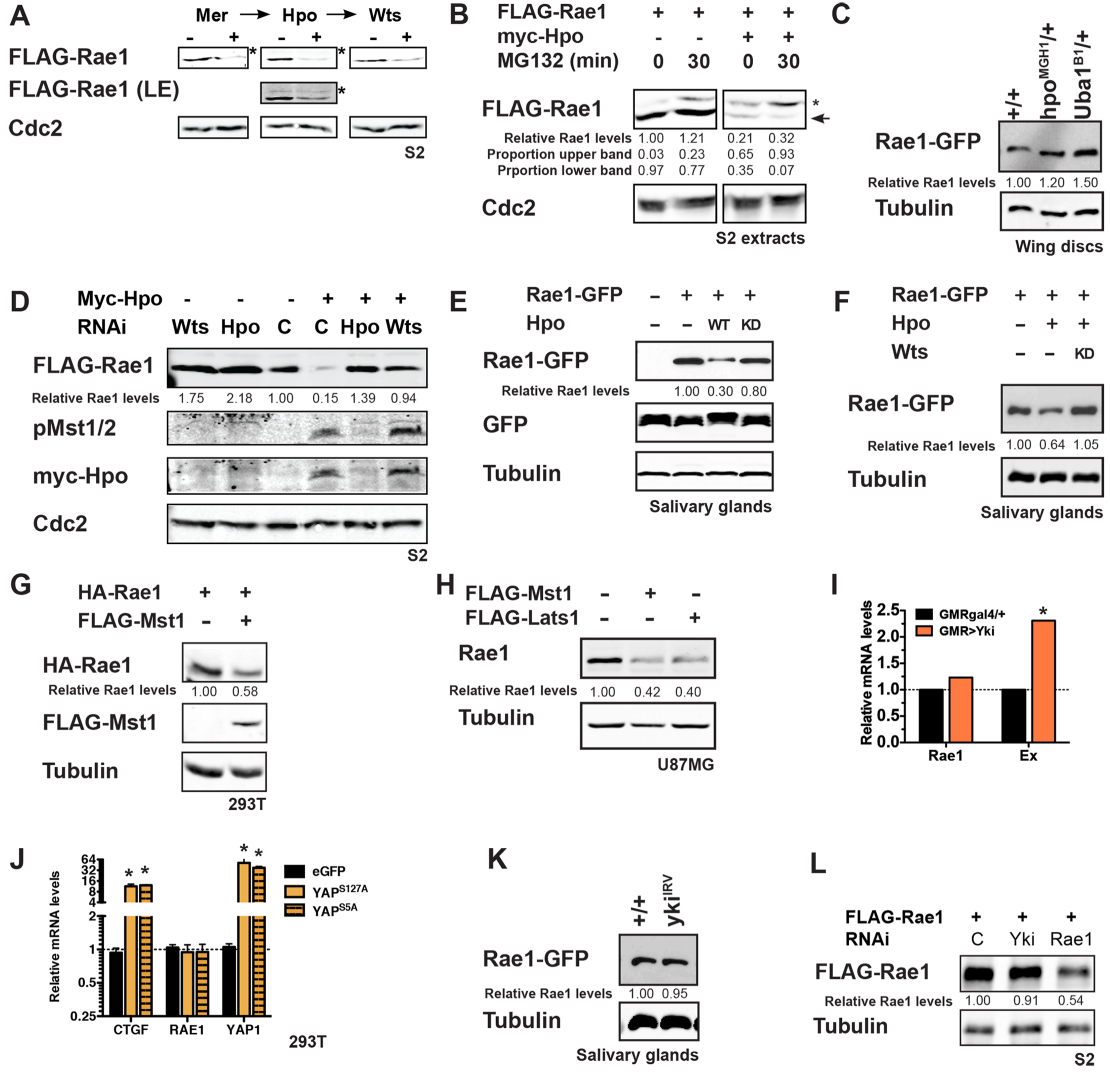
Hippo signaling plays a highly conserved role to restrict Rae1 protein levels independently of Yki/YAP. (A) Co-transfecting S2 cells with *Mer*, *hpo*, or *wts* causes loss of Rae1 protein levels compared to control-transfected cells. *faint, shifted band in cell lysates. Longer exposure of the Hpo panel (LE) makes the slower migrating band more visible. (B) An extract of S2 cells transfected with *Rae1* (left panels) or *Rae1* and *hpo* (right panels) created in the presence of phosphatase inhibitors and cycloheximide shows two bands. In extracts from the *hpo*-co-transfected cells, there was less Rae1 overall, and the upper band (*) predominated when incubated with MG132 (right panel, 30 min). (C) Impairing Hippo signaling by heterozygosity at the *hpo* locus (*hpo*^*MGH1*^*/+*, lane 2) or impairing proteasomal activity by heterozygosity at the E1 Ubiquitin activating enzyme locus (*Uba1*^*B1*^*/+*, lane 3) increases Rae1-GFP protein levels compared to control (*+/+*, lane 1) in *Drosophila* wing discs. (D) Rae1 is more stable (>70% by quantitation) in cells after RNAi knockdown of *hpo* or *wts* (two left-most lanes) compared to control RNAi (third lane). RNAi to *wts* stabilizes Rae1 in the presence of co-transfected *hpo* (right-most lane) compared to cells treated with control RNAi (fourth lane). Anti-Myc (tag on Hpo) and anti phospho-MST (pMST, a phospho-specific antibody to mammalian Mst1/Mst2 which cross-reacts with *Drosophila* Hpo to label the activated form [51]) blots confirm expression and activation of Hpo with RNAi to *wts* (right-most lane) indicating that Rae1 destabilization required Wts activity. Cdc2 (PSTAIR) blot acts as a loading control. (E) Over-expressing a wild-type (lane 3) but not a kinase-dead (lane 4) Hpo transgene in the context of Rae1-GFP over-expression in salivary glands shows a reduction in Rae1-GFP protein compared to controls (lane 2). Hpo transgene expression did not affect GFP protein levels or salivary gland organ size. (F) Over-expressing kinase-dead Wts (lane 3) restores Hpo-mediated reduction in Rae1-GFP protein levels (lane 2 compared to control in lane 1) in salivary glands. (G) HEK293T cells expressing human HA-Rae1 were co-transfected with Mst1, showing a loss of Rae1 levels. (H) Over-expression of Mst1 or Lats1 in U87MG cells shows loss of endogenous Rae1 protein levels compared to control-transfected cells. (I) Over-expressing *yki* in differentiating eye cells (*GMR Yki*) increases the relative mRNA levels (normalized to GAPDH) of Yki target *ex* (orange bars) [26], but not the relative mRNA levels of *Rae1* (black bars) compared to a *GMRgal4* control. (J) Over-expression of constitutively active Yap constructs (YAP^S127A^, orange bars, or YAP^S5A^, orange, striped bars) in HEK-293T cells increases the relative mRNA levels (normalized to GAPDH) of YAP target *CTGF* [52] but not the relative mRNA levels of *Rae1*. (K) *Yki* knockdown via RNAi causes no change in Rae1-GFP protein levels (lane 2) compared to control salivary glands (lane 1). (L) Co-transfecting S2 cells with *yki* RNAi causes no change in Rae1 protein levels compared to control-transfected cells (over three independent experiments), while Rae1 RNAi causes a statistically significant decrease in Rae1 protein levels. Relative Rae1 levels (normalized by Cdc2 in A, B, D, Tubulin in C, F, G, H, K, L and GFP in E) are indicated for blots A-H and K-L. In B, this is followed by a breakdown of the relative proportion of the slower (second line) and faster (third line) migrating species in each lane (normalized to total Rae1). * indicates statistically significant difference p<0.05.

We identified Rae1 in a *Drosophila in vitro* expression cloning (DIVEC) screen [48–50] for *in vitro* translated (IVT) proteins whose stability or migration on a gel was affected by supplementing IVT reticulocyte lysates with recombinant Mst1 and Mst2 proteins (S1A–S1C Fig, [50], experimental detail is provided in the Materials and Methods section). To evaluate if Rae1 played a role in Hippo signaling, we first characterized Hippo signaling regulation of Rae1 stability *in vitro* in tissue culture cells and *in vivo* in *Drosophila*. In S2 cells, co-transfected Hippo Pathway tumor suppressor components *Mer*, *hpo*, or *wts* each promoted a reduction in Rae1 levels (Fig 1A). Longer exposures showed a slower migrating band (Fig 1A) that decreased when incubated with phosphatase (S2A Fig), and experiments in S2 extracts which preserve proteasomal activity (see Materials and Methods for extract protocol) showed accumulation of this band in the presence of MG132 and phosphatase inhibitors (Fig 1B), suggesting that Hippo signaling promotes a phosphorylation-dependent mobility shift and Rae1 degradation by the proteasome. Consistent with this, reducing the gene dosage of *hpo* or *wts in vivo* in *Drosophila* or impairing proteasome function by heterozygosity in E1 (*Uba1*, the most upstream enzyme in the Ubiquitin Pathway), increased Rae1 protein levels as monitored by the levels of a GFP-tagged Rae1 transgene, *Rae1*^*GFP*^ [46] (Fig 1C and S2B–S2D Fig). RNAi knockdown of *hpo* or *wts* stabilized co-transfected Rae1, and RNAi to *wts* prevented Hpo-induced degradation of Rae1 in S2 cells (Fig 1D and S2E Fig). Consistent with this, Rae1 protein levels were negatively regulated by Hippo and Warts kinase activity *in vivo* in *Drosophila* imaginal discs and salivary glands (Fig 1E and 1F and S2F Fig). The ability of co-transfected *wts* to destabilize Rae1 (Fig 1A) and of *wts* inhibition (through RNAi *in* vitro, Fig 1D and S2E Fig or over-expression of a kinase-dead transgene *in vivo*, Fig 1F) to stabilize Rae1 in the presence of over-expressed Hpo indicates that Wts activity is required downstream of Hpo for regulating Rae1 protein levels in *Drosophila* cells and in tissues.

Importantly, regulation of Rae1 by Hippo signaling is highly conserved. Activating Hippo signaling by over-expressing Mst1 and/or Lats1 promoted loss of co-transfected Rae1 in immortalized HEK-293T cells (Fig 1G) and loss of endogenous Rae1 in tumorigenic U87-MG (Fig 1H) or HeLa cells (S2G Fig). Rae1 loss was dose-responsive to Hippo signaling (S2H Fig) and not due to cell death (S2I Fig).

To address if Rae1 is a direct target of the Warts/Lats kinase, we tested if immunoprecipitated Rae1 was recognized by a phospho-RXXS antibody (the consensus Lats1 site [53]) (S3A Fig). The percentage of immunoprecipitated Rae1 phosphorylated at the RXXS site increased in a dose-responsive manner to increased pathway activation (S3A Fig). Like many WD repeat proteins, recombinant *Drosophila* or human Rae1 purified from bacteria was insoluble and refolding attempts resulted in largely aggregated protein unsuitable for direct kinase assays. Therefore, we utilized small Rae1 peptides containing the putative Rae1 phosphorylation site (S3B Fig). Despite recognition of endogenous Rae1 by the phospho-RXXS antibody, purified system kinase assays using recombinant Lats2 and a Rae1 peptide of this site failed to show phosphorylation even when showing robust phosphorylation of a control YAP peptide (S3B Fig). Kinase assays with full-length baculovirus-produced Rae1 (a gift from Y. Ren and the Blobel lab, [54]) showed insignificant phosphorylation by Lats2 compared to a positive control (S3C Fig). Recognition by phosho-RXXS antibodies but failure of Lats2 to recognize Rae1 peptides or baculovirus-expressed Rae1 may reflect that Warts/Lats kinase directly phosphorylates full length Rae1 when in specific complexes with other proteins or requires a priming phosphorylation. Alternatively, the Warts-dependent Rae1 targeting observed (Fig 1 and S2 Fig) may occur further downstream.

To exclude that changes in Rae1 are in part due to a transcriptional effect of Yki/YAP, we conducted qRT-PCR of adult heads expressing a *yki* transgene (GMR>Yki, Fig 1I) and of mammalian cells over-expressing activated YAP (YAP^S127A^ and YAP^S^^5^^A^, Fig 1J). Both cases showed increased levels of well-characterized transcriptional targets (*expanded*, a Yki target in flies [26] and *CTGF*, a transcriptional target of YAP in mammalian systems [52]), confirming increased Yki/YAP transcriptional activity, but did not show increased *Rae1* transcripts.

Our *in vitro* extract experiments in the presence of cycloheximide (Fig 1B) showed accumulation of a slower-migrating form of Rae1 from the initial time point to the 30 minute time point; this does not rule out that Hippo signaling affected Rae1 via transcriptional means in cells before extract creation but does suggest a means by which Hippo signaling regulates Rae1 post-translationally. If this occurred by a non-transcriptional role of Yki to regulate Rae1 protein levels, modulating the levels of Yki should modulate Rae1 levels. Reducing *yki* levels by RNAi had no substantial effect on Rae1 protein levels or localization in S2 cells or in larval tissues (Fig 1K and 1L and S3D–S3G Fig). Consistent with this, over-expressing activated YAP in 293T cells did not increase Rae1 protein levels (S3H Fig). These findings suggest that the Hippo Pathway does not downregulate Rae1 levels through Yki/YAP via transcriptional or post-translational mechanisms.

### Rae1 loss decreases organ and organism size

To investigate if the negative regulation of Rae1 by the Hippo Pathway is functionally relevant in restricting proliferation, organ size, and promoting apoptosis, we first characterized the phenotypes of reducing or over-expressing Rae1 *in vivo* in the fly. We used previously characterized *Rae1* deletion allele *Rae1*^*ex28*^ [46] and four inducible RNAi lines corresponding to three independent inverted repeat alleles: *P{GD14705}v29303* from the VDRC referred to here as *Rae1*^*IR*V^; *9862R-2* and *9862R-3* from the NIG collection, referred to here as *Rae1*^*IRN2*^ and *Rae1*^*IRN3*^, and *P{TRIP*.*HMS00670}* from the Transgenic RNAi Project referred to here as *Rae1*^*IRT*^. The inverted repeat in *Rae1*^*IRN2*^ and *Rae1*^*IRN3*^ is partially overlapping with *Rae1*^*IR*V^; *Rae1*^*IR*T^ is entirely non-overlapping with *Rae1*^*IR*V^, *Rae1*^*IRN2*^, and *Rae1*^*IRN3*^. To increase *Rae1* gene dosage, we created inducible transgenic alleles *Rae1*^*02*^ and *Rae1*^*03*^, and used previously characterized *Rae1* transgenic allele *Rae1*^*GFP*^ [46] (relative mRNA levels for a subset of these is shown in S4A Fig).

**Fig 2:**
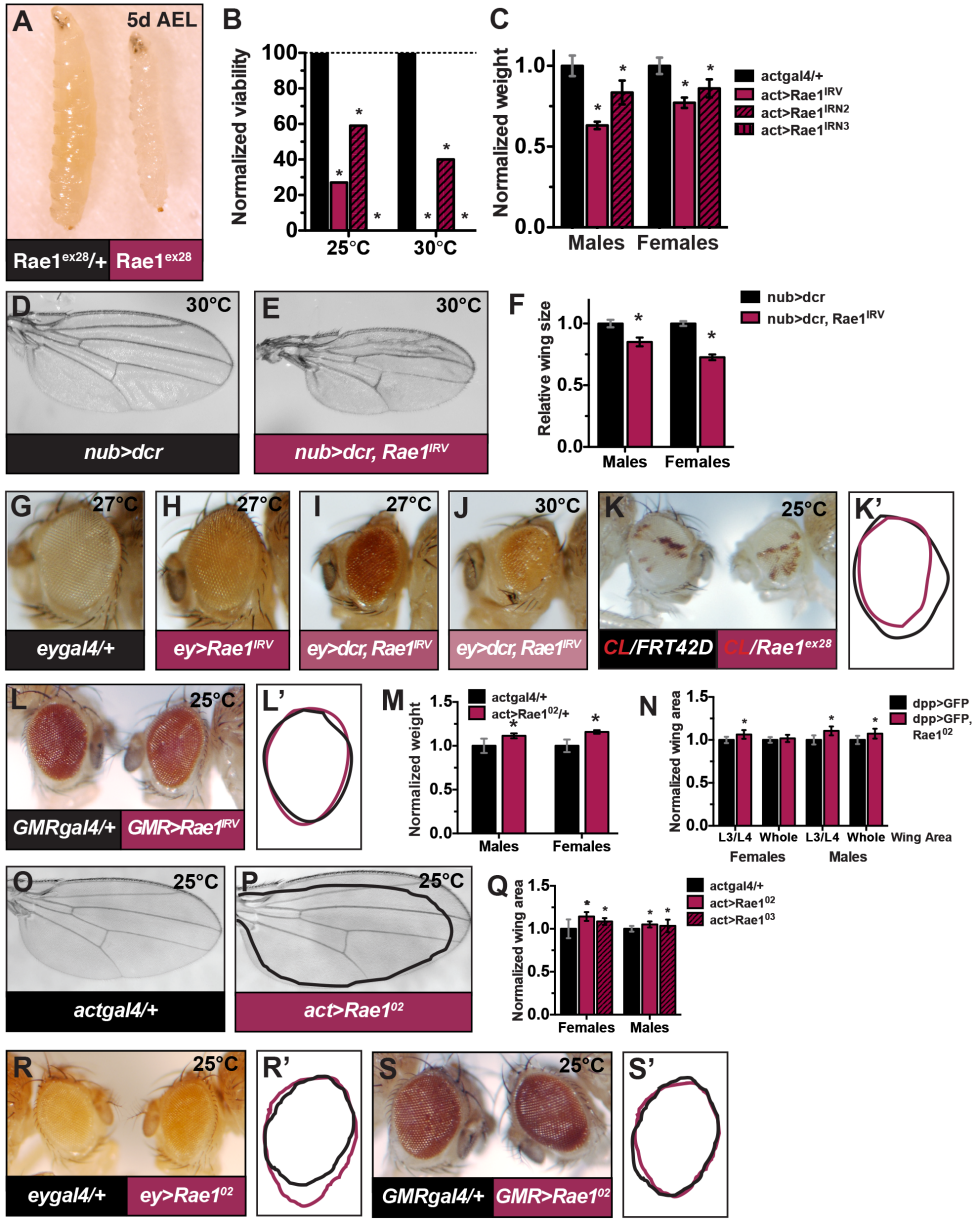
Rae1 regulates organ and organism size in *Drosophila*. (A) *Rae1*^*ex28*^ null third instar larvae (right) are smaller than heterozygous siblings (left). (B-C) Constitutive *Rae1* RNAi reduces adult viability (B) and weight (C) of surviving adults. In (B), N = 229, 236, 131, 263 (for bars in order) for 25°C and N = 107, 52, 85, 212 for 30°C. In C, N = 73, 22, 52 (males) and N = 85, 43, 56 (females). (D) Control *nub>dcr* wing. (E) Decreasing *Rae1* by RNAi (*nub>dcr*, *Rae1*^*IRV*^) reduces wing size. (F) Quantification of the wings in D-E. N = 15, 18, 13, 17. (G) Control *eygal4/+* eye. (H-J) Decreasing *Rae1* by RNAi decreases eye size and causes eye roughness (*ey>Rae1*^*IRV*^, H). Eye size is further decreased by co-expression of *dcr* (*ey>dcr*, *Rae1*^*IRV*^, I) and development at a higher temperature (*ey>dcr*, *Rae1*^*IRV*^, 30°C, J). Quantification for these eyes is shown in S4L Fig. (K-K’) Eyes containing primarily *Rae1*^*ex28*^ tissue (*yweyFLP; FRT42D Rae1*^*ex28*^*/FRT42D l(2) pW+*, right eye, traced in pink in K’) were smaller and rough compared to control eyes (*yweyFLP; FRT42D/FRT42D l(2) pW+*, left eye, traced in black in K’; quantification shown in S4M Fig). Red tissue remaining is heterozygous (“un-flipped”) tissue. (L-L’) Decreasing *Rae1* by RNAi (right eye, traced in pink in M’) in differentiating eye tissue causes no obvious visible phenotypes compared to controls (left eye, traced in black in L’; quantification shown in S4Q Fig). (M) Constitutive Rae1 over-expression increases overall organism size in terms of weight (shown) and body length (S4R Fig). N = 103, 64, 122, 91. (N) *Rae1* over-expression in a stripe in the developing wing using *dppgal4* and *Rae1*^*02*^ (*dpp>dcr*, *GFP*, *Rae1*^*02*^) increased the area of the wing between the L3 and L4 wing veins and also increased overall wing area in males. N = 13, 15 (females), N = 14, 14 males. (O-Q) Constitutive Rae1 over-expression also increased eye size (S4S and S4S’ Fig) as well as wing size (P) compared to control (O, black tracing in P). (Q) Quantification of wings in O-P. N = 17, 15, 13 (females), N = 14, 12, 17 (males). (R-R’) Over-expressing Rae1 in the early eye (*ey>Rae1*^*02*^, right, pink tracing in R’) increases eye size compared to control (*eygal4/+*, left eye, black tracing in R’). (S-S’) Rae1 over-expression in the differentiating cells of the eye (right eye in S, pink tracing in S’) shows no obvious visible phenotypes compared to control eyes (left eye in S, black tracing in S’) Eyes quantified in S4Q Fig. * indicates statistically significant change from controls, p<0.05.

Larvae homozygous for deletion of *Rae1* or undergoing strong, constitutive *Rae1* RNAi died as small wandering third-instars (Fig 2A and 2B, [46]). Their imaginal discs were smaller than control heterozygous animals. Reducing *Rae1* levels by low level RNAi resulted in viable adults of reduced weight and size (Fig 2C). *Rae1* RNAi in the developing wing disc using *nubgal4* resulted in adult flies with smaller wings (Fig 2D–2F). Similar phenotypes were observed using different wing drivers and additional RNAi lines (S4B–S4J Fig) or by RNAi in a stripe in the wing (S4K Fig). *Rae1* RNAi in the proliferating cells of the developing eye disc using *eygal4*, resulted in adult flies with smaller eyes (Fig 2G–2J, quantified in S2L Fig). Eyes containing primarily homozygous *Rae1*^*ex28*^ mutant tissue were also small (Fig 2K and 2K’, quantified in S4M Fig). Furthermore, organ size reduction was seen with non-overlapping RNAi lines, and Rae1 over-expression rescued RNAi phenotypes in the eye and wing (S4N–S4P Fig) indicating that the reduced organ size phenotypes resulted specifically from *Rae1* reduction and not off-target effects. In contrast, *Rae1* RNAi in differentiating eye cells (*GMR>Rae1*^*IRV*^) resulted in no obvious phenotype (Fig 1L and 1L’ and S4Q Fig).

### Rae1 over-expression increases organ and organism size

The Rae1 loss-of-function phenotypes could result from an essential cell function or from a normal role of Rae1 to promote organ size. If Rae1 positively regulates organ size, its over-expression should increase organ size. Constitutive Rae1 over-expression increased overall organism size in terms of weight and body length (Fig 2M and S4R Fig), increased wing size (Fig 2O and 2P, quantified in 2Q), and increased eye size (S4S-S4S’ Fig). Over-expressing Rae1 in the whole wing or specific compartments also increased wing size (Fig 2N). Over-expressing Rae1 in the proliferating cells (Fig 2R and 2R’) (but not the differentiating cells only, Fig 2S and 2S’, quantified in S4Q Fig) of the eye increased eye size. Despite larger overall size, Rae1 over-expressing eyes appeared normally-patterned and showed no change in ELAV (a neuronal marker) expression in third instar eye discs (S4T Fig). This is in stark contrast to Yki over-expression in the early eye. Wild-type and constitutively active Yki expression in proliferating cells of the eye with *eygal4* or constitutively with *Actgal4* resulted in loss of eye structures (S4U-S4V’ Fig) reminiscent of *ex* loss [55–56] and as seen with Yki expression limited to the dorsal-ventral margins with *bigal4* [57]. As with loss of *ex*, the block in differentiation from Yki over-expression with eygal4 in our study or *bigal4* [57] was suppressed by loss of *wingless* (*wg*) so likely resulted from effects of increased *wg* blocking progression of the Morphogenetic Furrow (MF). All together, the organ size phenotypes of Rae1 loss-of-function or over-expression are consistent with a role for Rae1 to promote organ size and are consistent with Rae1 inhibition by the Hippo Pathway (Fig 1 and S2 Fig) to restrict organ size.

### Rae1 modulation does not result in increased apoptosis or differentiation effects

Effects on organ size can result from changes in cell size. Forward scatter of cells dissociated from dissected mosaic wing discs containing clones of *Rae1* RNAi using *Rae1*^*IRV*^ and *Rae1*^*IRN2*^ showed no difference in size of cells undergoing RNAi to *Rae1* (GFP-positive cells) compared to control cells (GFP-negative cells) (S5A Fig). Similarly, forward scatter showed no difference in size for Rae1 over-expressing cells (GFP-positive cells) compared to control cells (GFP-negative cells) (S5B Fig). This suggests that the smaller organ size of Rae1 RNAi and the larger organ size of Rae1 over-expression did not result from changes in cell size.

Smaller organs could also result from increased cell death or from differentiation into other structures. We saw no obvious increase in anti-activated caspase 3 staining or in TUNEL assays upon *Rae1* RNAi (S5C and S5D Fig for TUNEL). Moreover, co-expressing caspase inhibitor p35 did not suppress the eye size phenotype of *Rae1* RNAi (S5E Fig). Consistent with previous studies that *Rae1* RNAi in S2 cells did not promote apoptosis [58], these findings suggest that decreased organ size did not result from increased apoptosis. We observed no effects on ELAV staining upon *Rae1* RNAi in actively dividing cells in the early eye (using *eygal4)* (S5F Fig) suggesting that reducing Rae1 does not cause premature differentiation to reduce organ size.

### Rae1 regulates proliferation

Smaller organs could result from decreased proliferation. In the *Drosophila* larval eye, a wave of differentiation, the MF, passes from posterior to anterior. A subset of cells undergo an additional round of coordinated division called the Second Mitotic Wave (SMW) which appears as a synchronized stripe of dividing cells just posterior to the MF. Cells posterior to the SMW in the eye disc are not dividing at this stage and so do not normally stain for pHH3 and do not undergo BrdU incorporation. When cells in this region are induced to undergo ectopic division (for example, due to over-expression of an oncogene), individual cells will incorporate BrdU or stain for cell cycle markers cycA, cycB, or pHH3 (appropriate for their cell cycle phase). If cells enter S-phase but are endoreplicating or stall before mitosis (so are not actively cycling), they do not stain for pHH3. When cells that have entered the cell cycle arrest in mitosis, cells in the tissue do not incorporate BrdU but stain for pHH3. Effects of Rae1 on the cell cycle can thus be assessed with cell cycle markers in the third instar larval eye.

**Fig 3:**
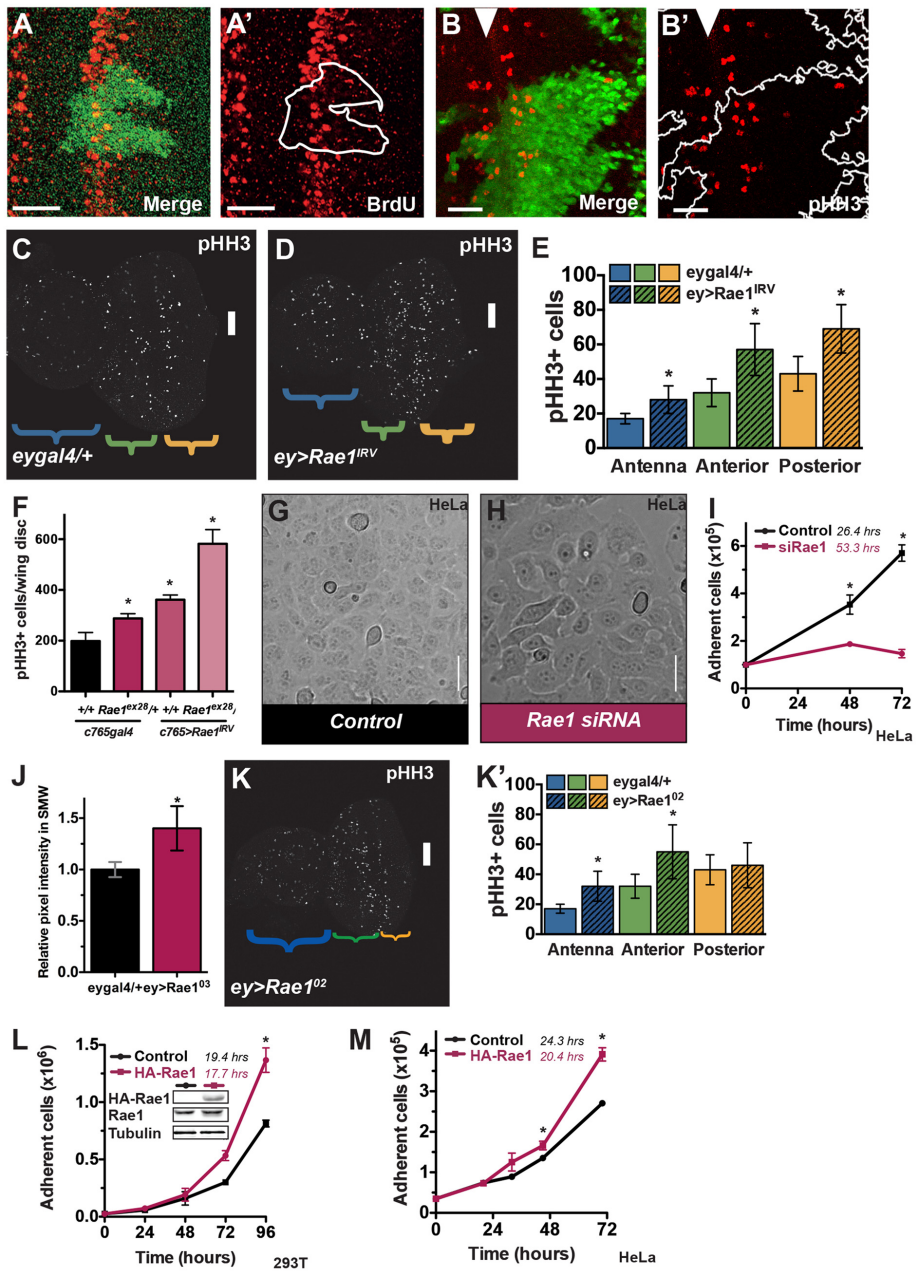
Rae1 regulates proliferation in *Drosophila* and in mammalian cells. (A-A’) Clones undergoing constitutive *Rae1* RNAi (green in A, tracing in A’) show reduced BrdU incorporation (red) compared to adjacent tissue. (B-B’) There was no obvious decrease in pHH3 positive cells (red) in *Rae1* RNAi clones (green in B, tracing in B’). Arrowhead indicates the position of the MF in this and subsequent figures. (C-D) Eye discs undergoing *Rae1* RNAi anterior to the MF using *eygal4* (D, *ey>Rae1*^*IRV*^) show increased pHH3 staining (white) compared to control *eygal4/+* disc (C) including posterior to the MF where cells most should be differentiating. (E) Bar graph quantifying the average pHH3 cells per region of the disc; antenna, blue; anterior to the MF, green; posterior to the MF, orange. N = 7 for each genotype. (F) Quantification of pHH3 staining in wing discs undergoing *Rae1* reduction through heterozygosity at the *Rae1* locus (*c5gal4*, *Rae1*^*ex28*^*/+*, S5K Fig), *Rae1* RNAi in the whole wing disc (*c5>Rae1*^*IRV*^, S5L Fig) or both (*c5>Rae1*^*IRV*^, *Rae1*^*ex28*^*/+*, S5M Fig) show an increase in pHH3 positive cells relative to control wing discs (*c5gal4/+*, S5J Fig). N = 8, 5, 10, 4. (G-I) Rae1 knockdown with siRNA (H, pink line in I) increases the doubling time (indicated) of HeLa cells compared to control transfected cells (G, black line in I). (J) Quantification of BrdU incorporation in terms of the mean pixel intensity of equivalent regions in the SMW of control *eygal4/+* discs and *ey>Rae1*^*02*^ discs. N = 4, 5. (K) *ey>Rae1*^*02*^ eye disc stained for pHH3. (K’) Quantifying the number of pHH3 positive cells show increased pHH3 in the antenna (blue) and in the eye anterior to the MF (green) but not in eye posterior to the MF (orange) where cells should be differentiating. N = 7, 9. (L-M) Rae1 over-expression (pink line in L, M) decreases the doubling time (indicated) of 293T (L) and HeLa (M) cells compared to control transfected cells (black line in L, M). * indicates statistically significant change from controls, p<0.05. Scale bars in A-B indicate 25 μm, in C-D and K indicate 50 μm, and in G-H indicate 75 μm.

Tissue undergoing *Rae1* RNAi (GFP-positive) prior to differentiation showed reduced BrdU incorporation most obviously in the SMW compared to adjacent wild-type tissue (GFP-negative), consistent with decreased entry into S-phase (Fig 3A and 3A’ and S5G-S5H’ Fig). This is consistent with a previously reported role for Rae1 in the G1-S transition in cell culture [46]. Despite decreased BrdU incorporation, *Rae1* RNAi clones in the eye disc did not show decreased phospho-histone H3 (pHH3) staining (Figs 3B, 3B’, S5I and S5I’). Reduced BrdU incorporation but no obvious reduction in pHH3 staining is perplexing but suggests that cells undergoing *Rae1* RNAi that do enter the cell cycle endure a prolonged stay or arrest in mitosis.

To further investigate this mitotic phenotype, in addition to constitutive *Rae1* RNAi in small clones, we examined eye discs undergoing *Rae1* RNAi across all cells anterior to the MF using *eygal4*. These discs were smaller than controls but, surprisingly, showed increased pHH3 staining in the antenna, anterior to the MF, and, strikingly, posterior to the MF compared to controls (Fig 3C–3E; because the increased pHH3 staining made it difficult to distinguish the posterior border of the SMW, pHH3 staining was quantified for the regions anterior to the MF versus posterior to the MF including the SMW). Consistent with findings in the eye, we also observed an increase in pHH3-positive cells in larval wing discs (Fig 3F and S5J–S5M Fig) and in S2 cells (S5N Fig) upon Rae1 loss. As noted, cells posterior to the SMW do not divide during the third instar larval stage and therefore should not stain positive for pHH3. We saw no significant BrdU incorporation or inappropriate cycA and cycB staining posterior to the SMW in these discs (S5O–S5R Fig) indicating that (1) these pHH3-positive cells were not actively cycling and (2) presumably they had completed cyclin degradation potentially placing them in anaphase or telophase. Visual examination of nuclei in S2 cells undergoing Rae1 RNAi showed significant abnormalities including multipolar spindles, inappropriately localized tubulin, and lagging chromosomes (S6 Fig), consistent with reports that Rae1 depletion causes disorganized or multipolar spindles as well as chromosome alignment and segregation defects in cultured human and plant cells [42, 59–60] and in *Drosophila* neuroblasts and spermatocytes [47].

The requirement for Rae1 in cellular proliferation is conserved in mammalian cells; transient *Rae1* knockdown in various mammalian transformed and tumorigenic cell lines reproducibly restricted proliferation (Fig 3G–3I and S7A–S7D Fig). These cells have intact p53 signalling and did not show elevated p21 transcription (S7E–S7G Fig), suggesting the proliferative arrest is likely independent of p53.

A decrease in proliferation upon Rae1 loss might indicate a role for Rae1 to promote proliferation. Over-expressing Rae1 in the early eye resulted in increased BrdU incorporation (Fig 3J and S7H and S7I Fig) and increased pHH3 staining anterior to MF (Fig 3K and 3K’) suggesting that Rae1 plays a role to promote proliferation. Analogously, Rae1 over-expression promoted proliferation in both 293T and HeLa cells (Fig 3L and 3M). Taken together, these findings reflect a highly conserved role for Rae1 in proliferation.

### Rae1 regulates cyclin A and cyclin B levels

**Fig 4:**
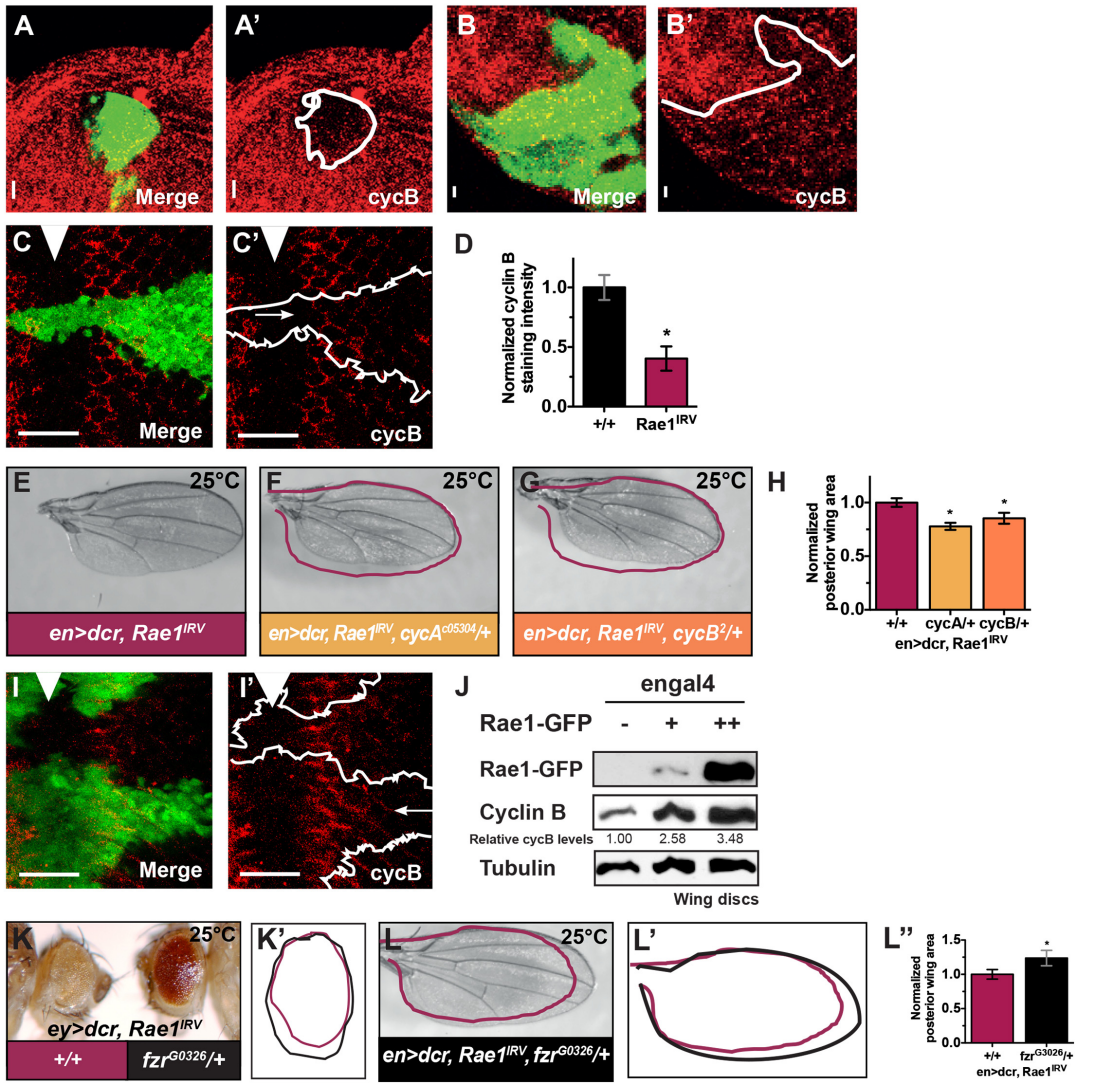
Rae1 regulates cycB. (A-D) Clones undergoing *Rae1* RNAi (green in A, B, C and tracings in A’, B’, and C’) show reduced cycB staining (red A-C’) in the antenna (A-A’), the anterior region of the eye disc (B-B’) and in the SMW (C-C’). Quantification of fluorescence intensity in clones from a several discs show reduction in cycB staining by at least 30%. (D) Quantification of cycB staining intensity based on average intensity from 5 of equivalent regions in control tissue (black bar) and the Rae1 RNAi clones (pink bar) of the clones shown in B-B’. (E-H) Mutations in *cycA* (F) and *cycB* (G) dominantly enhance reduced wing size of RNAi to *Rae1* in the posterior wing, highlighted by traced overlay of control wing from (E) (pink) and quantified in H. For graph in H, N = 4, 7, 7. (I) Rae1 over-expressing clones (green in I, tracing in I’) show increased cycB staining (red). Arrow in I’ indicates high cycB staining extending more posterior in Rae1 over-expressing clone compared to adjacent wild-type clone. (J) Lysates of wing discs over-expressing Rae1 in the posterior compartment show a dose-dependent increase in cyclin B protein levels. (K-K’) Mutation in *Cdh1/fzr* (right, black tracing in K’) dominantly suppressed the reduced eye size from *Rae1* RNAi in the early eye (left in K, pink tracing in K’). (L-L”) Mutation in *Cdh1/fzr* dominantly suppressed the reduced wing size upon *Rae1* RNAi in the posterior wing (red overlay shows a tracing of control wing from E). (L’- L”) overlaid tracings (L’) and quantification (L”) of wings from E, L. For graph in L”, N = 4, 4. Relative cycB protein levels (normalized by tubulin loading control) are indicated. *indicates statistically significant difference p<0.05. Scale bars indicate 25 μm.

Previous studies reported genetic interactions between *wts* and *cycA* to regulate organ size [32], and *wts* loss affected cycE, cycA, and cycB levels (S8A and S8B Fig). Although accumulation of cycA and cycB has been reported in a variety of Hippo Pathway mutants, the functional mechanism underlying their regulation by Hippo signaling has remained unresolved. A prior report linked decreased S-phase entry of Rae1 loss to cycE [58], and regulation of cycE by the Hippo Pathway has already been established. Therefore, to further investigate the Rae1 loss-of-function mitotic phenotypes and establish if they underlie Hippo signaling regulation of mitosis, we proceeded by examining cycA and cycB levels upon Rae1 knockdown. Clones undergoing *Rae1* RNAi anterior to the MF and in the SMW showed subtly decreased cycA and cycB staining compared to adjacent control tissue (Fig 4A–4D and S8C and S8C’ Fig). To establish if decreased cyclin levels were functionally relevant to reduced organ size caused by *Rae1* knockdown, we reduced *cycA* and *cycB* gene dosage. Heterozygous mutation in *cycA* and *cycB* across the fly each dominantly enhanced the reduced organ size phenotypes of Rae1 RNAi in the eye and wing (Fig 4E–4H and S8D–S8G Fig) but did not reduce the size of control wings (S8H Fig). Conversely, individually over-expressing cycE, cycA, or cycB3 partially suppressed reduced eye size caused by reduction in *Rae1* (S8I–S8M Fig) despite producing no overgrowth of control eyes (S8N–S8R Fig).

The reduced cyclin levels upon reduction of Rae1 together with the genetic interaction studies suggest a normal role for Rae1 to positively regulate cycA and cycB levels to promote proliferation and organ size. Consistent with this, cycA and cycB staining increased in Rae1-over-expressing clones and discs (Fig 4I and 4I’ and S8S–S8U Fig). Furthermore, increasing Rae1 in the larval wing disc increased cycB protein levels in a dose-dependent way (Fig 4J).

How does Rae1 regulate cycA and cycB? Rae1 was identified for a role in RNA export [41] and was later reported to inhibit the Anaphase Promoting Complex/Cyclosome (APCC) activator Cdh1/Fizzy-related (*fzr*, also called *rap;* referred to here as *Cdh1/fzr*) in mammalian cells [43–45]. The APCC is a ubiquitin ligase that targets the mitotic cyclins and has an essential role in mitosis. When coupled to substrate-specific activators Cdc20/Fizzy (fzy) and Cdh1/fzr, the APCC ubiquitinates substrates to direct them for proteasomal degradation. Excess Cdh1/Fzr activity upon Rae1 loss could explain reduction of cycA and cycB. To establish if Cdh1/Fzr misregulation played a role in Rae1 organ size phenotypes, we tested interactions with APCC components and regulators. We saw no obvious change in reduced organ size of Rae1 RNAi in the eye by removing one copy of APCC subunit *Cdc27* or *Cdc20/fzy*. However, removing one copy of *Cdh1/fzr* across the fly dominantly suppressed the reduced organ size of *Rae1* RNAi in the eye and wing (Fig 4K–4L”) but did not increase the size of control organs (S8V Fig). Although we cannot rule out parallel regulation of cycB by Cdh1/Fzr-APCC, these findings are consistent with a model that the reduced organ size resulted from specific effects of Rae1 on Cdh1/Fzr-APCC, not Cdc20-APCC.

Proliferation is necessary to achieve appropriate organ size, but changes in cell cycle regulation are not sufficient to increase organ size. For example, loss of cell cycle regulators such as cyclin E (cycE) can be dramatic enough that they cannot be compensated for sufficiently to achieve normal organ size (S8X and S8Y Fig), but over-expression of cycE, cycA, or cycB3 on their own do not increase organ size [32] (S8N–S8R Fig).

### Hippo signaling regulation of Rae1 is important to its restriction of organ size *in vivo*

**Fig 5:**
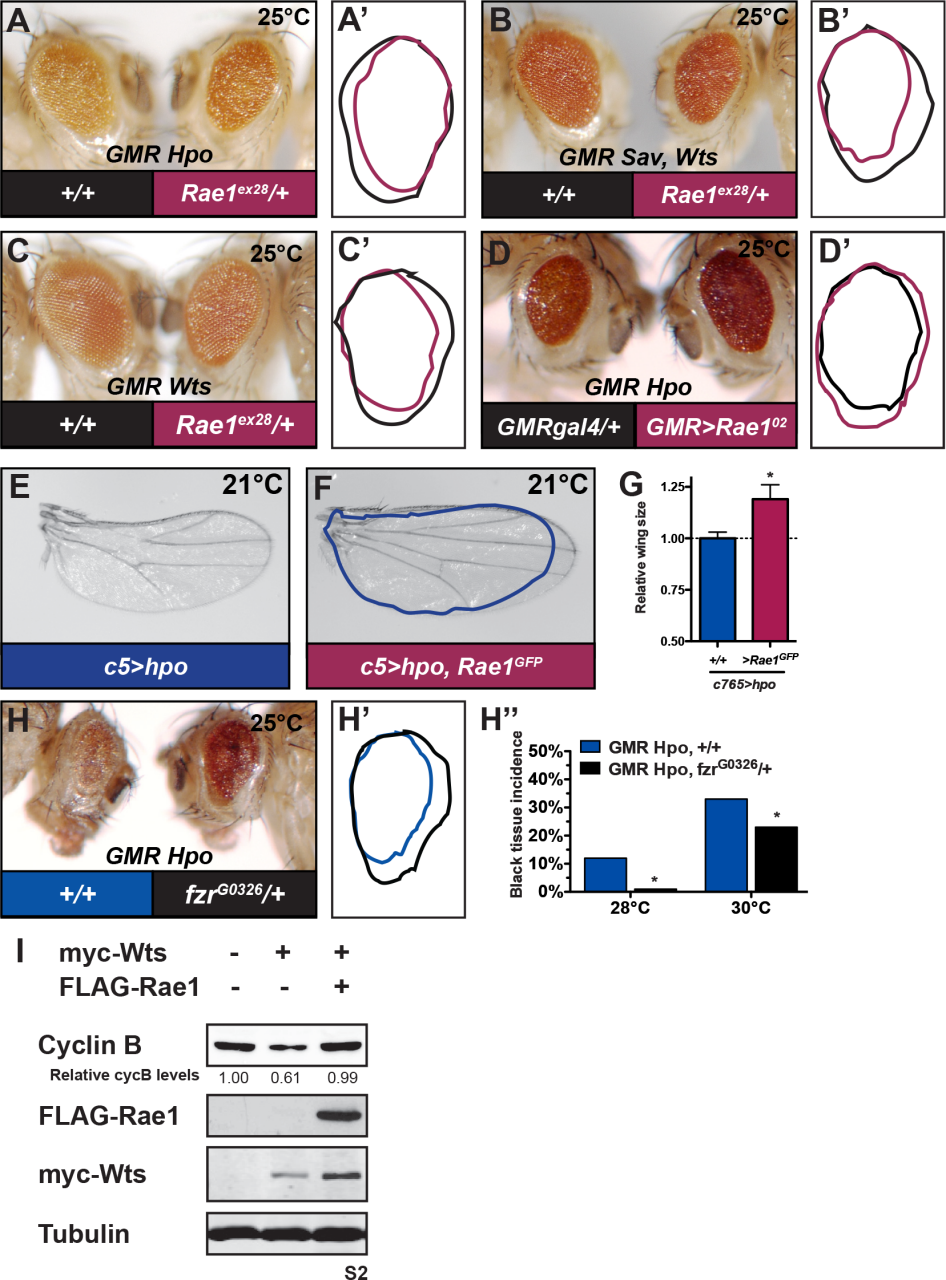
Restriction of Rae1 is important for Hippo signaling restriction of organ size and downregulation of cyclin B. (A) Removing one copy of *Rae1* by introducing deletion allele *Rae1*^*ex28*^ (right eye in A, pink tracing in A’) dramatically enhances the phenotype of expressing Hpo in differentiating eye cells, *GMR Hpo*, (left eye in A, black tracing in A’) evident by increased roughness, and further reduced eye size. (B) *Rae1*^*ex28*^ (right eye in B, pink tracing in B’) dramatically enhances the phenotype of over-expressing Sav and Wts together in differentiating eye cells, *GMR Sav*,*Wts*, (left eye in B, black tracing in B’). (C) *Rae1*^*ex28*^ (right eye in C, pink tracing in C’) dramatically enhances the phenotype of over-expressing Wts in differentiating eye cells, *GMR Wts*, (left eye in C, black tracing in C’). (D) Over-expressing Rae1 in differentiating eye cells (*GMR>Rae1*^*02*^) causes no obvious phenotype (Fig 2S and 2S’) but dramatically suppresses (right eye in D, pink tracing in D’) the phenotype of *GMR Hpo* (left eye in D, black tracing in D’) evident by restoration of eye size and decreased roughness. (E-G) Over-expressing Hpo in the wing reduces wing size (E, blue tracing in F, quantified in G). (F) Over-expressing Rae1 restores wing size (quantified in G). For graph in G, N = 9, 12. (H-H”) Mutation in *Cdh1/fzr* (right, black tracing in H’) dominantly suppresses reduced eye size of *GMR Hpo* (left in H, blue tracing in H’). (H”) As the phenotype of *GMR Hpo* increases in severity, black tissue appears, as shown in S9A–S9D Fig. Removing one copy of *Cdh1/fzr* (black bars, *GMR Hpo*, *fzr*^*G3026*^*/+*) suppresses the appearance of black tissue in *GMR Hpo* eyes (blue bars) at 28°C and 30°C. N = 60, 85, 222, 26. (I) Wts over-expression in S2 cells reduces cycB protein levels (lane 2) compared to controls (lane 1). Concomitant over-expression of a low-level of FLAG-Rae1 restores cycB levels (lane 3) to control levels. Relative levels of cycB (normalized to Tubulin) are indicated for blot in I. * indicates statistically significant difference p<0.05.

Over-expressing Hpo, Sav and Wts, or Wts alone in differentiating cells of the *Drosophila* eye reduces eye size. Eyes become smaller and rougher and black tissue appears with increased expression of Hpo (S9A–S9D Fig); these phenotypes are suppressed by reducing *wts* gene dosage (S9E–S9G Fig). If these phenotypes result in part by promoting Rae1 degradation, they would be enhanced by further reducing Rae1 and suppressed by restoring Rae1 levels. Removing one copy of Rae1 on its own (with *Rae1*^*ex28*^) or with a deficiency that uncovers it (*Df(2R)ED3923* or by *Rae1* RNAi in differentiating eye cells (*GMR>Rae1*^*IRV*^) resulted in no obvious phenotype (Fig 2L and 2L’) but enhanced the phenotype of over-expressing Hpo (*GMR Hpo*, Fig 5A–5A’ and S9H–S9J’ Fig), Sav and Wts (*GMR Sav*, *Wts*, Fig 5B and 5B’ and S9K–S9K’ Fig), and Wts alone (*GMR Wts*, Fig 5C–5C’) in differentiating eye cells in terms of both eye size and the appearance of black tissue. In contrast, co-over-expressing Rae1 in differentiating eye cells (*GMR>Rae1*^*02*^) or constitutively (*Act>*Rae1GFP) strongly suppressed the small eye caused by *GMR Hpo* (Fig 5D and 5D’ and S9L and S9L’ Fig) but resulted in no obvious phenotype on its own (Fig 2S and 2S’). Similarly, the small wing phenotype caused by Hpo over-expression in the wing was suppressed by reducing the gene dosage of *wts* or by Rae1 over-expression (Fig 5E–5G and S9M–S9N Fig). These *in vivo* findings are consistent with tissue culture findings that Hippo signaling negatively regulates Rae1 downstream of Wts (Fig 1 and S2 Fig) and provide evidence that this regulation, regardless of direct targeting by Wts or targeting further downstream, plays a role in the Hippo-mediated restriction of organ size.

Generally, RNAi reduces but does not eliminate gene expression; *Rae1* RNAi should lead to Rae1 protein at lower levels subject to its endogenous post-translational regulation. Therefore, reducing the gene dosage of negative regulators of Rae1 protein should suppress *Rae1* RNAi organ size phenotypes. Indeed, mutations in *Mer*, *ex*, *hpo*, or *wts* dominantly restored eye and wing size in organs undergoing *Rae1* RNAi (S10A and S10B Fig and S1 Table for *hpo*, *Mer*, and additional effectors on the eye, and S10C–S10E Fig for *hpo* and *wts* effects on the wing). Together with our *in vitro* findings (Fig 1 and S2 Fig), these genetic interactions support a role for Hippo signaling to negatively regulate Rae1 *in vivo* to restrict organ size.

Hippo Pathway downregulation of Rae1 (Fig 1 and S2 Fig), Rae1 inhibition of Cdh1/Fzr [43–45], and Rae1 interaction with Cdh1/Fzr *in vivo* (Fig 4K–4L”) would further suggest that Hpo promotes Cdh1/Fzr activation by relieving Cdh1/Fzr inhibition by Rae1. Therefore, Hpo over-expression phenotypes may in part result from excess Cdh1/Fzr activity. Indeed, removing one copy of two distinct alleles of *Cdh1/fzr* partially restored *GMR Hpo* eye size (Fig 5H–5H” for *fzr*^*G0326*^, S10F Fig) suggesting a possible functional link between the Hippo Pathway and the essential cell cycle ubiquitin ligase, APCC.

Previous studies reported genetic interactions between *wts* and *cycA* to regulate organ size [32]. We observed that *wts* loss affected cycE, cycA, and cycB levels (S8A and S8B Fig). If the cyclin decreases in the context of Hippo signaling result from Rae1 depletion, then restoring Rae1 levels should prevent Wts-mediated cyclin decrease. In the context of Wts over-expression, expressing a low level of Rae1 restored cycB protein to control levels in S2 cells (Fig 5I).

### Accumulation of Rae1 is important for cycB accumulation and overgrowth phenotypes seen upon Hippo Pathway loss of function

**Fig 6:**
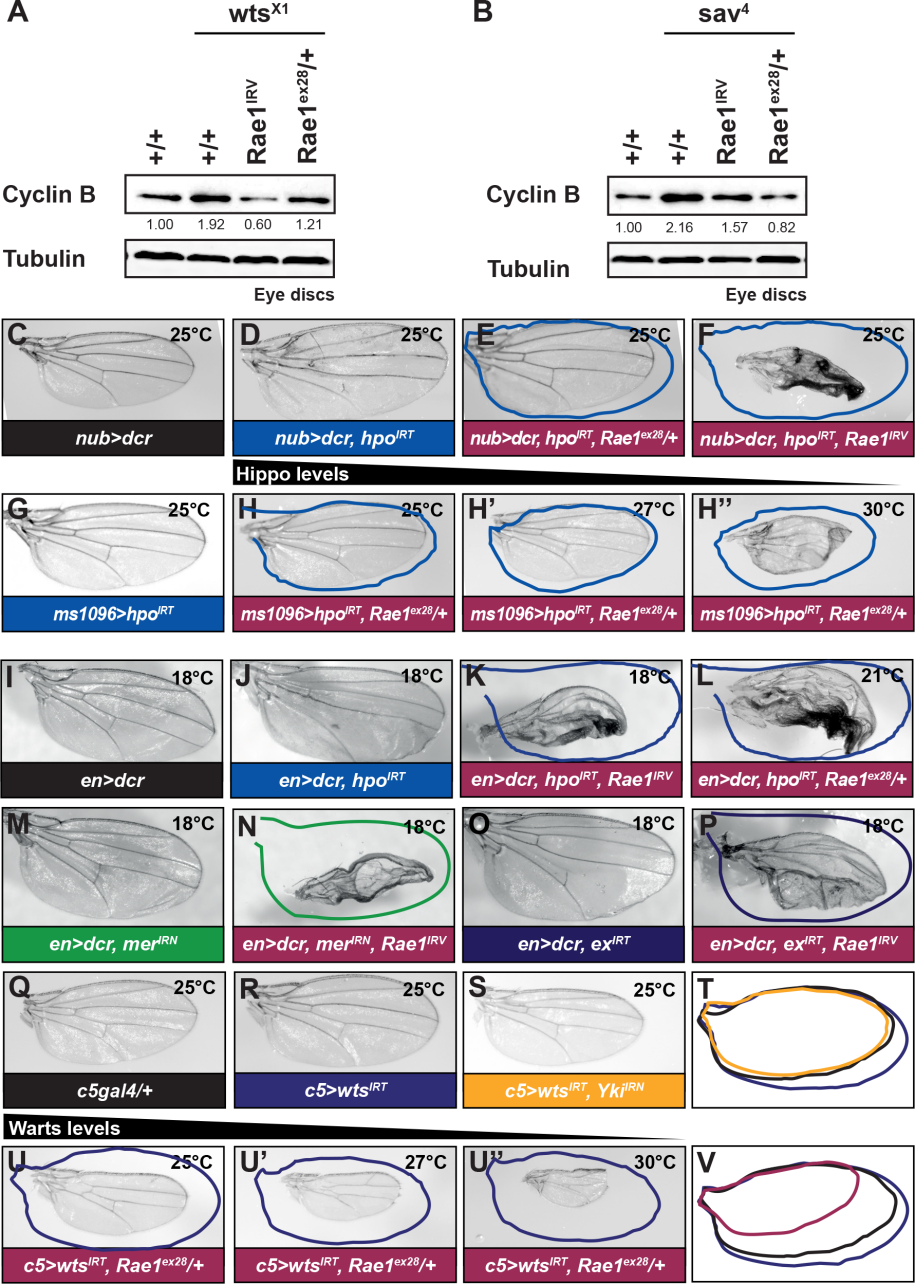
Rae1 is required for cycB accumulation, overgrowth, and survival upon loss of Hippo signaling. (A) Wts loss (MARCM *wts*^*x1*^ clones) in eye discs increases cycB protein levels (lane 2) compared to control *FRT82B* discs (lane 1). Concomitant reduction of *Rae1* either through RNAi (lane 3) or removing a genomic copy (lane 4) restores cycB levels. (B) *Sav* loss (MARCM *sav*^*4*^ clones) in eye discs increases cycB protein levels (lane 2) compared to control *FRT82B* discs (lane 1). Concomitant reduction of *Rae1* either through RNAi (lane 3) or removing a genomic copy (lane 4) restores cycB levels. Relative levels of cycB (normalized to Tubulin) are indicated for blots in A, B. (C) Control wing (*nub>dcr*). (D) RNAi to *hpo* in the wing using *nubgal4* (*nub>dcr*, *hpo*^*IRT*^) causes wing overgrowth. (E-F) Reducing *Rae1* levels slightly by removing one copy suppresses tissue overgrowth (E, *nub>dcr*, *hpo*^*IRT*^, *Rae1*^*ex28*^*/+*) while greater reduction in *Rae1* levels by RNAi both suppresses overgrowth and causes tissue lethality (F, *nub>dcr*, *hpo*^*IRT*^, *Rae1*^*IRV*^). (G) Control wing showing overgrowth due to *hpo* RNAi using the wing driver *ms1096gal4* (*ms1096>hpo*^*IRT*^) at 25°C. (H-H”) Removing one copy of Rae1 using *Rae1*^*ex28*^ (*ms1096>hpo*^*IRT*^, *Rae1*^*ex28*^*/+*) suppresses the overgrowth (H). The gal4/UAS system is temperature responsive. Increasing the temperature leads to increased expression of inverted repeats and increased knockdown of target genes. Increasing the temperature to 27°C (H’) and 30°C (H”) further decreases Hippo signaling by increased RNAi to *hpo*. Blue overlaid tracings show control *ms1096>hpo*^*IRT*^ wing outlines from the indicated temperatures; wings are more overgrown but do not lie flat; overlaid traced images of these wings are smaller than actual wing size. Removing one copy of Rae1 (which maintained the *Rae1* gene dosage to that in H) causes more dramatic tissue loss with further increasing the temperature to 27°C (H’) and 30°C (H”). (I) Control *en>dcr* wing at 18°C. (J) RNAi to *hpo* (*en>dcr*, *hpo*^*IRT*^) in the posterior wing at 18°C; wings are so enlarged that flattened, mounted wings appear smaller than they are; see S12C and S12D Fig for examples of overgrown wings still attached to flies corresponding to the wings in J, K). (K-L) Reducing *Rae1* by concurrent *Rae1* RNAi (*en>dcr*, *hpo*^*IRT*^, *Rae1*^*IRV*^, K), or introducing *Rae1*^*ex28*^ (L) suppresses overgrowth and causes overgrowing wings to blacken and shrivel (tracing of a control *en>dcr*, *hpo*^*IRT*^ wing is overlaid to highlight growth suppression). (M-P) RNAi to *Rae1* causes growth suppression and tissue disruption upon concurrent RNAi to *Mer* (*en>dcr*, *mer*^*IRN*^, *Rae1*^*IRV*^, N) compared to RNAi to *Mer* alone (*en>dcr*, *mer*^*IRN*^, *Rae1*^*IRV*^, M, green tracing in N) or concurrent RNAi to *ex* (*en>dcr*, *ex*^*IRT*^, *Rae1*^*IRV*^, P) compared to RNAi to *ex* alone (*en>dcr*, *ex*^*IRT*^, O, purple tracing in P). Overgrowth due to *wts* RNAi with *engal4* was so extensive wings were too overgrown to mount for comparison. (Q) Control wing (*c5gal4/+*). (R) RNAi to *wts* in the wing using *c5gal4* (*c5>wts*^*IRT*^) causes wing overgrowth (purple tracing in T, U, and V). (S) RNAi to *yki* (*c5>wts*^*IRT*^, *yik*^*IRN*^, yellow tracing in T) suppresses tissue overgrowth, restoring wing size to that of a control wing (black tracing). (U-V) In contrast to *yki* RNAi in S-T, removing one copy of *Rae1* using *Rae1*^*ex28*^ not only suppresses overgrowth but promotes significant tissue loss (*c5>wts*^*IRT*^, *Rae1*^*ex28*^*/+*, U, pink tracing in V). The resulting wings are significantly smaller than either control wings (Q) or wings suppressed by *yki* RNAi (S); this is highlighted by an overlay of tracings in (V). Increasing the temperature to 27°C (U’) and 30°C (U”) further decreases Hippo signaling by increased RNAi to *wts*. Blue overlaid tracings show control *c5>wts*^*IRT*^ wing outlines from the indicated temperatures; as noted earlier, overgrowth in these context is quite dramatic such that wings no longer lie flat; mounted wings thus wrongly appear smaller once they are flattened to be photographed. Under these conditions, removing one copy of *Rae1* (which maintained the *Rae1* gene dosage to that in U) causes even more dramatic loss of tissue.

By the logic above, if accumulation of the cyclins in the absence of Hippo signaling resulted from Rae1 accumulation, then reducing Rae1 levels in those contexts should suppress cyclin accumulation phenotypes. As explained shortly, it is difficult to perform epistatic analysis with double mutant tissue, so we were limited in the contexts in which to perform epistasis experiments. Normal cyclin levels were restored in homozygous mutant *wts* or *sav* tissue in eye discs (generated using MARCM tools) upon reducing *Rae1* (by low level *Rae1* RNAi or removing one copy of *Rae1*) (Fig 6A and 6B). Together with the Wts-over-expression studies in Fig 5I, these findings indicate that the Wts regulation of cycB occurs through the downregulation of Rae1.

Knocking down *Mer*, *ex*, *hpo*, or *wts* in the posterior or whole wing or inhibiting Hippo signaling with a kinase dead version of Hpo resulted in dramatic wing overgrowth (Fig 6D, 6G, 6J, 6M, 6O and 6R and S11C, S11G, S11N, S11R, S12A, S12C, S12E and S12H Figs) compared to controls (Fig 6C, 6I and 6Q and S11A, S11E, S11L, S11P and S12G Figs). Reducing the levels of critical target Yki suppresses this overgrowth ([26]; Fig 6S and S12F and S12J Fig). Similarly, in contexts of little overgrowth, low-level *Rae1* RNAi causing mild or no change in wing size on its own or removing one copy of *Rae1* significantly reduced overgrowth due to loss *hpo* or over-expression of kinase dead Hpo (Fig 6E and 6H and S11D, S11I and S11J Fig). This suggests that the accumulation of Rae1 upon loss of Hippo signaling (Fig 1C and 1D and S2B, S2D and S2E Fig) is important for the resulting tissue overgrowth.

### Tissue overgrowing due to loss of *Mer*, *ex*, *hpo*, or *wts* but not Yki-over-expression requires Rae1 for tissue survival

Surprisingly, reducing *Rae1* gene dosage in many of these overgrowth contexts also gave rise to shriveled and blackened wings, a “tissue synthetic lethality” phenotype. As noted above, in contexts of little overgrowth, *Rae1* knockdown suppressed overgrowth (Fig 6E, 6H and 6J and S11D, S11I and S11J Fig) or led to blistering (S12B Fig). Upon much lower levels of Hippo Pathway activity that causes more severe overgrowth, *Rae1* knockdown caused catastrophic tissue loss (Fig 6F, 6H”, 6K–6L, 6N, 6P, 6U and 6U”; S11H, S11I’ and S11J’ Fig). Enhanced overgrowth can cause tissue collapse; however, in these cases overall wing size decreased coincident to tissue collapse, reflecting suppressed not enhanced overgrowth. This is also highlighted when we reduce *hpo*, *ex*, or *Mer* in only the posterior compartment using *engal4* (Fig 6K–6L, 6N and 6P and S12B and S12D Fig); anterior tissue remains intact indicating that the wings inflated and only posterior tissue was collapsing. We saw tissue loss throughout the body including in the thorax, the legs, and the eye when we created random clones in proliferating tissue (S12K Fig for the eye) and compelling growth suppression and tissue loss using multiple gal4 drivers including *nubgal4* (Fig 6C–6F), *ms1096gal4* (Fig 6G–6H”‘), *engal4* (Fig 6I–6P and S12A–S12D Fig), and *c765gal4* (referred to as *c5gal4* Fig 6Q–6V). We did not observe catastrophic tissue loss when using *GMRgal4*, suggesting this phenomenon may reflect sensitivity of proliferating, not differentiated, tissue. Importantly, this synthetic tissue lethality upon reducing Rae1 in the context of limited Hippo Pathway activity is characteristically distinct from Yki, reduction of which suppressed overgrowth upon loss of Hippo signaling but did not compromise the survival of overgrowing tissue (Fig 6S and S12F and S12J Fig).

Tissue lethality instead of straight-forward suppression was unanticipated because (1) we reduced *Rae1* to a level with minimal or no phenotypes on its own (removing one copy or low-level RNAi), (2) even significant knockdown or knockout of *Rae1* does not cause tissue lethality, and (3) *Rae1* reduction would be expected only to decrease proliferation and decrease organ size. When lethality results in genetic interactions upon modulating two genes each of whose individual modulation is not lethal, the term”synthetic lethality” is applied and usually results from perturbing genes with parallel, redundant roles or between genes of the same pathway. The “tissue synthetic lethality” was specific for Hippo signaling and not a global response of overgrowing tissue. We did not see tissue lethality upon *Rae1* RNAi or removing one copy of *Rae1* with expression of oncogenic Ras, myc, or caspase inhibitor p35 (S12L–S12O Fig for Myc and p35). The “synthetic lethality” in this instance, therefore, might suggest that tissue with impaired Hippo signaling requires Rae1 for survival and further supports a role for Rae1 in Hippo signaling.

**Fig 7:**
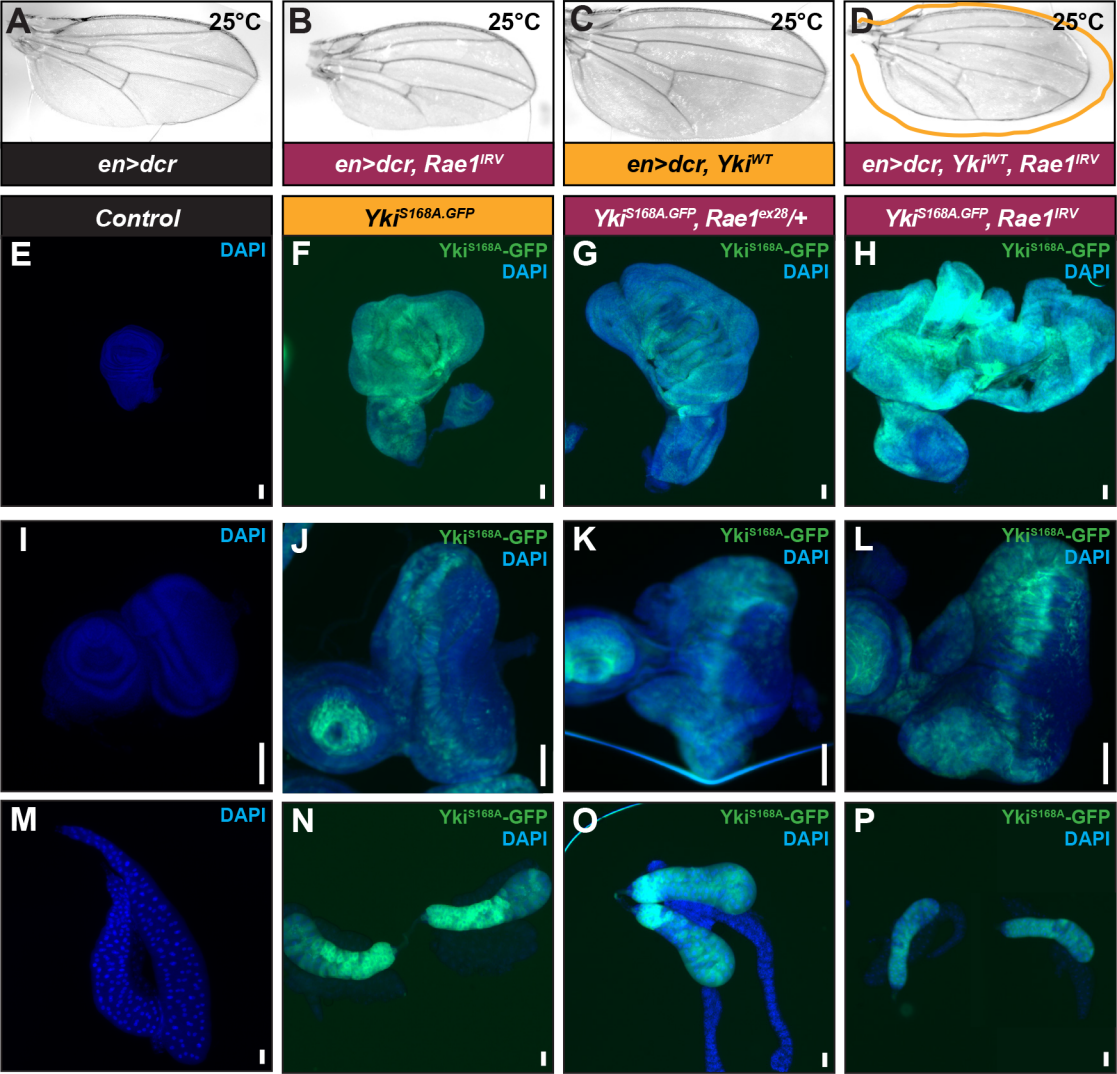
Rae1 negatively regulates Yki/YAP in *Drosophila* and in mammalian cells. (A) Control wing (engal4/+). (C) Over-expressing Yki leads to wing overgrowth (C, and yellow overlay in D). (D) RNAi to *Rae1* even to levels that reduce wing size (B) suppressed Yki-mediated overgrowth but did not cause tissue collapse (D). (E) Control wing disc (*c5gal4/+*). (F) Over-expressing Yki leads to wing disc overgrowth (*c5>Yki*^*S168A*^). (G) Reducing *Rae1* levels slightly by removing one copy enhances wing overgrowth. (H) Reducing *Rae1* levels by concurrent *Rae1* RNAi (*c5>Yki*^*S168A*^, *Rae1*^*IRV*^) further increases wing disc size. (I) Control eye disc (*eygal4/+*). (J) Over-expressing Yki leads to eye disc overgrowth (*ey>Yki*^*S168A*^). (K) Reducing *Rae1* levels slightly by removing one copy enhances eye disc overgrowth. (L) Reducing *Rae1* levels by concurrent *Rae1* RNAi (*ey>Yki*^*S168A*^, *Rae1*^*IRV*^) further increases eye disc size. (M) Control salivary gland (*ptcgal4/+*). (N) Over-expressing Yki leads to a reduction in salivary gland size (*ptc>Yki*^*S168A*^). (O) Reducing *Rae1* levels slightly by removing one copy enhances the small salivary gland size. (P) Reducing *Rae1* levels by concurrent *Rae1* RNAi (*ptc>Yki*^*S168A*^, *Rae1*^*IRV*^) further decreases salivary gland size. Glands in 7P were separated during dissection; gland on the right was overlaid into the frame with the gland on the left to provide images of two glands. Scale bars indicate 100 μm.

To determine whether Rae1 could promote “synthetic lethality” of Yki-over-expressing tissue, we reduced *Rae1* levels in a range of Yki over-expression contexts including contexts that matched or exceeded the extent of overgrowth seen for reducing *Mer*, *ex*, *hpo*, and *wts*. When Yki caused moderate overgrowth matching those shown for loss of *Mer*, *ex*, *hpo*, or *wts*, *Rae1* reduction in some cases suppressed the wing overgrowth but did not cause tissue ablation (Fig 7A–7D and S13A–S13R Fig). The lack of tissue collapse of *Rae1* knockdown upon Yki over-expression emphasizes that there is a fundamental difference between loss of Hippo signaling and Yki over-activation and that the “tissue synthetic lethality” phenomenon is restricted to specific components of the pathway including *Mer*, *ex*, *hpo*, and *wts*.

### Rae1 regulates Yki/YAP levels, localization, and activity

In cases of Yki over-expression where there was even more dramatic overgrowth, *Rae1* reduction did not suppress the overgrowth but unexpectedly enhanced Yki over-expression adult phenotypes (S13D and S13S–S13T’ Fig). We used a moderately activated transgene (Yki^S168A^) to determine whether the enhancement caused by Rae1 loss was more robust with a higher threshold of Yki activity than that caused by wild-type Yki over-expression. Importantly, this moderately activated form of Yki, Yki^S168A^, is still responsive to Hippo Pathway regulation since co-expression of Hpo and Wts reduces the size of Yki^S168A^ clones and suppresses Yki-mediated eye overgrowth [61]. Because this transgene causes adult lethality with promoters that drive expression in proliferative tissues, we analyzed interactions between Rae1 and Yki^S168A^ in carefully-staged larval organs. Removing a genomic copy of Rae1 dramatically enhanced the Yki-mediated overgrowth phenotypes in imaginal discs (shown for wing and eye discs in Fig 7E–7G and 7I–7K but also seen in leg imaginal discs). Rae1 knockdown using RNAi further enhanced these phenotypes (Fig 7H and 7L). In the salivary glands, Yki^S168A^ mis-expression restricted salivary gland size (Fig 7M and 7N as reported previously, [62]) which was enhanced with Rae1 loss (Fig 7O and 7P).

**Fig 8:**
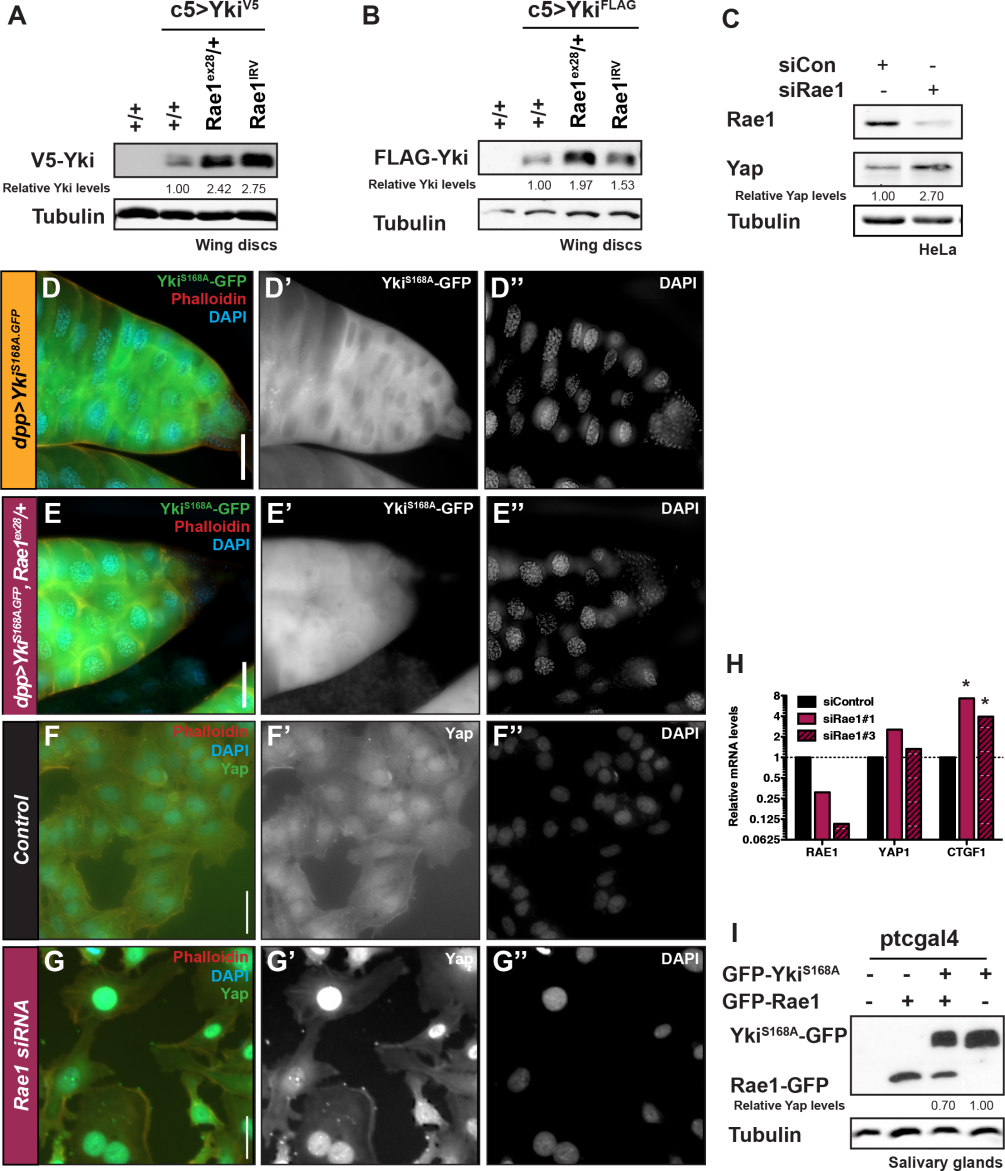
Rae1 is required to restrict Yki/YAP levels and localization. (A) Reducing *Rae1* levels slightly by removing one copy (lane 3) or by RNAi (lane 4) increases Yki-V5 levels in larval wing discs (lane 2). (B) Reducing *Rae1* levels slightly by removing one copy (lane 3) or by RNAi (lane 4) increases FLAG-Yki levels in larval wing discs (lane 2). (C) Rae1 reduction by siRNA increases YAP levels in HeLa cells. (D-D”) Mis-expressing an activated Yki transgene (Yki^S168A^-GFP) in the larval salivary glands shows a predominantly cytoplasmic localization. (E-E”) Mis-expressing activated Yki (Yki^S168A^-GFP) with *Rae1* reduction in the larval salivary glands shows some nuclear localization of Yki. (F-G) Rae1 reduction by siRNA (G) increases YAP nuclear localization compared to control MCF10A cells (F). (H) *Rae1* reduction by siRNA increases the relative mRNA levels (normalized to GAPDH) of YAP target *CTGF* [52] in HeLa cells. *indicates statistically significant change from controls, p<0.05. (I) Expression of Rae1-GFP reduces the levels of Yki^S168A^-GFP (lane 3) compared to control (lane 4) in *Drosophila* salivary glands. Relative levels of Yki in A, B, I and YAP in C (normalized by Tubulin) are indicated. Scale bars indicate 50 μm.

In addition to their changes in size, these tissues showed stronger Yki^S168A^ fluorescence upon Rae1 reduction (for example, the disc in Fig 7H compared to 7F), suggesting that Rae1 may negatively affect Yki protein levels. Rae1 reduction reproducibly increased the levels of both V5 and FLAG-tagged wild-type Yki in wing disc lysates (Fig 8A and 8B). In salivary glands, mis-expressed Yki migrated as a doublet, presumably because of phosphorylation mediated by Hippo Pathway activity (reported to be high in the salivary glands [62]). Rae1 loss caused an increase in Yki levels and reduced the proportion of the slower-migrating form (Fig 8 and S13U Fig). Effects on Yki were conserved in mammalian cells; HeLa cells knocked down for Rae1 showed increased YAP levels compared to control-treated cells (Fig 8C). Consistent with studies of Yki localization in the wing [28], immunofluorescence of Yki mis-expressing salivary glands showed that both wild-type and Yki^S168A^ are predominantly cytoplasmic (Fig 8D–8D” for Yki^S168A^). Rae1 loss promoted nuclear localization of Yki^S168A^ (Fig 8E–8E”). This restriction of Yki localization is conserved in mammalian cells; Rae1 knockdown promoted accumulation and nuclear localization of YAP in transformed, non-tumorigenic mammary epithelial cells (Fig 8G–8G”) compared to control cells (Fig 8F–8F”). Furthermore, this YAP accumulation and relocalization promotes YAP transcriptional activity (Fig 8H). The increase in Yki/YAP protein levels upon loss of Rae1 suggests that Rae1 plays a role to limit Yki/YAP levels. Consistent with this, Rae1 over-expression reduced mis-expressed Yki protein levels (Fig 8I).

### Rae1 feeds back to regulate upstream Hippo Pathway components Mer, Hpo, and Wts

Given that a genome-wide mass-spec study reported direct binding between Rae1 and Yki [63], we cannot exclude that some effects of Rae1 on Yki/YAP might be mediated by direct interactions. However, in experiments exploring the role of Rae1 in Wts-mediated cyclin regulation, we had observed that increased Rae1 levels stabilized myc-Wts (Fig 5I). Because post-translational effects on Yki/YAP levels, localization, and activity are known to result from targeting by the Hippo Pathway [26–28, 61], the Rae1 effects on Wts protein in Fig 5I could explain the regulation of Yki/YAP. This raises the possibility that in addition to serving as a target of the pathway, Rae1 could act in a feedback circuit to promote Hippo Pathway activity at a step upstream of Yki/YAP.

**Fig 9:**
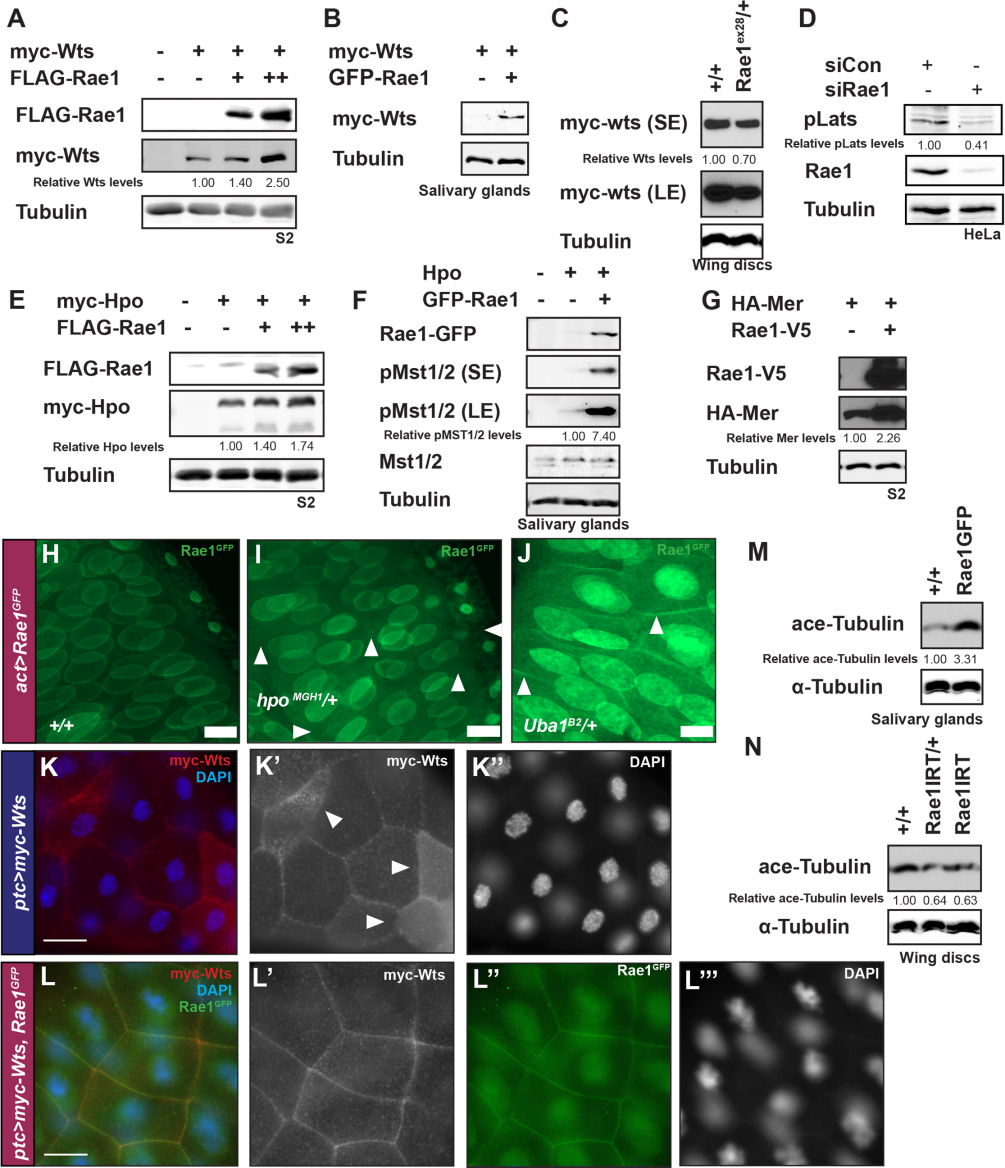
Rae1 feeds back to stabilize Warts, Hippo, and Merlin. (A) FLAG-Rae1 over-expression shows a dose-dependent increase in myc-Wts levels in S2 cells. (B) Rae1 over-expression increases myc-Wts levels (lane 2) compared to control (lane 1) in *Drosophila* salivary glands. (C) Reducing *Rae1* levels slightly by removing one copy (lane 2) reduces myc-Wts levels relative to control (lane 1) in *Drosophila* wing discs. (D) *Rae1* reduction by siRNA reduces pLats levels in HeLa cells. (E) FLAG-Rae1 over-expression shows a dose-dependent increase in myc-Hpo levels in S2 cells. (F) Rae1 over-expression increases pHpo levels (as monitored by pMST/2 antibodies that crossreact with *Drosophila* Hpo, [51]) (lane 3) compared to control (lane 2) in *Drosophila* salivary glands. (G) FLAG-Rae1 over-expression increases HA-Mer levels in S2 cells. (H) Constitutive, low level Rae1-GFP over-expression shows association with the nuclear periphery in salivary glands. (I-J) Increasing Rae1-GFP levels using alleles that impair Hippo signaling (*hpo*^*MGH1*^/+, I) or ubiquitination (*Uba1*^*B1*^/+, J) show increased pools of Rae1-GFP and some association with the cell membrane (arrowheads in I, J) strongest at higher levels of Rae1 (J). All images (I-J) were taken with identical exposure times and settings. Scale bars in H-J indicate 20 μm. (K-K”) Immunofluorescence of Wts over-expression in the salivary glands (*ptc>myc-wts)* shows Wts association with plasma membranes and diffuse staining in the cytoplasm. Arrowheads point to cells with higher levels of cytoplasmic Wts staining. (L-L”‘) Co-over-expression of Rae1 and Wts in the salivary glands (*ptc>myc-wts*, *Rae1*^*GFP*^*)* increases Wts levels and association with the plasma membrane including overlapping domains with Rae1. Cells with higher cytoplasmic Wts staining are no longer seen. Membrane recruitment of Wts has been reported to promote its activity [64–65]. Scale bars in K-L indicate 50 μm. (M) Rae1 over-expression increases acetylated tubulin levels in *Drosophila* salivary glands. (N) Reducing Rae1 levels decreases acetylated tubulin levels in *Drosophila* wing discs. Relative levels of Wts in A-C, pLats in D, Hpo in E, pHpo in F, Mer in G, and acetylated tubulin in M, N (normalized by Tubulin) are indicated.

To investigate the potential for Rae1 to regulate upstream components, we examined their levels and activity *in vitro* and *in vivo*. Rae1 over-expression increased Wts protein levels in S2 cells and *Drosophila* tissue (Fig 9A and 9B and Fig 5I). Conversely, *Rae1* loss reduced Wts protein levels in *Drosophila* tissue (Fig 9C) and reduced Lats1 activation in mammalian cells (Fig 9D), indicating that Rae1 regulation of Wts/Lats is conserved. Rae1 over-expression increased Hpo and Mer protein levels in S2 cells and Hpo activation in *Drosophila* tissue (Fig 9E–9G), suggesting that Rae1 might act at or upstream of Mer, or on multiple components when in complex together. Because Rae1 did not promote accumulation of other proteins tested including GFP (Fig 1E) and promoted reduction in Yorkie/YAP levels (Fig 8I), effects on Mer, Hpo, and Wts protein levels are unlikely due to a non-specific effect to stabilize all proteins.

**Fig 10:**
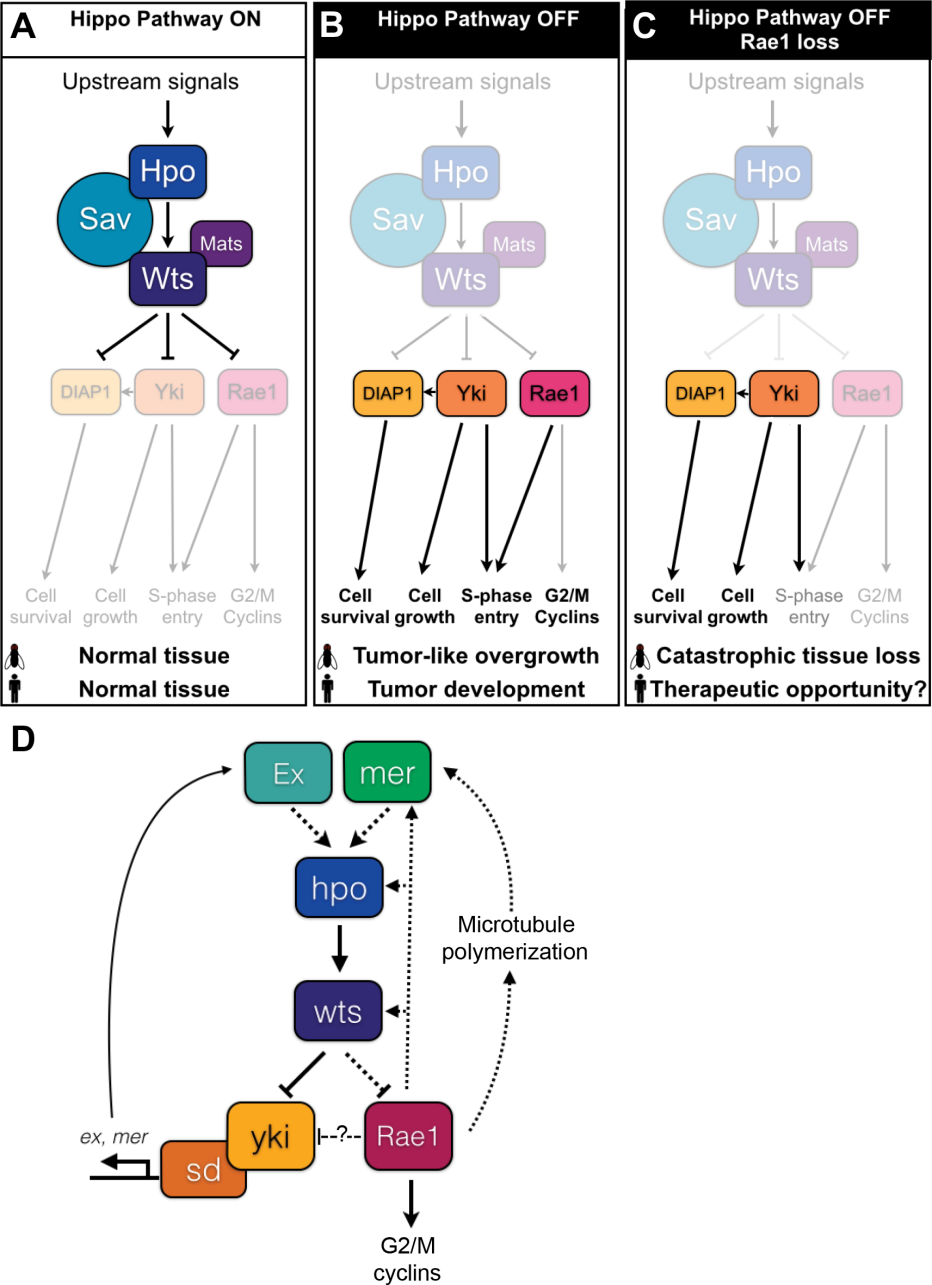
Extended model of Hippo signaling. (A-D) We propose a revised Hippo Pathway model. The schematics show a simplified model of *Drosophila* Hippo Pathway core components Hpo, Sav, Mats, and Wts and downstream targets DIAP1, Yki, and Rae1. Due to the complexity of the Hippo signaling network, not all upstream regulators, downstream targets, or crosstalk with other pathways are pictured. (A) When the Hippo Pathway is active (“ON”), DIAP1 is down-regulated transcriptionally due to Yki inactivation [26] and post-translationally by direct phosphorylation by the core cassette [19, 21]. Inactivation of Yki restricts cell growth (for example, due to decreased *bantam* (*ban*) transcription [70] and *myc* transcription [71–72], not pictured) and impairs S-phase entry by decreasing cyclin E transcription [26]. Down-regulation of Rae1 impairs S-phase entry (Fig 3A) and mitotic progression in part by promoting cycA and cycB loss (Fig 4). (B) When the Hippo Pathway is inactive (“OFF”), Yki promotes increased *ban* [70] and *myc* [71–72] (not pictured) and DIAP1 [26] transcription to promote growth and survival. DIAP1 levels increase further to promote cell death resistance due to the absence of de-stabilizing phosphorylation [19, 21]. Rae1 accumulates due to absence of Wts-mediated downregulation (Fig 1 and S2 Fig) and together with the Yki-dependent increased cyclin E promotes S-phase entry. Rae1 promotes accumulation of cycA and cycB to promote proliferation (Figs 3 and 4). (C) When Hippo signaling is lost but Rae1 levels are not sufficient to meet the demands of the overgrowing tissue (“OFF Rae1 loss”), the subsequent imbalance causes catastrophic tissue loss (Fig 6). (D) We propose a possible model of a Rae1 feedback circuit to explain Rae1 regulation of Yki activity. Upstream components Mer and Ex promote activation of the core kinase cassette [29]. The core cassette then inhibits Yki and Rae1. Ex also plays a role to inhibit Yki by direct binding (not pictured) [73–74]. When active, Yki promotes transcription of *mer* and *ex* to promote pathway feedback. Dotted lines in this simplified, conceptual model represent possible interactions between the Hippo signaling components tested in Fig 9 as candidate targets of feedback from Rae1. Our data (Fig 9) clearly shows a role for Rae1 to regulate Mer, Hpo, and Wts levels and to promote tubulin acetylation either or both of which may explain the Rae1 interactions with Yki (Figs 7 and 8). We cannot rule out a role for Rae1 to inhibit Yki directly (dashed line).

Activation of Hippo signaling requires proper recruitment of the Hpo and Wts kinases to specific regions in the apical membrane from distinct domains by upstream components of the pathway [64–65]. Mer and Ex are membrane-associated proteins that facilitate this activation of Hippo signaling by recruiting Wts/Lats to the membrane where it receives its activating phosphorylation from Hippo/MST [64–65]. To assess how Rae1, a primarily nuclear protein, could affect the protein levels and activation of these components, we looked more closely at Rae1 localization. Consistent with previous reports about Rae1 localization in other systems [42, 59–60, 66], a Rae1-GFP fusion protein was strongly enriched in the nucleus and nuclear periphery in various *Drosophila* tissues (Fig 9H and S14A–S14A” Fig). Rae1 was also found to be associated with a mesh-like network in the cytoplasm. Importantly, at higher levels of Rae1 (resulting from reduced Hpo or Uba1 or from increased Rae1 expression) a pool of Rae1 localized to the membrane (Fig 9I and 9J and S14C–S14C” Fig) whereas at lower levels of Rae1 (such as upon co-expression of Hpo, S14B–S14B” Fig and S14D–S14D” Fig), this pool disappeared. Curiously, in addition to promoting accumulation of Wts protein levels (Figs 5I and 9A and 9B) over-expressing Rae1 increased the membrane association of Wts protein, including some areas of co-localization (Fig 9L and 9L’ compared to Fig 9K and 9K’). Increased Wts at the membrane could reflect the increase in overall Wts levels (Figs 5I and 9A and 9B). Because Mer acts to increase Wts recruitment to the membrane, increased Wts at the membrane could also reflect the increased Mer levels (Fig 9G). Mer and Rae1 both bind microtubules in purified systems [42, 67]. Mer’s association with microtubules is increased upon microtubule acetylation, and interaction with acetylated microtubules is critical in regulating YAP [67–69]. Therefore another potential explanation for the increased Wts recruitment to the membrane could be via Rae1 effects on microtubules or microtubule acetylation which could affect Mer. Rae1 over-expression in *Drosophila* tissues dramatically increased the proportion of acetylated tubulin while tissue undergoing Rae1 RNAi showed reduced acetylated tubulin (Fig 9M and 9N). All together, these findings would be consistent with higher levels of Rae1 activating the Hippo Pathway at the membrane my mutiple mechanisms: (1) Rae1 could act at an upstream step to promote tubulin acetylation to regulate Mer, and/or (2) Rae1 could promote accumulation of core components Mer, Hpo, and Wts which would then promote downregulation of both Rae1 and Yki/YAP (Fig 10).

## Discussion

### The Hippo Pathway promotes Rae1 degradation

The Hippo Tumor Suppressor Pathway plays a highly conserved role from *Drosophila* to mammals in organ homeostasis, in restricting growth and proliferation, and in promoting apoptosis. By searching for post-translational targets, we identified Rae1 as a novel target of Hippo signaling downstream of Wts with a role to regulate proliferation, cycB, and organ size. Our studies showed that Rae1 is regulated by Wts *in vitro* and Wts kinase activity *in vivo*. Whether Rae1 is a direct target of Wts remains an open question. Rae1 binds both Wts and Lats in *Drosophila* and mammalian cultured cells and increasing Hippo activity shows increased recognition of Rae1 by a pRXXS antibody. However, an *in vitro* kinase assay using a peptide encompassing the Wts consensus motif and full-length purified Rae1 showed no phosphorylation. These data raise the possibilities that Rae1 phosphorylation by Wts would require an additional co-factor or a priming phosphorylation, or that Rae1 is targeted downstream of Wts by another kinase. Ongoing studies are centered on resolving how the Hippo Pathway targets Rae1 for degradation.

Unlike DIAP1, which is regulated both transcriptionally by Yki [26] and post-translationally by phosphorylation by the pathway [19, 21], we found no evidence that Rae1 transcript or protein levels are regulated by Yki in *Drosophila* tissues. This is consistent with previous RNA-seq and microarray studies that showed no major changes in *Rae1* mRNA in Yki over-expressing or *wts* mutant contexts [75–77]. Our studies in mammalian cell lines also show no evidence for *Rae1* transcriptional regulation by YAP activity. Consistent with this, microarray studies of activated YAP [9, 52, 78–81] as well as ChIP-on-chip, ChIP-seq and RNA-seq studies [52, 81–83] did not show statistically significant regulation of *Rae1* mRNA in a variety of mouse tissues and human cell lines. Rae1 regulation thus may represent another example of Yki-independent functions of the Hippo pathway including a variety of developmental processes such as dendritic tiling [84], planar polarity [85], salivary gland and neuronal autophagy [62, 86] as well as in growth control contexts including that elicited by F-actin accumulation [87] and alcohol [88], the mechanisms of which remain unresolved. Recent work also identified novel Yki-independent pathway effectors such as Enabled, Mud, and Canoe in processes such as collective migration [89], and spindle orientation [90–91].

### Rae1 regulates cyclin B downstream of Wts

Phenotypic characterization revealed that Rae1 acts as a regulator of organ and organism size and as a critical regulator of mitosis. While both loss-of-function and gain-of-function studies showed increased mitotic index, cells in the former context were not actively cycling and each context produced distinct outcomes in terms of cyclin levels suggesting that Rae1 normally acts to promote mitotic progression and its loss results in a prolonged stay or arrest in mitosis. Consistent with our findings, synchronization experiments in BY-2 plant cells showed that Rae1 depleted cells entered mitosis normally but showed delayed progression [59]. This mitotic phenotype may result from mitotic cyclin dysregulation. We observed that the mitotic cells knocked down for Rae1 did not stain for cycA and cycB, consistent with arrest in anaphase or telophase.

Rae1 loss of function results in decreased cycA and cycB levels, while Rae1 over-expression promotes high cycA and cycB protein levels. Our genetic interaction studies show that the restriction of cycA and cycB levels is critical for Rae1 loss-of-function reduced organ size phenotypes. Furthermore, Rae1 acts epistatically to *wts* and *sav* loss of function in regulating cyclin protein levels. In this work we thus established a Hippo Pathway-dependent but Yki-independent role for Rae1 in mitotic cyclin regulation. A summary of the similarities and differences between the roles and phenotypes of Rae1 and Yki is shown in S2 Table. How could Rae1 regulate the mitotic cyclins? Rae1 has been reported to regulate APCC activator Cdh1/fzr [43–45]. Indeed, we showed that Cdh1/fzr genetically interacts with both Rae1 and Hippo to regulate organ size. Not necessarily mutually exclusive, Rae1 is an RNA binding protein and may directly regulate cycB transcripts, suggested by studies in plants and yeast [62, 92]. Thirdly, mass spec screens in yeast identified binding between Rae1 homolog Gle2 and B-type cyclins CLB2 and CLB3 [93] so may affect cycB by direct protein-protein interaction.

As noted, “organ size checkpoint” mechanisms normally compensate for changes in proliferation to ensure that final organ size is not altered. Rae1 over-expression increased both proliferation and organ size suggesting an exciting role for Rae1 in the Hippo signaling network to integrate regulation of proliferation and overall organ size. We propose that Rae1 acts as a “rheostat” for organ size rather than an “on/off” switch for tissue growth: decreasing Rae1 levels tunes the dial down to a lower organ size while increasing Rae levels tunes the dial up to a larger organ size. However, increased cyclin levels are insufficient to increase organ size. It will be interesting for future studies to investigate which Rae1-dependent processes mediate increased organ size.

### A Rae1-Hippo Pathway feedback circuit

Our studies show that Rae1 restricts Yki levels and localization in *Drosophila* tissues and YAP localization, levels, and activity in mammalian cells, potentially as a result of Rae1-mediated functional regulation of upstream components of the Hippo Pathway. Increasing Rae1 levels leads to Rae1 deposition outside the nucleus including at the plasma membrane where activation of the pathway occurs. Rae1 is a conserved regulator of actin and microtubule networks *in vitro* [41–42, 66], and we showed that Rae1 promotes microtubule acetylation *in vivo*. Rae1 effects on microtubules could reflect a role in mitosis and spindle assembly [41–45, 47, 59–60, 66]. Alternatively, tubulin acetylation is implicated in activation of Hippo signaling in some contexts [94]. Mer interacts with acetylated microtubules; mutants disrupting this association promote YAP nuclear localization [67–69, 94]. Mer is crucial in recruiting Wts to the membrane to signal [64–65]. Collectively, these observations are consistent with a model that Rae1 could regulate tubulin acetylation and/or stability to correctly localize Mer to allow for Wts recruitment for proper Hippo signaling (Fig 10).

We observed that Rae1 increases the protein levels of Mer, Hpo and Wts. Rae1 regulation of Wts is conserved; Rae1 loss reduces Wts levels in *Drosophila* tissues and reduces pLats levels in mammalian cells, and other reports indicate Rae1 interactions with Mst2 in HEK-293T cells [95]. Previous reports showed that upstream component Fat promotes accumulation of Wts protein in *Drosophila* [96–97]. Future work will address if stabilization of Hpo and Wts occurred via upstream stabilization of Mer, akin to Fat regulation of Wts protein levels. Previous work addressing the instability of Sav protein showed Hpo/Mst association with Sav is stabilizing in both flies and mammalian systems [98–99]. Our observations are consistent with an alternate but not mutually exclusive model that Rae1 could promote stabilization of a complex of core pathway components by promoting their assembly following proper recruitment of Wts to the membrane or by direct association.

### A possible role for Rae1 in cancer and cancer therapeutics

Disrupting the tight link between proliferation and organ size can have serious consequences in normal development and in diseases such as cancer. Hippo Pathway dysregulation is associated with a broad spectrum of cancers, and mutations in upstream component Merlin are associated with the familial tumor syndrome Neurofibromatosis Type 2 (NF2) [2–19]. Our findings in both *Drosophila* and mammalian cells demonstrate that (1) high levels of Rae1 promote proliferation, (2) that Rae1 levels are controlled by Hippo signaling, and (3) that this increased proliferation due to high levels of Rae1 allows cells to evade the organ size checkpoint. We showed that Rae1 over-expression could promote proliferation of human cancer cells in culture, and our data suggests that Rae1 protein may accumulate upon loss of Hippo signaling in cancer cells. TCGA data indicates Rae1 amplification in a range of cancers [100–101] and Rae1 protein levels accumulate in gliomas [102], a tumor type where loss of *Mer* and Hippo Pathway function are frequently implicated [11].

Importantly, we showed that decreasing Rae1 dramatically compromised the survival of tissue with abrogated Hippo signaling. This means that maintaining sufficient Rae1 was crucial in the context of Hippo Pathway loss; when Rae1 levels did not reach a critical threshold, the tissue underwent massive catastrophe. Elucidating this phenomenon could have tremendous impact for cancer therapeutics. In recent years, evidence has emerged that cancer cells rely heavily on individual genes for survival (oncogene and non-oncogene “addiction”) [103–106] It has also been proposed that “second site mutations” that do not impair viability of wild-type tissue can disadvantage cancer cells with specific primary lesions, and that this “synthetic lethality” can be exploited therapeutically [107]. Given the role of Rae1 to promote cell proliferation and increase organ size, its conserved regulation by the Hippo pathway in both cultured insect and mammalian cells, and the synthetic tissue lethality phenomenon observed in Hippo-compromised tissue, we propose that Rae1 may represent a novel therapeutic target in cancers arising due to loss of Hippo Pathway tumor suppression (Fig 10).

## Materials and Methods

### Drosophila

Flies were raised on standard media at 25°C unless otherwise stated. Genotypes are detailed are detailed separately for larval and adult tissues appearing in image panels and tissue analyzed in Western blots. The coding region of Rae1 was cloned into pUAST. Genetic Services, Inc. performed vector injection of pUAST-Rae1 and isolated independent transgenic lines.

Adult wings of progeny were photographed, all at the same magnification. For quantitation, between 4 and 20 wings per genotype were traced using Adobe Photoshop CS5 or ImageJ, and wing areas were normalized to the average area in control. For *engal4* wings, we measured area posterior to vein L4. For *c5gal4*, total wing area is shown. Because of the effect on eye shape and size with both Hippo and Yki over-expression and Rae1 loss of function phenotypes, we found side-by-sides the best way to represent genetic interactions with Rae1 with respect to eye size. To rule out unintentional observer bias, experiments were scored blind with lab members evaluating eyes without knowledge of genotypes. We also indicate quantification of eye outlines traced and measured using Image J. The data shown in the figures are representative experiments that have been performed independently at least 3 times.

### Immunohistochemistry and western analysis

Larvae were dissected and stained using standard protocols and imaged on a Leica TSC-SP confocal. S2 cells were stained using standard protocols and imaged on Zeiss Axio Imager.Z1. Antibodies, anti-FlagM2 (1:500, Sigma), anti-pHH3 (1:1000, Upstate), anti-BrdU (1:500, BD Biosciences), anti-cyclin B (1:25, DSHB) anti-cyclin A (1:25, DSHB), Alexa-Fluor 488 and 555 goat anti-mouse (1:4000), Alexa-Fluor 555 goat anti-rabbit (1:4000), Molecular Probes/Invitrogen. Antibodies, anti-FlagM2 (1:2000, Sigma), anti-myc 9E10 (1:1000, mouse, Santa Cruz Biotechnology, SCB), anti-myc A14 (1:1000, rabbit, SCB), anti-cyclin B d-300 (1:500, SCB), anti-phosphoMst1/2 (1:1000, Cell Signaling), anti-Mst1/2 (1:1000, Cell Signaling), anti-Cdc2 (PSTAIR) (1:1000, SCB), anti-Rae1 (1:8000, Sigma), anti-HA (1:1000, Roche) anti-Tubulin (1:8000, Sigma) anti-Lats1 (1:500, Cell Signaling), anti-Yap (1:500, SCB), anti-pRXXS (1:1000, Cell Signaling), Alexa-Fluor goat anti-mouse 680 (1:20,000), Alexa-Fluor goat anti-rabbit 800 (1:20,000, Molecular Probes/Invitrogen), anti-rabbit IgG-conjugated HRP (1:4000, GE Healthcare) or anti-mouse IgG-conjugated HRP (1:4000 dilution; GE Healthcare). Westerns of cultured cell extracts were visualized with the Li-Cor Odyssey. Westerns of *Drosophila* tissue extracts were developed using Clarity Western ECL Substrate (Bio-Rad). Results from immunohistochemical staining and Western Analysis were reproduced in at least three independent trials.

### Genotypes of larvae and adults analyzed in all figures

*w; Rae1*^*ex28*^*/+* (left larva in Fig 2A)

*w; Rae1*^*ex28*^ (right larva in Fig 2A)

*w; actgal4/+* (2O; left eye in S4S Fig; black bars in Fig 2B, 2C; 2M and 2Q and S4A, S4R Fig; black tracings in Fig 2P and S4S’ Fig)

*w; UAS Rae1*^*IRV*^*/+; actgal4/+* (pink bars in Fig 2B and 2C, white bar in S4A Fig)

*w; UAS Rae1*^*IRN2*^*/+; actgal4/+* (hashed pink bars in Fig 2B and 2C)

*w; actgal4/UAS Rae1*^*IRN3*^ (striped pink bars in Fig 2B and 2C)

*UAS dcr2; nubgal4/+* (Figs 2D and 6C and S11L, S11P and S13I Figs; black bars in Fig 2F)

*UAS dcr2; nubgal4/UAS Rae1*^*IRV*^ (Fig 2E; pink bars in Fig 2F)

*w; nubgal4/+* (S4H Fig)

*w; nubgal4/+; Rae1*^*IRT*^*/+* (S4I Fig)

*w; nubgal4/+; Rae1*^*IRT*^ (S4J Fig)

*w; eygal4/+* (Fig 2G and Figs 3C and 7I, left eyes in 2R and S4V, S5C, S5O, S5Q, S7H, S8N and S8X Figs; solid bars in Fig 3E and 3K’, black bars in Fig 2J and S4L Fig, pink bars in S5E and S8R Figs, black tracing in Fig 2V’)

*w; eygal4/UAS Rae1*^*IRV*^ (Figs 2H and 3D and S5P Fig; pink bar in S4L Fig, hashed bars in Fig 3E)

*UAS dcr2/+; eygal4/UAS Rae1*^*IRV*^ (Fig 2I and 2J and S8D and S8I Fig; left eyes in S4N, S10A and S10B Figs; hashed and striped bars in S4L Fig, pink bars in S8G and S8M Fig)

*yweyFLP/+; FRT42D/FRT42D l(2) pW+* (left eye in Fig 2K; black tracing in Fig 2K’; black bar in S4M Fig)

*yweyFLP/+; FRT42D Rae1*^*ex28*^*/FRT42D l(2) pW+* (left eye in Fig 1K; pink tracing in Fig 1K’; pink bar in S4M Fig)

*w; GMRgal4/+* (left eye in Fig 2L and 2S; black tracing in Fig 2L’ and 2S’; black bar in Fig 1I and S4Q Fig)

*w; UAS Rae1*^*IRV*^*; GMRgal4/+* (right eye in Fig 2L; pink tracing in Fig 2L’; pink bar in S4Q Fig)

*UAS dcr2/+; eygal4/UAS Rae1*^*IRV*^*; UAS-Rae1*^*02*^/+ (Right eye in Fig 2N)

*UAS dcr2/+; engal4/+* (Figs 6I and 7A and S4B and S13M Figs)

*UAS dcr2/+; engal4/UAS Rae1*^*IRV*^ (Figs 4E and 7B and S4C, S4D, S4O and S10C Figs; pink tracing in Fig 4F–4G and 4L–4L’ and S4P, S10D and S10E Figs; pink bar in Fig 4L”)

*UAS dcr2/fzr*^*G0326*^*; engal4/UAS Rae1*^*IRV*^ (Fig 4L; black tracing in Fig 4L’; black bar in Fig 4L”)

*UAS dcr2/+; engal4/UAS Rae1*^*IRV*^*; UAS-Rae1*^*02*^/+ (S4P Fig)

*w; c5gal4/+* (Figs 6Q and 7E and S4E, S5J, S11A, S11E, S12G, S13A and S13P Figs; black bar in Fig 2J; black tracing in Fig 6T and 6V)

*w; Rae1*^*ex28*^*/+; c5gal4/+* (S5K Fig; pink bar in Fig 3F)

*w; UAS Rae1*^*IRV*^*/+; c5gal4/+* (S5L and S6F Figs; light pink bar in Fig 3F)

*w; Rae1*^*ex28*^*/UAS Rae1*^*IRV*^*; c5gal4/+* (S5M Fig; lightest pink bar in Fig 3F)

*w; dppgal4*, *UAS GFP/+* (S4K’ Fig; black bars in S2N and S4K Figs)

*w; UAS Rae1*^*IRV*^/+; *dppgal4*, *UAS GFP/+* (Pink bars in S2K Fig)

*w*^*1118*^ (Pink bars in S8H Fig)

*w; eygal4/UAS Rae1*^*IRN2*^ (S5D, S5F and S5R Fig)

*w; eygal4/UAS Rae1*^*IRV*^*; UAS p35/+* (Hashed pink bar in S5E Fig)

*yw hsFLP UAS GFP; Act>y+>gal4/+* (Pink line in S5A and S5B Fig)

*yw hsFLP UAS GFP/+; UAS Rae1*^*IRV*^*/Act>y+>gal4* (Fig 3A and 3A’ and S5H-S5I’ Fig, green line in S5A Fig)

*yw hsFLP UAS GFP/+; UAS Rae1*^*IRN2*^*/Act>y+>gal4* (Fig 3B and 3B’ and S5G and S5G’, S8C and S8C’ Figs)

*UAS dcr/fzr*^*G0326*^*; eygal4/UAS Rae1*^*IRV*^ (Right eye in Fig 4K; black tracing in Fig 4K’)

*UAS dcr2/+*; *engal4/UAS Rae1*^*IRV*^; *cycA*^*c05304*^/+ (Fig 4F, yellow bar in Fig 4H)

*UAS dcr2/+*; *engal4/UAS Rae1*^*IRV*^; *cycB*^*2*^/+ (Fig 4G, orange bar in Fig 4H)

*UAS dcr2/+*; *eygal4/UAS Rae1*^*IRV*^; *cycA*^*c05304*^/+ (S8E Fig, yellow bars in S8G Fig)

*UAS dcr2/+*; *eygal4/UAS Rae1*^*IRV*^; *cycB*^*2*^/+ (S8F Fig, orange bars in S8G Fig)

*w; cycA*^*c05304*^/+ (yellow bars in S8H Fig)

*w; cycB*^*2*^/+ (orange bars in S8H Fig)

*UAS dcr2/+*; *eygal4/UAS Rae1*^*IRV*^; *UAS cycE*/+ (S8J Fig, red bars in S8M Fig)

*UAS dcr2/+*; *eygal4/UAS Rae1*^*IRV*^/*UAS* *cycA* (S8K Fig, yellow bars in S8M Fig)

*UAS dcr2/+*; *eygal4/UAS Rae1*^*IRV*^/ *UAS cycB3* (S8L Fig, orange bars in S8M Fig)

*UAS dcr2/fzr*^*G0326*^*; engal4*, *UAS Rae1*^*IRV*^/+ (Fig 4L; black tracing in Fig 4L’; black bar in Fig 4L”)

*w; eygal4/+; UAS Rae1*^*02*^*/+* (right eye in Fig 2R and S8U Fig; hashed bars in Fig 3K’; pink tracing in Fig 2R’)

*w; eygal4/+; UAS Rae1*^*03*^*/+* (S5A and S7I Figs; pink bar in Fig 3J)

*yw hsFLP UAS GFP; Act>y+>gal4/+; UAS Rae1*^*02*^*/+* (green line in S5B Fig)

*yw hsFLP UAS GFP; Act>y+>gal4/+; UAS Rae1*^*03*^*/+* (S8S and S8S’ Fig)

*w; GMRgal4/ UAS Rae1*^*02*^ (right eye in Fig 2S; pink hashed bar in S4Q Fig; pink tracing in Fig 2S’)

*w; GMRgal4/ UAS Rae1*^*03*^ (pink striped bar in S4Q Fig)

*w; actgal4/UAS Rae1*^*02*^ (Fig 2P; pink bar in Fig 2M and 2Q and S4A and S4R Fig)

*w; actgal4/UAS Rae1*^*03*^ (Pink hashed bar S4A and S4R Fig)

*w; actgal4/UAS Rae1*^*GFP*^ (Fig 9H, right eye in S4S Fig; pink tracing in S4S’ Fig)

*w; actgal4/UAS yki*^*V5*^ (eye in S4U Fig)

*w; eygal4/+; UAS yki*^*V5*^*/+* (right eye in S4V Fig; yellow tracing in S4V’ Fig)

*w; eygal4/+; UAS cycE/+* (S8X Fig)

*w; eygal4/+; UAS cycE*^*IRT*^*/+* (S8Y Fig)

*w; GMR Hpo/+* (S9A–S9D Fig and S10B Fig; left eyes in Fig 5A and 5H and S9E, S9F, S9H and S9J Fig; black tracing in Fig 5A’ and S9H’ and S9J’ Fig, blue tracing in Fig 5H, blue bars in Fig 5H”, black bar in S10F Fig)

*w; GMR Hpo/Rae1*^*ex28*^ (right eye in Fig 5A and S9H and S10B Figs; pink tracing in Fig 5A’ and S9H’ Fig)

*w; GMR Sav*, *Wts/+* (left eye in Fig 5B; black tracing in Fig 5B’)

*w; Rae1*^*ex28*^*/+; GMR Sav*, *Wts/+* (right eye in Fig 5B; pink tracing in Fig 5B’)

*w; GMR Wts/+* (left eye in Fig 5C; black tracing in Fig 5C’)

*w; Rae1*^*ex28*^*/+; GMR Wts/+* (right eye in Fig 5C; pink tracing in Fig 5C’)

*w; GMR Hpo/GMRgal4* (left eye in Fig 5D; black tracing in Fig 5D’)

*w; GMR Hpo/GMRgal4; UAS Rae1*^*02*^*/+* (right eye in Fig 5D; pink tracing in Fig 5D’)

*w; UAS hpo/+; c5gal4/+* (Figs 5E and S9M; blue tracing in Fig 5F and S9N Fig; blue bar in Fig 5G)

*w; UAS hpo/+; c5gal4/UAS Rae1*^*GFP*^ (Fig 5F; pink bar in Fig 5G)

*fzr*^*G0326*^*/+; GMR Hpo/+* (right eye in Fig 5H; black tracing in Fig 5H’; black bar in Fig 5H”)

*fzr*^*G0418*^*/+* (blue bar in S8V Fig)

*fzr*^*G0418*^*/+; GMR Hpo/+* (blue bar in S10F Fig)

*w; GMR Hpo/+; wts*^*3-17*^*/+* (S9G Fig; right eyes in S9E and S9F Fig)

*w; UAS hpo/+; c5gal4/wts*^*X1*^ (S9N Fig)

*w; GMR Hpo/Df(2R)ED3923* (right eye in S9J Fig; pink tracing in S9J’ Fig)

*w; GMR Sav*, *Wts/GMRgal4* (left eye S9K Fig; black tracing in S9K’ Fig)

*w; UAS Rae1*^*IRV*^*/+; GMR Sav*, *Wts/GMRgal4* (right eye S9K Fig; pink tracing in S9K’ Fig)

*w; GMR Hpo/+; actgal4/+* (left eye in S9L Fig; black tracing in S9L’ Fig)

*w; GMR Hpo/+; actgal4/Rae1*^*GFP*^ (right eye in S9L Fig; pink tracing in S9L’ Fig)

*UAS dcr2/+; eygal4*, *UAS Rae1*^*IRV*^*/hpo*^*MGH1*^ (right eye in S10A Fig)

*UAS dcr2/mer*^*4*^*; eygal4*, *UAS Rae1*^*IRV*^*/+* (right eye in S10B Fig)

*UAS dcr2/+; engal4*, *UAS Rae1*^*IRV*^/*hpo*^*KS240*^ *(*S10F Fig)

*UAS dcr2/+; engal4*, *UAS Rae1*^*IRV*^/+; *wts*^*3-17*^*/+* (S10E Fig)

*UAS dcr2/+; engal4/+; UAS hpo*^*IRT*^*/+* (Fig 6J and S12C Fig; blue tracing in Fig 6K and 6L)

*UAS dcr2/+; engal4*, *UAS Rae1*^*IRV*^*; UAS hpo*^*IRT*^*/+* (Fig 6K and S12D Fig)

*UAS dcr2/+; engal4/Rae1*^*ex28*^*; UAS hpo*^*IRT*^*/+* (Fig 6L)

*UAS dcr2/+; nubgal4/+; UAS hpo*^*IRT*^*/+* (Fig 6D; blue tracing in Fig 6E and 6F)

*UAS dcr2/+; nubgal4/Rae1*^*ex28*^*; UAS hpo*^*IRT*^*/+* (Fig 6E)

*UAS dcr2/+; nubgal4/UAS Rae1*^*IRV*^*; UAS hpo*^*IRT*^*/+* (Fig 6F)

*ms1096gal4/+; UAS hpo*^*IRT*^*/+* (Fig 6G)

*ms1096gal4/+; Rae1*^*ex28*^*/+; UAS hpo*^*IRT*^*/+* (Fig 6H’ and 6H”)

*UAS dcr2/+; engal4/UAS Mer*^*IRN*^ (Fig 6M and S12A Fig; green tracing in Fig 6N and S12B Fig)

*UAS dcr2/+; engal4*, *UAS Rae1*^*IRV*^*/UAS Mer*^*IRN*^ (Fig 6N and S12B Fig)

*UAS dcr2/+; engal4/+; UAS ex*^*IRT*^*/+* (Fig 6O; purple tracing in Fig 6P)

*UAS dcr2/+; engal4*, *UAS Rae1*^*IRV*^*/*+*; UAS ex*^*IRT*^*/+* (Fig 6P)

*w; c5gal4/UAS wts*^*IRT*^ (Fig 6R and S12H Fig; purple tracing in Fig 6T and 6V, S12I and S12J Fig)

*w; UAS yki*^*IRN*^*/+; c5gal4/UAS wts*^*IRT*^ (Fig 6S and S12J Fig; yellow tracing in Fig 6T)

*w; Rae1*^*ex28*^*/+; c5gal4/UAS wts*^*IRT*^ (Fig 6U–6U” and S12I Fig; pink tracing in Fig 6V)

*UAS dcr2/+; engal4/+; UAS yki*^*V5*^*/+* (Fig 7 and S13N Fig; yellow tracing in S13O Fig)

*UAS dcr2/+; engal4/UAS Rae1*^*IRV*^*; UAS yki*^*V5*^ /+ (S13O Fig)

*w; c5gal4/UAS yki*^*FLAG*^ (S13Q Fig; yellow tracing in S13R Fig)

*w; Rae1*^*ex28*^*/+; c5gal4/UAS yki*^*FLAG*^ (S13R Fig)

*w; GMRgal4/UAS yki*^*V5*^ (Left eye in S13S Fig; black tracing in S13S’ Fig; orange bars in Fig 1I)

*w; UAS Rae1*^*IRV*^*/+; GMRgal4/UAS yki*^*V5*^ (Right eye in S13S Fig; pink tracing in S13S’ Fig)

*w; GMRgal4/UAS yki*^*S168A*.*GFP*^ (Left eye in S13T Fig; black tracing in S13T’ Fig)

*w; UAS Rae1*^*IRV*^*/+; GMRgal4/UAS yki*^*S168A*.*V5*^ (Right eye in S13T Fig; pink tracing in S13T’ Fig)

*w; UAS yki*^*S168A*.*GFP*^*/+; c5gal4/+* (Fig 7F)

*w; UAS yki*^*S168A*.*GFP*^*/Rae1*^*ex28*^*; c5gal4/+* (Fig 7G)

*w; UAS yki*^*S168A*.*GFP*^*/UAS Rae1*^*IRV*^*; c5gal4/+* (Fig 7H)

*w; UAS yki*^*S168A*.*GFP*^*/eygal4* (Fig 7J)

*w; UAS yki*^*S168A*.*GFP*^*/eygal4*, *Rae1*^*ex28*^ (Fig 7K)

*w; UAS yki*^*S168A*.*GFP*^*/eygal4*, *UAS Rae1*^*IRV*^ (Fig 7L)

*w; dppgal4/+* (Fig 7M)

*w; UAS yki*^*S168A*.*GFP*^*/+; dppgal4/+* (Figs 7N and 8D–8D”)

*w; UAS yki*^*S168A*.*GFP*^*/Rae1*^*ex28*^*; dppgal4/+* (Figs 7O and 8E–8E”)

*w; UAS yki*^*S168A*.*GFP*^*/UAS Rae1*^*IRV*^*; dppgal4/+* (Fig 7P)

*w; ptcgal4/+; UAS Rae1*^*GFP*^*/+* (S14C and S14C’ Fig)

*w; ptcgal4/UAS Hpo; UAS Rae1*^*GFP*^*/+*(S14D and S14D’ Fig)

*w; actgal4/hpo*^*MGH1*^*; UAS Rae1*^*GFP*^*/+* (Fig 9I)

*w; actgal4/Uba1*^*B2*^*; UAS Rae1*^*GFP*^*/+* (Fig 9J)

*w; c5gal4/Rae1*^*IRT*^ (S11B and S11F Fig)

*w; UAS Hpo*^*KD*^*/+; c5gal4/+* (S11C and S11G Fig)

*w; UAS Hpo*^*KD*^*/+; c5gal4/UAS Rae1*^*IRT*^ (S11D and S11H Fig)

*w; UAS Hpo*^*KD*^*/Rae1*^*ex28*^*; c5gal4/+* (S11I and S11I’ Fig)

*w; UAS Hpo*^*KD*^*/UAS Rae1*^*IRV*^*+; c5gal4/+* (S11J and S11J’ Fig)

*UAS dcr2/+; nubgal4/+; UAS Rae1*^*IRT*^*/+* (S11M and S11Q Fig)

*UAS dcr2/+; nubgal4/UAS Hpo*^*KD*^ (S11N and S11R Fig)

*UAS dcr2/+; nubgal4/UAS Hpo*^*KD*^*; UAS Rae1*^*IRT*^*/+* (S11O and S11S Fig)

*w; engal4/+; UAS hpo*^*IRT*^*/+* (S12E Fig; blue tracing in S12F Fig)

*w; engal4/UAS Yki*^*IRN1*^*; UAS hpo*^*IRT*^*/+* (S12E Fig; blue tracing in S12F Fig)

*UAS dcr2/+; engal4/+; UAS myc*^*WT*^*/+* (S12L Fig; black tracing in S12M Fig)

*UAS dcr2/+; engal4/UAS Rae1*^*IRV*^*; UAS myc*^*WT*^*/+* (S12M Fig)

*UAS dcr2/+; engal4/+; UAS p35/+* (S12N Fig; black tracing in S12O Fig)

*UAS dcr2/+; engal4/UAS Rae1*^*IRV*^*; UAS p35/+* (S12O Fig)

*yw hsFLP UAS GFP; Act>y+>gal4/UAS Rae1*^*IRV*^*; UAS hpo*^*IRT*^*/+* (S12K Fig)

*w; c5gal4/UAS Yki*^*V5*^ (S13B Fig; yellow tracing in S13C and S13D Fig)

*w; Rae1*^*ex28*^*/+; c5gal4/UAS Yki*^*V5*^ (S13C Fig)

*w; Rae1*^*IRV*^*/+; c5gal4/UAS Yki*^*V5*^ (S13D Fig)

*w; engal4/+* (S13E Fig)

*w; engal4/+; UAS Yki*^*V5*^ (S13F Fig)

*w; engal4/Rae1*^*ex28*^*; UAS Yki*^*V5*^ (S13G Fig)

*w; engal4/Rae1*^*IRV*^*; UAS Yki*^*V5*^ (S13H Fig)

*UAS dcr2/+; nubgal4/+; UAS Yki*^*V5*^ (S13J Fig)

*UAS dcr2/+; nubgal4/ Rae1*^*ex28*^*; UAS Yki*^*V5*^ (S13K Fig)

*UAS dcr2/+; nubgal4/Rae1*^*IRV*^*; UAS Yki*^*V5*^ (S13L Fig)

*w; ptcgal4/+; UAS wts*^*myc*^ (Fig 9K–9K”)

*w; ptcgal4/+; UAS wts*^*myc*^*/Rae1*^*GFP*^ (Fig 9L–9L”)

### Genotypes of larvae analyzed in western blots

*w; UAS Rae1*^*GFP*^*/c5gal4* (Lane 1 in Fig 1C)

*w; hpo*^*MGH1*^*/+; UAS Rae1*^*GFP*^*/c5gal4* (Lane 2 in Fig 1C)

*w; Uba1*^*B1*^*/+; UAS Rae1*^*GFP*^*/c5gal4* (Lane 3 in Fig 1C)

*w; dppgal4*, *UAS GFP /+* (Lane 1 in Fig 1E, lane 1 in S2C Fig)

*w; dppgal4*, *UAS GFP /UAS Rae1*^*GFP*^ (Lane 2 in Fig 1E, lane 2 in S2C Fig)

*w; UAS hpo/+; dppgal4*, *UAS GFP/UAS Rae1*^*GFP*^ (Lane 3 in Fig 1E)

*w; UAS hpo*^*KD*^*/+; dppgal4*, *UAS GFP/UAS Rae1*^*GFP*^ (Lane 4 in Fig 1E)

*w; ptcgal4/+; UAS Rae1*^*GFP*^*/+* (Lane 1 in Fig 1F, lane 1 in Fig 1K, lane 2 in Fig 8I, lane 2 in Fig 9M, lane 1 in S2B Fig, lane 1 in S2D Fig, lane 1 in S2F Fig)

*w; ptcgal4/hpo*^*MGH1*^*; UAS Rae1*^*GFP*^*/+* (Lane 2 in S2B Fig, lane 2 in S2D Fig)

*w; ptcgal4/+; UAS Rae1*^*GFP*^*/wts*^*X1*^ (Lane 3 in S2B Fig)

*w; ptcgal4/Uba1*^*B1*^*; UAS Rae1*^*GFP*^*/+* (Lane 3 in S2D Fig)

*w; Uba1*^*B1*^*/+; dppgal4*, *UAS GFP/UAS Rae1*^*GFP*^ (Lane 3 in S2C Fig)

*w; ptcgal4/UAS hpo; UAS Rae1*^*GFP*^*/+* (Lane 2 in Fig 1F, lane 2 in S2F Fig)

*w; ptcgal4/UAS hpo*^*KD*^*; UAS Rae1*^*GFP*^*/+* (Lane 3 in S2F Fig)

*w; ptcgal4/UAS hpo; UAS Rae1*^*GFP*^*/UAS wts*^*KD*^ (Lane 3 in Fig 1F)

*w; ptcgal4/UAS Yki*^*IRV*^*; UAS Rae1*^*GFP*^*/+* (Lane 2 in Fig 1K)

*w; engal4/+* (Lane 1 in Fig 4J)

*w; engal4/+; UAS Rae1*^*GFP*^*/+* (Lane 2 in Fig 4J)

*w; engal4/+; UAS Rae1*^*GFP*^ (Lane 3 in Fig 4J)

*ey(3*.*5)-FLP; Act>y+>gal4/+*,*UAS GFP/+; FRT82B* (Lane 1 in Fig 6A, lane 1 in Fig 6B, lane 1 in S8A Fig, lane 1 in S8B Fig)

*ey(3*.*5)-FLP; Act>y+>gal4/+*,*UAS GFP/+; FRT82B wts*^*X1*^ (Lane 2 in Fig 6A, lane 2 in S8A Fig, lane 2 in S8B Fig)

*ey(3*.*5)-FLP; Act>y+>gal4/+*,*UAS GFP/+; FRT82B wts*^*X1*^ (Lane 2 in Fig 6A)

*ey(3*.*5)-FLP; Act>y+>gal4/+*,*UAS GFP/UAS Rae1*^*IRV*^*; FRT82B wts*^*X1*^ (Lane 3 in Fig 6A)

*ey(3*.*5)-FLP; Act>y+>gal4/+*,*UAS GFP/Rae1*^*ex28*^*; FRT82B wts*^*X1*^ (Lane 4 in Fig 6A)

*ey(3*.*5)-FLP; Act>y+>gal4/+*,*UAS GFP/+; FRT82B sav*^*4*^ (Lane 2 in Fig 6B)

*ey(3*.*5)-FLP; Act>y+>gal4/+*,*UAS GFP/UAS Rae1*^*IRV*^*; FRT82B sav*^*4*^ (Lane 3 in Fig 6B)

*ey(3*.*5)-FLP; Act>y+>gal4/+*,*UAS GFP/Rae1*^*ex28*^*; FRT82B sav*^*4*^ (Lane 4 in Fig 6B)

*w; c5gal4/+* (Lane 1 in Fig 8A and 8B)

*w; c5gal4/UAS Yki*^*V5*^ (Lane 2 in Fig 8A)

*w; Rae1*^*ex28*^*/+; c5gal4/UAS Yki*^*V5*^ (Lane 3 in Fig 8A)

*w; UAS Rae1*^*IRV*^*/+; c5gal4/UAS Yki*^*V5*^ (Lane 4 in Fig 8A)

*w; c5gal4/UAS Yki*^*FLAG*^ (Lane 2 in Fig 8B)

*w; Rae1*^*ex28*^*/+; c5gal4/UAS Yki*^*FLAG*^ (Lane 3 in Fig 8B)

*w; UAS Rae1*^*IRV*^*/+; c5gal4/UAS Yki*^*FLAG*^ (Lane 4 in Fig 8B)

*w; ptcgal4/+* (Lane 1 in S13U Fig, Lane 1 in Fig 8I, Lane 1 in Fig 9F, Lane 1 in Fig 9M)

*w; ptcgal4/+; UAS Yki*^*V5*^*/+* (Lane 2 in S13U Fig)

*w; ptcgal4/ Rae1*^*ex28*^*; UAS Yki*^*V5*^*/+* (Lane 3 in S13U Fig)

*w; ptcgal4/UAS Rae1*^*IRV*^*; UAS Yki*^*V5*^*/+* (Lane 4 in S13U Fig)

*w; UAS yki*^*S168A*.*GFP*^*/ptcgal4; UAS Rae1*^*GFP*^*/+* (Lane 3 in Fig 8I)

*w; UAS yki*^*S168A*.*GFP*^*/ptcgal4* (Lane 4 in Fig 8I)

*w; ptcgal4/+; UAS myc wts/+* (Lane 1 in Fig 9B)

*w; ptcgal4/+; UAS myc wts/UAS Rae1*^*GFP*^ (Lane 2 in Fig 9B)

*w; UAS myc wts/c5gal4+* (Lane 1 in Fig 9C)

*w; Rae1*^*ex28*^*/+; UAS myc wts/c5gal4* (Lane 2 in Fig 9C)

*w; ptcgal4/UAS hpo* (Lane 2 in Fig 9F)

*w; ptcgal4/UAS hpo; UAS Rae1*^*GFP*^*/+* (Lane 3 in Fig 9F)

*w; nubgal4/+* (Lane 1 in Fig 9N)

*w; nubgal4/+; UAS Rae1*^*IRT*^*/+* (Lane 2 in Fig 9N)

*w; nubgal4/+; UAS Rae1*^*IRT*^ (Lane 3 in Fig 9N)

### Tissue culture

S2 cells cultured at 25°C were transfected with *Actin-Gal4*, *pIE1-4-myc-Hippo*, *pIE1-4-myc-Warts*, *pIE1-4-HA-Merlin* and *UAS-FLAG-His6-*

*Rae1*, *pAc5*.*1-His6-FLAGx3-Rae1*, *pAc5*.*1-Rae1-V5-His* using Cellfectin II (Invitrogen) or Effectene (Qiagen). HEK-293T, U87MG and HeLa cells were cultured in DMEM (Invitrogen) containing 10% FBS (Gemini) and 50 μg/mL penicillin/streptomycin (Gemini). Transfection with *pCMV5-FLAG-Mst1*, *pCMV2-FLAG2 Lats1*, *pCMV-FLAG Yap2 S127A*, *pCMV-FLAG-Yap2 5SA*, using Effectene (Qiagen) was performed according to the manufacturer's instructions.

### Mitotic index determination in S2 cells

The percentage of anti-phosphorylated histone H3 (Cell Signalling Technology)-positive S2 cells per total cells (mitotic index) was determined by scoring a total of at least 400 cells in each of four independent experiments.

### *Drosophila in vitro* Expression Cloning (DIVEC) screening

Bacterial stocks containing plasmids of *Drosophila* Gene Collection Releases 1 and 2 (representing more than 11,000 genes in the *Drosophila* genome) were grown as individual 1 ml cultures then pooled for isolation of plasmid DNA in pools of 12 and 16. Pools containing 12–16 plasmids were *in vitro* translated (IVT) using the Promega TNT combined *in vitro* transcription/translation kits and labeled with ^35^S-methione. Pool IVTs were incubated in the presence or absence of two unrelated kinases or a combination of recombinant Mst1 and Mst2 (Invitrogen) and run on a gel (*Drosophila In vitro* Expression Cloning) [48–50]. Bands representing individual clones in the pools were considered positive hits if showing a gel shift, smear, or other change after incubation with Hpo compared to the control incubation or incubation with two unrelated kinases.

### Cell lysis

S2 cells were harvested by centrifugation at 1000 g for 3 minutes. The cell pellet was washed once and resuspended in either 8M urea, 150 mM NaCl, 25 mM Tris, 1% NP40 and 1 mM EDTA or in 8M urea dissolved in PhosphoSafe Extraction Reagent (Novagen). In both cases, the lysis buffers were supplemented with 1 mM PMSF and protease inhibitor cocktail (Roche).

### Extract protocol

S2 cells were harvested by centrifugation at 400 g for 5 minutes. The cell pellet was resuspended in 300 μL of extract buffer (20 mM HEPES KOH pH 7.4, 50 mM KCl, 1.5 mM MgCl_2_, 1 mM EDTA, 1 mM EGTA, 1 mM DTT, 250 mM sucrose, 100 μg/mL cycloheximide, 1 mM PMSF, protease inhibitor cocktail (Roche), 1 mM NaF, 20 mM NaOPO_7_, 40 mM b-glycerophosphate and para-Nitrophenylphosphate, supplemented with either 20 μM MG132 or DMSO) and homogenized using 30 strokes of a Dounce homogenizer. Cell extracts were incubated at room temperature and stopped by the addition of 6x Laemmli sample buffer and boiling for 10 minutes

### CIP treatment

100 μg of protein was incubated with 200 units of calf-intestinal phosphatase (New England Biolabs) with NEB Buffer 3 supplemented with protease inhibitor cocktail (Roche) and 1 mM PMSF at room temperature for 10 minutes. Reactions were stopped by the addition of 6x Laemmli sample buffer and boiling for 10 minutes.

### RNAi

The DNA template was generated using the following primer sets (Table 1):

**Table 1:** Primer sets used to generate DNA templates for RNAi experiments.

Hippo RNAi F	TAATACGACTCACTATGGGAGTCCGCAGAAGCCACCACCATCG
Hippo RNAi R	TAATACGACTCACTATGGGAGCCAAGTTCGACTCCAGCTCCACC
Warts RNAi F	TAATACGACTCACTATGGGAGAAGGCGGCCACGGTG
Warts RNAi R	TAATACGACTCACTATGGGAGCTCCTTCTCCTTGGAGATCT
Rae1 RNAi F	TAATACGACTCACTATAGGGTGCTGGACGTTTGCTGGTCG
Rae1 RNAi R	TAATACGACTCACTATAGGCTTTTGGATTCCCCGGATTCA
Yki RNAi F	TAATACGACTCACTATAGGAGGACATGCTTTCGCCGATCA
Yki RNAi R	TAATACGACTCACTATAGGACTGTTCTCTGGGAAAGTGGG

dsRNA was generated using the T7 RiboMAX Express Large Scale RNA Production System (Promega), followed by DNAse digestion using RQ1 RNAse-free DNAse (Promega). 1x10^6^ cells were treated with 15 μg dsRNA for 48–72 hours and then transfected with appropriate plasmids (Fig 4C and 4M) or co-transfected with plasmids and dsRNA (S8F Fig).

### Quantitative RT-PCR (qPCR)

RNA from approximately 15 larvae of each genotype or 30 adult heads was extracted using TRIzol reagent (Invitrogen). For mammalian cells, RNA was extracted using the GeneJet RNA Purification kit (Thermo). In all cases, RNA was treated with RQ1 RNase-free DNase (Promega). 1 μg of RNA was reverse-transcribed using iScript cDNA synthesis kit (BioRad) and diluted 1:50 for each quantitative PCR reaction (QPCR SYBR Green ROX Mix (Fisher Scientific)).

The probes used were (Table 2):

**Table 2:** Probes for qPCR.

dmRae1 Left	AATCGACGAACCGAATGAAC
dmRae1 Right	CCCATGGTCTTCATCGACTT
dmGAPDH Left	GGCATTTCGCTGAACGAT
dmGAPDH Right	CAACAGTGATTCCCGACCA
dmEx Left	CAGCAGCAGCCGAAAACCT
dmEx Right	GGCGGGACGTCTCATCTTTC
hsCTGF Left	AAAAGTGCATCCGTACTCCCA
hsCTGF Right	CCGTCGGTACATACTCCACAG
hsRae1 Left	TGTGCTGACGTGATATACCCC
hsRae1 Right	GGGCAAAACCAGTAGGCTTGT
hsGAPDH Left	AAGGTGAAGGTCGGAGTCAAC
hsGAPDH Right	GGGGTCATTGATGGCAACAATA
hsYap Left	CAGCAGAACCGTTTCCCAGA
hsYap Right	GTCCACATTTGTCCCAGGAAT
hsTP53 Left	CTGCCCTCAACAAGATGTTTTG
hsTP53 Right	CTATCTGAGCAGCGCTCATGG
hsCDKN1A Left	ACTCTCAGGGTCGAAAACGG
hsCDKN1A Right	CCTCGCGCTTCCAGGACTG

### Statistics and reproducibility

Statistical analysis was performed using Excel. For all quantitative changes, T-test (with one-sided using equivalent variance) were conducted. For changes in categorical data (for example, incidence of black tissue or pHH3 cells), chi tests were conducted. All data presented represent typical findings from experiments performed a minimum of three times with appropriate controls.

### FACS

Approximately 20 third instar larval wing discs of each genotype were dissected in PBS and transferred to 0.5 mL of 10x Trypsin-EDTA solution (Sigma-Aldrich) and incubated for 3 hours at room temperature on a nutator. The cells were analyzed using FACScalibur and BD CellQuest Pro.

### Cell counts

24 hours after transfection, 2.5x10^4^ (for 293T, Rae1 over-expression) and 3.5x10^4^ (for HeLa, Rae1 over-expression) cells were added to each well of multiple12-well cell culture dishes. For the Rae1 knockdown experiment, 1.0x10^5^ cells (for 293T, Rae1 knockdown) were added to each well of multiple 6-well cell culture dishes.

Cells were incubated at 37°C until harvest. At approximately 24, 48, and 72 hours post-seeding, one dish was retrieved from incubation and the contents of each well were aspirated. Each well was washed twice in 1x PBS. The adherent cells were dissociated with 0.05% Trypsin/EDTA and subsequently counted using a Coulter Counter (Beckman) or using Cell Countess (Invitrogen).

### BrdU incorporation

Third instar larval eye discs were dissected in serum-free Schneider’s media and then incubated with 8 μg/mL BrdU for 30 minutes at room temperature. Discs were washed first in serum-free media then in PBS and fixed in 4% paraformaldehyde (diluted in PBS) for 30 minutes. They were permeabilized in PBS/0.1%Triton 100 (PBT) and incubated in 2N HCl solution (diluted in PBT) for 30 minutes. Cells were washed in PBT and incubated overnight in anti-BrdU (1:500, BD Biosciences) and standard protocols were followed for secondary staining. BrdU incorporation was imaged on a Zeiss AxioImager Z1 and AxioVision Release 4.8 and/or also on a Leica TSC-SP confocal.

### Direct kinase assays

Rae1 and YAP Peptides were synthesized by Genscript and incubated individually with commercially available Lats2 (Invitrogen), spotted onto P81 phosphocellulose cation exchange paper (Whatman). The P81 paper was washed at least five times in 0.5% orthophosphoric acid until counts were no longer detectable in the washes, rinsed with ethanol, and air-dried. The dried P81 papers were mixed with Ready Safe scintillation mix (Beckman) and counted in a Beckman liquid scintillation counter. Purified Rae1 (a kind gift from Y Ren and the Blobel lab) or MBP (Sigma) were diluted in kinase assay buffer (50 mM Tris pH8, 10 mM MgCl_2_, 1 mM DTT) incubated in the presence of 100 μM cold ATP, 10 μCi [γ-32P]ATP and recombinant Lats2 (Invitrogen). The reaction mixtures were incubated for 20 minutes at 30°C, terminated with SDS sample buffer, and subjected to SDS-PAGE and autoradiography.

## Supporting Information

S1 Fig Rae1 was a strong hit from the DIVEC screen. (A) Schematic summarizing the DIVEC screen to identify novel substrates of the Hippo and Warts kinases. The *Drosophila* Gene Collection releases 1 and 2 were combined into pools of 12–16 clones. Pools were *in vitro* translated (IVT), labeling all clones with ^35^S methionine. Pool IVTs were incubated with two unrelated kinases or with recombinant Hippo (Mst1 and Mst2 recombinant protein purchased from Invitrogen). Positive hits were those which showed a shift (*), smearing, or alteration/loss of full length signal compared to the load lane in the presence of Hippo but not the unrelated kinases. Addition of Hippo protein activates the pathway to regulate downstream targets in the reticulocyte lysate [19], so the screen is designed to identify downstream targets of the core cassette, possibly both Hippo and Warts/Lats substrates. (B) Gel from DIVEC screen showing Rae1 (arrow) as a positive hit in the pool. Almost all of Rae1 shifted to a slower migrating form (*) in the presence of added Hippo (right-most lane, asterisk), but does not shift in control lane (left-most lane, arrow), or in the presence of two unrelated kinases (middle lanes). We therefore classified Rae1 as a strong hit targeted directly by recombinant Mst1/2, by activated reticulocyte Lats1/2, or by another enzyme in the reticulocyte lysate activated by Mst1/2 and/or Lats1/2. An advantage of this screening approach is that it allowed us to identify both direct kinase targets and targets further downstream that are modified by enzymes in the lysate in an Mst/Lats-dependent manner. (C) A pool showing no positive hits. All bands show similar migration and levels in the Hippo lane (right-most lane) as in the load control lane (left-most lane).
(PDF)

S2 Fig The Hippo Pathway negatively regulates Rae1 downstream of Warts. (A) The predominant slower migrating Rae1 band (right lane, *) in MG132-treated S2 extracts from Rae1 and Hpo co-transfected cells (the band that predominates in Fig 1B) is decreased (arrow) when incubated in the presence of phosphatase (left lane). Mild debris is seen in left lane. (B) Rae1-GFP protein levels are sensitive to the gene dosage of *hpo* (reduced by introducing one copy of the *hpo*^*MGH1*^ allele, lane 2) and *wts* (reduced by introducing one copy of the *wts*^*X1*^ allele, lane 3), compared to control (*+/+*, lane 1) in *Drosophila* salivary glands. (C) Rae1-GFP protein levels are increased when the ubiquitin pathway is impaired at the level of the Ubiquitin Activating Enzyme E1. Reducing the gene dosage of E1 (reduced by introducing one copy of the *Uba1*^*B1*^ allele, lane 3) increases Rae1 levels compared to control (+/+, lane 2) in *Drosophila* salivary glands. (D) Rae1-GFP protein levels are sensitive to the gene dosage of *hpo* (reduced by introducing one copy of the *hpo*^*MGH1*^ allele, lane 2) and ubiqiuitin pathway impairment (reduced by introducing one copy of the *Uba1*^*B1*^ allele, lane 3) compared to control (+/+, lane 2) in *Drosophila* salivary glands. (E) Co-transfecting S2 cells with a c-terminally tagged *Rae1* and *hpo* (lane 2) causes loss of Rae1 protein levels compared to control-transfected cells (lane 1). RNAi to *hpo* (lane 3) or *wts* (lane 4) stabilizes Rae1 in the presence of co-transfected *hpo* compared to cells treated with control RNAi (second lane). (F) Over-expressing a wild-type (lane 2) but not a kinase-dead (lane 3) Hpo transgene in the context of Rae1-GFP over-expression in salivary glands shows a reduction in Rae1-GFP protein compared to controls (lane 1). (G) Over-expression of both *Mst1* and *Lats1* in HeLa cells showed loss of endogenous Rae1 protein levels compared to control-transfected cells. (H) Transfection of increasing *Mst1* levels showed a dose-dependent loss of endogenous Rae1. (I) HEK293T cells expressing human *myc-Rae1* were co-transfected with *Mst1* showing a dose-dependent decrease in Rae1 protein levels (lanes 1–3). Concomitant over-expression of baculovirus caspase inhibitor p35 to block apoptosis did not block Rae1 reduction in the Mst1-over-expressing cells (lanes 4–5). In B-I, relative levels of Rae1 (normalized by GFP in blot C, and Tubulin in all other blots) and Mst1 in H (normalized by Tubulin) are indicated.
(PDF)

S3 Fig Investigating Rae1 regulation by Warts/Lats and Yki/YAP. (A) The region surrounding the Lats1 consensus site (red box) in Rae1 is strongly conserved across species. Cells co-transfected with *Mst1* and/or La*t*s1 and *myc-Rae1* showed decreased Myc-Rae1 levels in the whole cell lysate (WCL) and also immunoprecipitated Rae1 (Myc-IP) as expected. Immunoprecipitated Rae1 was recognized by an anti-phospho-RXXS antibody (Lats1 consensus site), and the percentage of Rae1 phosphorylated at the Lats consensus motif increased with increased pathway activation. Relative levels of phosphorylated Rae1 are indicated. Quantification of anti-phospho-RXXS antibody (Lats1 consensus site) recognition of Myc-Rae1 immunoprecipitated from whole cell lysates of cells co-transfected with *Mst1* and/or *Lats1* are indicated as relative levels below the blot and in the graph below (normalized to the amount of immunoprecipitated total Rae1). (B) Peptides were generated using 11 amino acids (underlined in black in A) for *Drosophila* Rae1 (dmRae1) and human Rae1 (hsRae1) with alanine mutants that abolished the Wts consensus site (RXXA) Similar control and alanine mutant peptides were generated for YAP (hsYAP). A peptide kinase assay using Lats2 showed robust phosphorylation of wild-type YAP peptide but not of the S127A mutant peptide or of any of the Rae1 (wild-type or alanine mutant) peptides. (C) Kinase assays using Lats2, Myelin Basic Protein (MBP), and full length, purified baculovirus Rae1 [54] (a gift from Y. Ren and the Blobel lab). Coomassie gel shows levels of MBP and Rae1 protein used, and phosphorimage shows no significant phosphorylation of Rae1 in the presence of Lats2 compared to the MBP positive control. (D-F) Co-transfecting S2 cells with *yki* RNAi causes no change in Rae1 localization (E) compared to control-transfected cells (D). *Rae1* RNAi causes a reduction of the membrane-bound pool of FLAG-Rae1 (F). Scale bars in D-F indicate 5 μm. (G) Rae1 protein levels over three independent experiments were quantified upon Yki or Rae1 RNAi. (H) Over-expression of constitutively active Yap constructs (Yap^S127A^ or Yap^S5A^) where some or all of the Lats consensus sites have been mutated did not increase human Myc-Rae1 protein levels compared to control-transfected cells. Relative levels of Rae1 (normalized to Tubulin) are indicated in H.
(PDF)

S4 Fig Rae1 modulation in proliferating cells modulates organ size. (A) qPCR indicates the reduction in relative mRNA levels of *Rae1* upon RNAi using *actgal4* and *Rae1*^*IRV*^, and the increase in Rae1 levels upon *Rae1* over-expression using *actgal4* and *Rae1*^*02*^ and *Rae1*^*03*^ transgenes. Low-level constitutive *Rae1* RNAi with *actgal4* led to approximately 50% reduction, whereas *Rae1*^*02*^ over-expression increased levels to almost eight-fold over endogenous and *Rae1*^*03*^ to two-fold over endogenous. (B-D) RNAi to *Rae1* in the posterior compartment of the wing (C-D) causes a Rae1 dose-dependent reduction in wing size compared to controls (*en>dcr*, B). (E-G) RNAi to *Rae1* in the whole wing (F-G) causes a Rae1 dose-dependent decrease in wing size compared to controls (*c5gal4/+*, E). (H) Control *nubgal4* wing. (I-J) Decreasing *Rae1* by RNAi (*nub>Rae1*^*IRT*^) reduces wing size. (K-K’) RNAi to *Rae1* in a stripe in the developing wing using *dppgal4* and *Rae1*^*IRV*^ (*dpp>dcr*, *Rae1*^*IRV*^) reduces the area of the wing between the L3 and L4 wing veins (region highlighted in K’) in both males and females and also reduced overall wing area compared to controls. N = 13, 11 (females), N = 14, 14 (males). (L) Quantification of eyes shown in Fig 2G–2J. RNAi to Rae1 reduces eye size; increased reduction is seen in the presence of *dcr* and at higher temperatures. N = 20, 14, 14, 10. (M) Quantification of eyes shown in Fig 2K. Eyes containing primarily homozygous *Rae1*^*ex28*^ tissue are smaller than control eyes. Entire eye size was measured; in some cases, the eyes were composed primarily of unflipped tissue with little to no *Rae1*^*ex28*^ tissue. N = 16, 8 (males), N = 15, 14 (females). (N) The reduced eye size of *Rae1* RNAi (*ey>dcr*, *Rae1*^*IRV*^, left eye in N) is suppressed by over-expressing *Rae1* using transgenes *Rae1*^*02*^, *Rae1*^*03*^, *Rae1*^*GFP*^ (*ey>dcr*, *Rae1*^*IRV*^, *Rae1*^*02*^, right eye in N; male eyes are shown). (O-P) The reduced wing size due to *Rae1* RNAi in the posterior wing (*en>dcr*, *Rae1*^*IRV*^, O and red tracing in P) is suppressed by *Rae1* over-expression using transgenes *Rae1*^*02*^, *Rae1*^*03*^, *Rae1*^*GFP*^ (*en>dcr*, *Rae1*^*IRV*^, *Rae1*^*02*^). (Q) Quantification of eyes undergoing Rae1 RNAi or Rae1 over-expression using GMR gal4 (as shown in Fig 2L and 2S). N = 16,17,19,14 (females). (R) Constitutive *Rae1* over-expression (*Act>Rae1*^*02*^, *Act>Rae1*^*03*^) increases body length and wing area compared to *actgal4/+* controls. N = 18, 17, 12 (females) and N = 17, 17, 5 (males). (S) Constitutive *Rae1* over-expression (*act>Rae1*^*GFP*^, right eye in S, pink tracing in S’) increases eye size relative to controls (*actgal4*, left eye in S, black tracing in S’). (T) Over-expressing *Rae1* in clones, in actively dividing cells in the early eye (shown for *ey>Rae1*^*03*^) resulted in a normal pattern of ELAV staining. Scale bar indicates 35 μM. (U) Constitutive *Yki* over-expression (*act>Yki*^*V5*^) decreases eye size relative to controls. (V-V’) Yki over-expression in the proliferating eye cells decreases eye size (*ey>Yki*^*V5*^, right in V, orange tracing in V’) compared to control eyes (*eygal4*/+, left in V, black tracing in V’). * indicates statistically significant change from controls, p<0.05.
(PDF)

S5 Fig Loss of Rae1 results in proliferation phenotypes but not in aberrant cell size, differentiation, or survival. (A) Reduced organ size was not due to reduced cell size. Mosaic analysis using flip-out methods (*hsFLP; Act>y+>gal4*, *UAS GFP/UAS Rae1*^*IRV*^) positively labeled RNAi clones with GFP. Mosaic wing discs were dissected, dissociated, and subjected to FACS analysis. Forward scatter of GFP-positive *Rae1* RNAi cells (*Rae1*^*IRV*^, green, normalized cell size was 1.03, indicated on the top right-hand corner box) showed no statistically different cell size compared to GFP-negative wild-type clones (pink, normalized cell size was 1.00) as obvious by the similar FSC-height peak (indicated on the X-axis). The difference in counts (y-axis) reflects the greater number of wild-type cells, not cell size. (B) Similar experiments using mosaic analysis and flip-out methods (*UAS hsFLP; Act>y+>gal4*, *UAS GFP/UAS Rae1*^*02*^) positively labeled over-expression clones with GFP. Forward scatter of GFP-positive *Rae1* over-expressing cells (*Rae1*^*02*^, green, relative cell size of 0.96) showed no statistically different cell size compared to GFP-negative wild-type clones (pink, relative cell size of 1.00) as obvious by the similar FSC-height peak (indicated on the X-axis). (C-D) TUNEL assays indicating cell death showed no obvious change between *eygal4/+* controls (C), *ey>Rae1*^*IRN2*^ (D), and *ey>Rae1*^*IRV*^ in third instar larval eye discs. (E) Over-expressing the caspase inhibitor p35 did not suppress the reduced eye size upon *Rae1* RNAi. N = 11, 16. Parallel experiments in the presence of *dcr2* gave similar results. (F) RNAi of *Rae1* in actively dividing cells in the early eye (shown for *ey>Rae1*^*IRN2*^) resulted in a normal pattern of ELAV staining, indicating photoreceptor differentiation progresses normally. (G-H’) Clones undergoing constitutive *Rae1* RNAi (green in G, H) showed decreased BrdU incorporation (red in G, H panels in G’, H’), shown here in two examples of entire discs from two different RNAi lines. Reduced BrdU incorporation was most evident in clones in the SMW, possibly because division synchronizes in the SMW. (I-I’) *Rae1* RNAi clones (green in I) (using *Rae1*^*IRV*^ and *Rae1*^*IRN2*^ (shown for *Rae1*^*IRV*^), showed no obvious decrease in pHH3 staining (red I, panel I’). (J-M) Wing disc images (of discs quantified in 3F) undergoing *Rae1* reduction through heterozygosity at the *Rae1* locus (*c5gal4*, *Rae1*^*ex28*^*/+*, K), *Rae1* RNAi in the whole wing disc (*c5>Rae1*^*IRV*^, L) or both (*c5>Rae1*^*IRV*^, *Rae1*^*ex28*^*/+*, M) show an increase in pHH3 positive cells relative to control wing discs (*c5gal4/+*, J). Scale bars in I-L indicate 100 μm. (N) Graph showing the percentage of pHH3-positive S2 cells per total cells when treated with Rae1 RNAi (blue hashed bar) or control RNAi (blue bar). Rae1 RNAi increases the mitotic index (percentage of pHH3-positive cells). (O-P) BrdU incorporation (white) in (O) control *eygal4/+* disc and (P) an eye disc undergoing *Rae1* RNAi in actively dividing cells (*ey>Rae1*^*IRV*^). (Q-R) CycB staining (white) appeared similar in the region posterior the SMW in an eygal4/+ control disc (Q) as in a disc undergoing *Rae1* RNAi in (R, *ey>Rae1*^*IRV*^). Importantly, the high pHH3 staining posterior to the SMW but lack of BrdU incorporation suggests the pHH3 positive cells are not actively cycling. pHH3 staining but lack of CycB staining suggests these cells are in a stage of the cell cycle after CycB degradation but before the mitotic phosphorylation is removed from Histone H3, such as anaphase or early telophase. Parallel experiments in the presence of *dcr2* gave similar results. Scale bars in C-D, G indicate 50μm, in F, Q-R indicate 35μm, in H, O-P indicate 25 μm, and in J-M indicate 100 μm.
(PDF)

S6 Fig Knockdown of Rae1 results in nuclear phenotypes. (A-B) Examples of S2 cells undergoing control RNAi stained for pHH3 (red), tubulin (green) and DAPI (blue). (C-E) S2 cells undergoing Rae1 RNAi stained for pHH3 (red), tubulin (green) and DAPI (blue). The pHH3 and tubulin staining (shown in both merge and individual channels) show significant abnormalities compared to control cells shown in A-B. Scale bars indicate 5 μm.
(PDF)

S7 Fig Rae1 regulates proliferation in osteosarcoma cell lines and *Drosophila* tissues. (A-B) Rae1 loss in the U2OS osteosarcoma cell line by siRNA transfection (B) reduces cell proliferation compared to controls (A). (C-D) Rae1 loss in the SJSA osteosarcoma cell line by siRNA transfection (D) reduces cell proliferation compared to controls (C). (E-G) Proliferative arrest induced by Rae1 knockdown in HeLa (E), U2OS (F) and SJSA (G) cells is not mediated by increased p21 (*CDKN1A*) (mRNA levels were normalized to GAPDH). (H) BrdU incorporation in a control *eygal4/+* disc. (I) BrdU incorporation in a disc over-expressing Rae1 in actively dividing cells in the early eye, *ey>Rae1*^*02*^. Staining anterior to the MF increased, and the width of the SMW increased. Scale bars in A-D indicate 75 μm, in H-I indicate 25 μm.
(PDF)

S8 Fig Rae1 regulates and genetically interacts with the mitotic cyclins. (A) *wts*^*X1*^ MARCM eye discs (lane 2) show increased cyclin A and E protein levels compared to control MARCM discs (lane 1) consistent with previous reports. (B) *wts*^*X1*^ MARCM eye discs (lane 2) show increased cycB protein levels compared to control MARCM discs (lane 1). Relative levels of cyclins (normalized to Tubulin) are indicated in A and B. (C-C’) Clones undergoing *Rae1* RNAi (green in C, white tracing in C’) showed reduced cycA staining (red) in all regions of the eye disc with actively proliferating cells. Quantifying staining intensity suggests a decrease of more than 35% in the Rae1 RNAi clone in the SMW. (D-F) Mutation in *cycA* (E) or cycB (F) enhances the eye size reduction of RNAi to Rae1 in the early eye (*ey>dcr*, *Rae1*^*IRV*^, D). (G) Quantification of eyes in D-F. N = 16, 13, 12 (males), N = 17, 14, 16 (females). (H) Mutation in *cycA* or in *cycB* does not dominantly reduce wing size. N = 13, 14, 12 (males), N = 12, 13, 16 (females). (I-L) Over-expressing cycE (J), cycA (K), or cycB3 (L) partially suppresses the eye size reduction of RNAi to Rae1 in the early eye (*ey>dcr*, *Rae1*^*IRV*^, I). (M) Quantification of eyes from I-L except male eyes over-expressing cycA. Increased lethality of cycA over-expression in this context resulted in few males for quantification. N = 14, 13, 13 (males), N = 12, 12,1 8, 15 (females). (N-Q) Increased expression of cycE (O), cycA (P), or cycB3 (Q) in the early eye does not increase eye size compared to controls (N). (R) Quantification of eyes in N-Q. Increased lethality of cycA over-expression in this context resulted in few males for quantification. CycA over-expression on its own reduced eye size. N = 12, 17, 12 (males), N = 16, 17, 13, 17 (females). (S-S’) Rae1 over-expressing clones (green in S, tracing in S’) show increased cycA staining (red). Arrow in D’ indicates more intense cycA staining. Quantifying staining intensity suggests cycA staining increases more than 50% in the SMW. Arrowheads in S-S’ indicate the MF. (T-U) Over-expressing *Rae1* in actively dividing cells (U) increased the intensity of cyclin B staining anterior to the furrow and in the SMW compared to control *eygal4/+* discs (T). (V) Mutation in *fzr* does not dominantly alter wing size (shown for allele *fzr*^*G0418*^). N = 13, 21. (X-Y) Decreasing *cycE* expression in actively dividing cells (*ey>cycE*^*IRT*^, Y) decreased eye size compared to control (X). Scale bars in T-U indicate 35 μm.*indicates statistically significant difference p<0.001.
(PDF)

S9 Fig Hippo over-expression organ size phenotypes are sensitive to downstream targets. (A-C) Transgenic over-expression of *hpo* in differentiating eye cells (*GMR Hpo*) is responsive to temperature. Increasing the temperature increases expression levels of Hpo, and therefore increases the severity of the phenotype. *GMR Hpo* eyes were small and rough at 25°C (A), and became smaller, rougher, and showed increased appearance of black tissue at 28°C (B) and 30°C (C). (D) The presence of black tissue was quantified over a range of temperatures. N = 46, 60, 156, 38, 142 (males), N = 48, 58, 130, 60, 222 (females). (E-F) Removing one copy of *wts* by introducing the mutant allele *wts*^*3-17*^ suppressed the *GMR Hpo* eye size and black tissue phenotypes at 28°C (E) and 30°C (F). (G) Quantification of the black tissue showed modification of the *GMR Hpo* phenotype by adjusting *wts* gene dosage. This indicates that the black tissue phenotype can be used to reflect genetic modification of Hpo over-expression phenotypes. N = 38, 30, 142, 42 (males), N = 60, 34, 222, 44 (females). (H) Removing one copy of Rae1 by introducing the deletion allele *Rae1*^*ex28*^ (right eye, pink tracing in H’) further reduced *GMR Hpo* (left eye, black tracing in H’) eye size at 30°C (highlighted by tracings in H’) and enhanced the appearance of black tissue. (I) Quantification of black tissue appearance highlights the dominant enhancement by the *Rae1*^*ex28*^ allele at 28°C. (J-J’) Removing one copy of *Rae1* using the deficiency *Df(2R)ED3923* (right eye in J, pink tracing in J’), enhanced the phenotype of *GMR Hpo* (left eye in J, black tracing in J’) in terms of increasing eye roughness and further reducing eye size. (K-K’) Rae1 RNAi in differentiating eye cells using *GMRgal4* and *Rae1*^*IRV*^ (*GMR>Rae1*^*IRV*^) (right in in K, pink tracing in K’) enhanced the phenotype of over-expressing *Sav* and *Wts* in differentiating eye cells (*GMR Sav*, *Wts*) (left eye in K, black tracing in K’). (L-L’) Constitutively over-expressing *Rae1* (*act>Rae1*^*GFP*^, right in L, pink tracing in L’) suppressed the phenotype of *GMR Hpo* (left in L, black tracing in L’). (M-N) Transgenic over-expression of *hpo* in the wing (*c5>hpo*, blue tracing in M) causes a small wing with vein defects. Removing one copy of *wts* by introducing the mutant allele *wts*^*3-17*^ suppressed the Hippo over-expression wing size and vein specification phenotypes (N). * indicates statistically significant difference p<0.05.
(PDF)

S10 Fig Hippo over-expression organ size phenotypes are sensitive to Rae1. (A-B) Mutation in *hpo* (right eye in A) and *Mer* (right eye in B) dominantly suppressed the reduced eye size and eye roughness of RNAi to Rae1 in the early eye (left eyes in A, B). (C) Control *en>dcr*, *Rae1*^*IRV*^ wing (and tracing in in D-E,). (D-E) Mutations in *hpo* (E) and *wts* (E) dominantly suppressed the reduced wing size of RNAi to Rae1 in the posterior wing, highlighted by overlay of tracing of the wing in (C) (pink). (F) Quantification of eye size indicating significant suppression of the reduced eye size of *GMR Hpo* by mutation in *Cdh1/fzr* (*fzr*^*G0418*^ allele shown) as seen in Fig 5H and 5H’. N = 29, 6. * indicates statistically significant difference p<0.05.
(PDF)

S11 Fig Tissue overgrowing due to impaired Hippo kinase activity is sensitive to Rae1 levels. (A) Control wing (*c5gal4/+*). (B) RNAi to *Rae1* (*c5>Rae1*^*IRT*^) causes a moderate reduction in wing size. (C) Over-expression of a kinase-dead Hpo transgene (*c5>hpo*^*KD*^) moderately increases wing size relative to controls (A). (D) This moderate overgrowth is suppressed by RNAi to *Rae1*. (*c5>hpo*^*KD*^, *Rae1*^*IRT*^). (E) Control wing (*c5gal4/+*). (F) RNAi to *Rae1* (*c5>Rae1*^*IRT*^) causes a more dramatic reduction in wing size at 27°C. (G) Over-expression of a kinase-dead Hpo transgene (*c5>hpo*^*KD*^) promotes wing overgrowth relative to controls (E). (H) RNAi to Rae1 suppresses the dramatic overgrowth and also impairs the survival of overgrowing tissue caused by loss of Hippo activity (*c5>hpo*^*KD*^, *Rae1*^*IRT*^). (I-I’) Reducing Rae1 levels by introducing the *Rae1*^*ex28*^ allele caused a low penetrance of tissue collapse in the background of hippo signaling impairment (*c5>hpo*^*KD*^, *Rae1*^*ex28*^*/+*, I’). (J-J’) The *Rae1*^*IRV*^ shows less dramatic phenotypes than the *Rae1*^*IRT*^ transgene. Reducing Rae1 levels by RNAi caused a moderate penetrance of tissue collapse (*c5>hpo*^*KD*^, *Rae1*^*IRV*^, J’). (L) Control wing (*nub>dcr*). (M) RNAi to *Rae1* (*nub>dcr*, *Rae1*^*IRT*^) causes a reduction in wing size. (N) Over-expression of a kinase-dead Hpo transgene (*nub>dcr*, *hpo*^*KD*^) increases wing size relative to controls (L). (O) This overgrowth is suppressed by RNAi to *Rae1* (*nub>dcr*, *hpo*^*KD*^, *Rae1*^*IRT*^). (P) Control wing (*nub>dcr*) at 27°C. (Q) RNAi to *Rae1* (*nub>dcr*, *Rae1*^*IRT*^) causes a more dramatic reduction in wing size at 27°C. (R) Over-expression of a kinase-dead Hpo (*nub>dcr*, *hpo*^*KD*^) increases wing size relative to controls at 27°C (R). (S) RNAi to *Rae1* suppresses this overgrowth and promotes shriveling and blistering of wings (*nub>dcr*, *hpo*^*KD*^, *Rae1*^*IRT*^).
(PDF)

S12 Fig Reducing Rae1 suppresses overgrowth and impairs tissue survival upon reduction in Hippo signaling, but not upon over-expressing the myc oncogene or the caspase inhibitor p35. (A-B) Typically, RNAi to *Mer* (A, *en>dcr*, *Mer*^*IRN*^) leads to less overgrowth than RNAi to the other tumor suppressor components *ex*, *hpo*, and *wts*. In cases where we saw less overgrowth, concurrently reducing Rae1 (B, *en>dcr*, *Mer*^*IRN*^, *Rae1*^*IRV*^) suppressed overgrowth and often led to blistering and other mild phenotypes of tissue loss. This is consistent with the requirement for Rae1 changing depending on the extent of overgrowth. (C-D) Overgrowth due to loss of Hippo Pathway tumor components in the wing is often so dramatic that wings no longer lie flat; mounted wings thus wrongly appear smaller once they are flattened to be photographed. These images of flies with their wings still attached highlight how extensive the overgrowth is upon *hpo* RNAi (C, *en>dcr*, *hpo*^*IRT*^) and how effectively reducing *Rae1* suppressed this overgrowth (D, *en>dcr*, *hpo*^*IRT*^, *Rae1*^*IRV*^). (E-F) Reducing *yki* levels did not cause the same tissue lethality we saw with *Rae1*; *yki* RNAi (F, *en>hpo*^*IRT*^, *yki*^*IRN*^) suppressed the overgrowth but did not impair the survival of tissue overgrowing due to loss of *hpo* (E and blue tracing in F, *en>hpo*^*IRT*^). (G) Control *c5gal4/+* wing. (H) RNAi to *wts* (*c5>wts*^*IRT*^) caused wing overgrowth. (I) Removing one copy of Rae1 (*c5>wts*^*IRT*^*; Rae1*^*ex28*^*/+*) caused tissue loss. (J) RNAi to *yki* (*c5>wts*^*IRT*^*; yki*^*IRN*^) suppressed overgrowth but did not cause tissue loss. (K) Creating random clones undergoing concurrent RNAi to both *Rae1* and *hpo* causes patches of dying tissue throughout the fly. Image is of a dissected pharate adult showing black spots in the eye, and other large swaths of black tissue elsewhere. (L-O) The tissue lethality phenotype appears to be specific and did not occur for other overgrowth phenotypes we tested, shown here for over-expressing the *myc* oncogene and the caspase inhibitor *p35*. (L, black tracing in M) Control wing over-expressing the myc oncogene (*en>dcr*, *myc*^*WT*^). (M) Rae1 RNAi suppressed the overgrowth but did not cause tissue lethality in myc over-expressing wings (*en>dcr*, *myc*^*WT*^, *Rae1*^*IRV*^). (N, black tracing in O) Control wing over-expressing the caspase inhibitor p35 (*en>dcr*, *p35*). (O) Rae1 RNAi suppressed the overgrowth but did not cause tissue lethality in p35 over-expressing wings (*en>dcr*, *p35*, *Rae1*^*IRV*^).
(PDF)

S13 Fig Reducing Rae1 suppresses overgrowth upon over-expressing the Yki oncogene. (A) Control *c5gal4/+* wing at 27°C. (B) Yki over-expression (*c5>Yki*^*V5*^) caused wing overgrowth at 27°C. (C-D) Removing one copy of Rae1 by introducing *Rae1*^*ex28*^ (*c5>Yki*^*V5*^*; Rae1*^*ex28*^*/+*, C) or RNAi to *Rae1* (*c5>Yki*^*V5*^*; Rae1*^*IRV*^*/+*, D) at 27°C subtly but reproducibly increased overgrowth. (E) Control *engal4/+* wing at 27°C. (F) Yki over-expression (*en>Yki*^*V5*^) caused mild wing overgrowth in the posterior compartment at 27°C. (G) Removing one copy of Rae1 by introducing *Rae1*^*ex28*^ (*en>Yki*^*V5*^*; Rae1*^*ex28*^*/+*) increased wing overgrowth caused by Yki over-expression. (H) RNAi to *Rae1* (*en>Yki*^*V5*^*; Rae1*^*IRV*^*/+*) at 27°C suppressed overgrowth. (I) Control *nub>dcr* wing at 27°C. (J) Yki over-expression (*nub>dcr*, *Yki*^*V5*^) caused mild wing overgrowth in wing at 27°C. (K) Removing one copy of Rae1 by introducing *Rae1*^*ex28*^ (*nub>dcr*, *Yki*^*V5*^*; Rae1*^*ex28*^*/+*) increased wing overgrowth caused by Yki over-expression. (L) RNAi to *Rae1* (*nub>dcr*, *Yki*^*V5*^*; Rae1*^*IRV*^*/+*) at 27°C dramatically suppressed overgrowth. (M) Control wing (*en>dcr*). (N) Over-expressing Yki leads to wing overgrowth (N, and yellow overlay in O). (O) Reducing *Rae1* levels slightly by removing one copy suppressed tissue overgrowth but did not cause tissue collapse. (P) Control wing (*c5gal4/+*). (Q) Over-expressing a different Yki transgene leads to wing overgrowth (E, and yellow overlay in F). (R) Reducing *Rae1* levels slightly by removing one copy did not cause tissue collapse. Overgrowth in this context is quite dramatic such that wings no longer lie flat; mounted wings thus wrongly appear smaller once they are flattened to be photographed. (S-S’) Over-expressing Yki in the differentiating cells of the adult eye causes a larger eye (left in S, black tracing in S’). Reducing *Rae1* levels by concurrent *Rae1* RNAi (*GMR>Yki*^*V5*^, *Rae1*^*IRV*^, right in S, pink tracing in S’) further increases eye size. (T-T’) Over-expressing a constitutively active Yki transgene in the differentiating cells of the adult eye causes a larger eye (left in T, black tracing in T’). Reducing *Rae1* levels by concurrent *Rae1* RNAi (*GMR>Yki*^*S168A*^, *Rae1*^*IRV*^, right in T, pink tracing in T’) further increases eye size. (U) Reducing *Rae1* levels slightly by removing one copy (lane 3) or by RNAi (lane 4) increases Yki-V5 levels in larval salivary glands (lane 2). Rae1 reduction also reduced the proportion of a slower migrating species. Relative distribution of both bands of Yki are indicated.
(PDF)

S14 Fig Hippo signaling negatively regulates pools of Rae1 at the cell membrane and in the cytoplasm. (A-A”) Transfecting FLAG-Rae1 into S2 cells shows Rae1 association with the cell periphery, cytoplasm, and nucleus. (B-B”) Co-transfecting *FLAG-Rae1* with *myc-Hpo* into S2 cells reduced Rae1 levels, particularly at the cell periphery and cytoplasm. All images (A-B) were taken with identical exposure times and settings. Scale bars in A-B indicate 5 μm. (C-C”) Over-expressing a Rae1-GFP transgene in the salivary glands shows strong Rae1-GFP localization to the nuclear periphery but also localization to the cell membrane and cytoplasm. (D-D”) Co-over-expressing the Rae1-GFP with Hpo reduces the levels of Rae1 in salivary glands and specifically causes a reduction in the Rae1-GFP pools associated with the cell membrane and cytoplasm. There is also a reduction of Rae1 associated with the nuclear periphery and the pool of Rae1 in the nucleus appears to be associated with chromatin. All images (C-D) were taken with identical exposure times and settings. Scale bars in C-D indicate 20 μm.
(PDF)

S1 Table Genetic interactions between Hippo Pathway components and Rae1 in the eye. (DOCX)

S2 Table Summary of comparisons and differences between roles and phenotypes of Rae1 and Yorkie. Table summarizes key similarities and differences of over-expressing or reducing Rae1 and Yorkie in a variety of contexts, highlighting the different mechanisms of feedback regulation exerted upon the Hippo pathway.
(DOCX)
