# Supplementary figures and images for: The Hippo Pathway Targets Rae1 to Regulate Mitosis and Organ Size and to Feed Back to Regulate Upstream Components Merlin, Hippo, and Warts
Source: PLoS Genet. 2016 Aug 5;12(8):e1006198. doi: 10.1371/journal.pgen.1006198 (PMC4975479; doi:10.1371/journal.pgen.1006198)

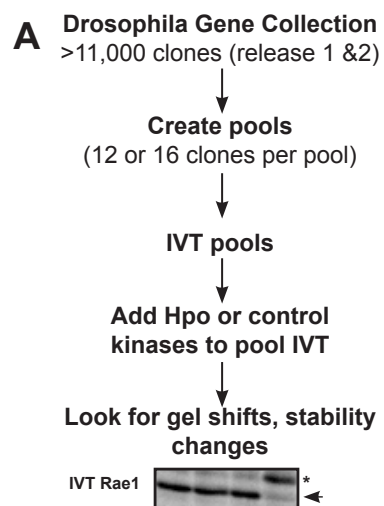

**B**

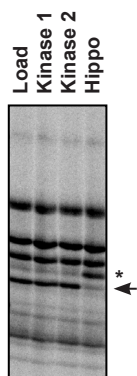

**C**

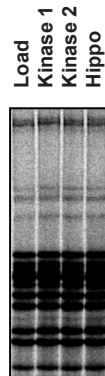

Supplement: S1 Fig — (A) Schematic summarizing the DIVEC screen to identify novel substrates of the Hippo and Warts kinases. The Drosophila Gene Collection releases 1 and 2 were combined into pools of 12–16 clones. Pools were in vitro translated (IVT), labeling all clones with 35S methionine. Pool IVTs were incubated with two unrelated kinases or with recombinant Hippo (Mst1 and Mst2 recombinant protein purchased from Invitrogen). Positive hits were those which showed a shift (*), smearing, or alteration/loss of full length signal compared to the load lane in the presence of Hippo but not the unrelated kinases. Addition of Hippo protein activates the pathway to regulate downstream targets in the reticulocyte lysate [19], so the screen is designed to identify downstream targets of the core cassette, possibly both Hippo and Warts/Lats substrates. (B) Gel from DIVEC screen showing Rae1 (arrow) as a positive hit in the pool. Almost all of Rae1 shifted to a slower migrating form (*) in the presence of added Hippo (right-most lane, asterisk), but does not shift in control lane (left-most lane, arrow), or in the presence of two unrelated kinases (middle lanes). We therefore classified Rae1 as a strong hit targeted directly by recombinant Mst1/2, by activated reticulocyte Lats1/2, or by another enzyme in the reticulocyte lysate activated by Mst1/2 and/or Lats1/2. An advantage of this screening approach is that it allowed us to identify both direct kinase targets and targets further downstream that are modified by enzymes in the lysate in an Mst/Lats-dependent manner. (C) A pool showing no positive hits. All bands show similar migration and levels in the Hippo lane (right-most lane) as in the load control lane (left-most lane). (PDF) [file pgen.1006198.s001.pdf]

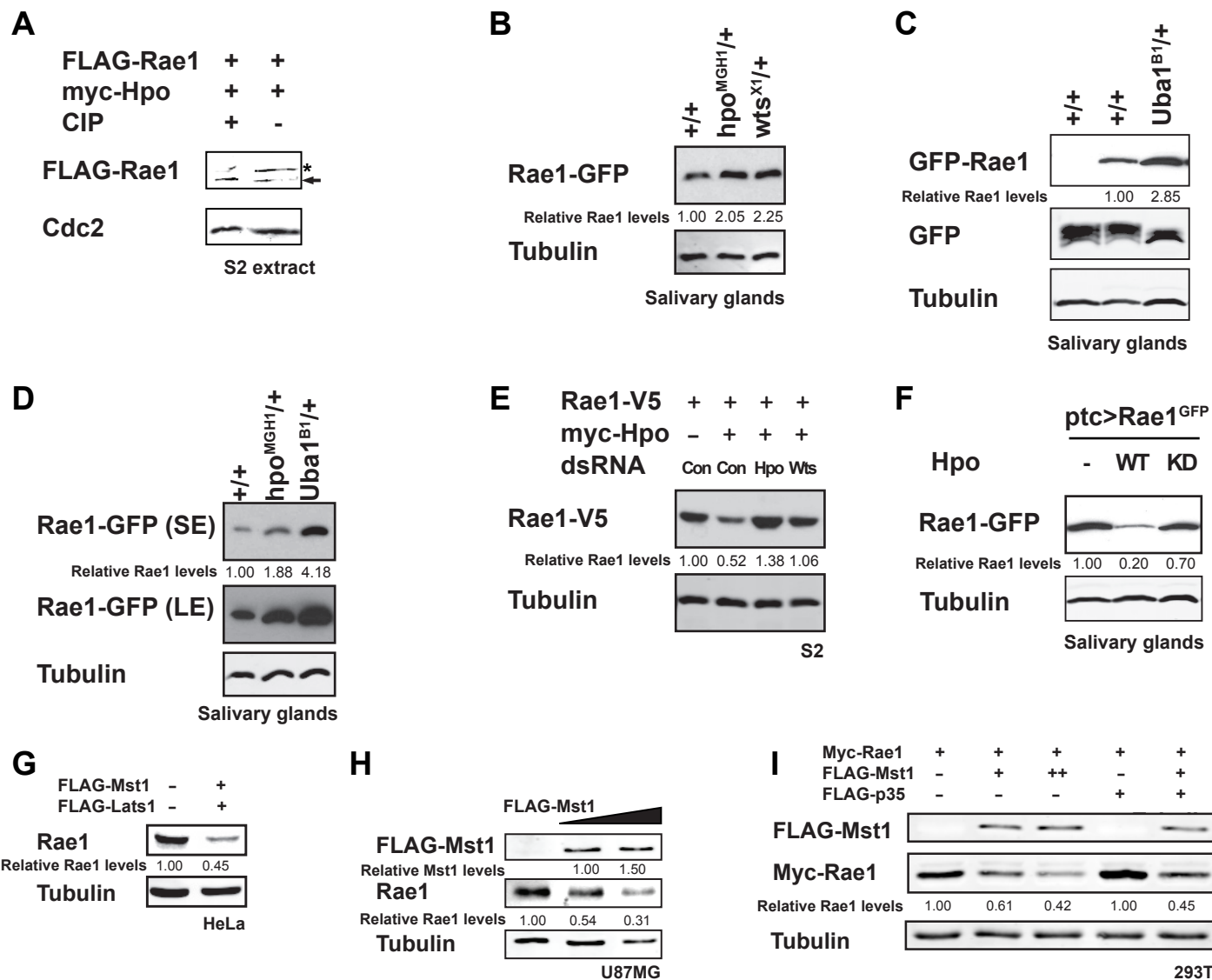

Supplement: S2 Fig — (A) The predominant slower migrating Rae1 band (right lane, *) in MG132-treated S2 extracts from Rae1 and Hpo co-transfected cells (the band that predominates in Fig 1B) is decreased (arrow) when incubated in the presence of phosphatase (left lane). Mild debris is seen in left lane. (B) Rae1-GFP protein levels are sensitive to the gene dosage of hpo (reduced by introducing one copy of the hpoMGH1 allele, lane 2) and wts (reduced by introducing one copy of the wtsX1 allele, lane 3), compared to control (+/+, lane 1) in Drosophila salivary glands. (C) Rae1-GFP protein levels are increased when the ubiquitin pathway is impaired at the level of the Ubiquitin Activating Enzyme E1. Reducing the gene dosage of E1 (reduced by introducing one copy of the Uba1B1 allele, lane 3) increases Rae1 levels compared to control (+/+, lane 2) in Drosophila salivary glands. (D) Rae1-GFP protein levels are sensitive to the gene dosage of hpo (reduced by introducing one copy of the hpoMGH1 allele, lane 2) and ubiqiuitin pathway impairment (reduced by introducing one copy of the Uba1B1 allele, lane 3) compared to control (+/+, lane 2) in Drosophila salivary glands. (E) Co-transfecting S2 cells with a c-terminally tagged Rae1 and hpo (lane 2) causes loss of Rae1 protein levels compared to control-transfected cells (lane 1). RNAi to hpo (lane 3) or wts (lane 4) stabilizes Rae1 in the presence of co-transfected hpo compared to cells treated with control RNAi (second lane). (F) Over-expressing a wild-type (lane 2) but not a kinase-dead (lane 3) Hpo transgene in the context of Rae1-GFP over-expression in salivary glands shows a reduction in Rae1-GFP protein compared to controls (lane 1). (G) Over-expression of both Mst1 and Lats1 in HeLa cells showed loss of endogenous Rae1 protein levels compared to control-transfected cells. (H) Transfection of increasing Mst1 levels showed a dose-dependent loss of endogenous Rae1. (I) HEK293T cells expressing human myc-Rae1 were co-transfected with Mst1 show [file pgen.1006198.s002.pdf]

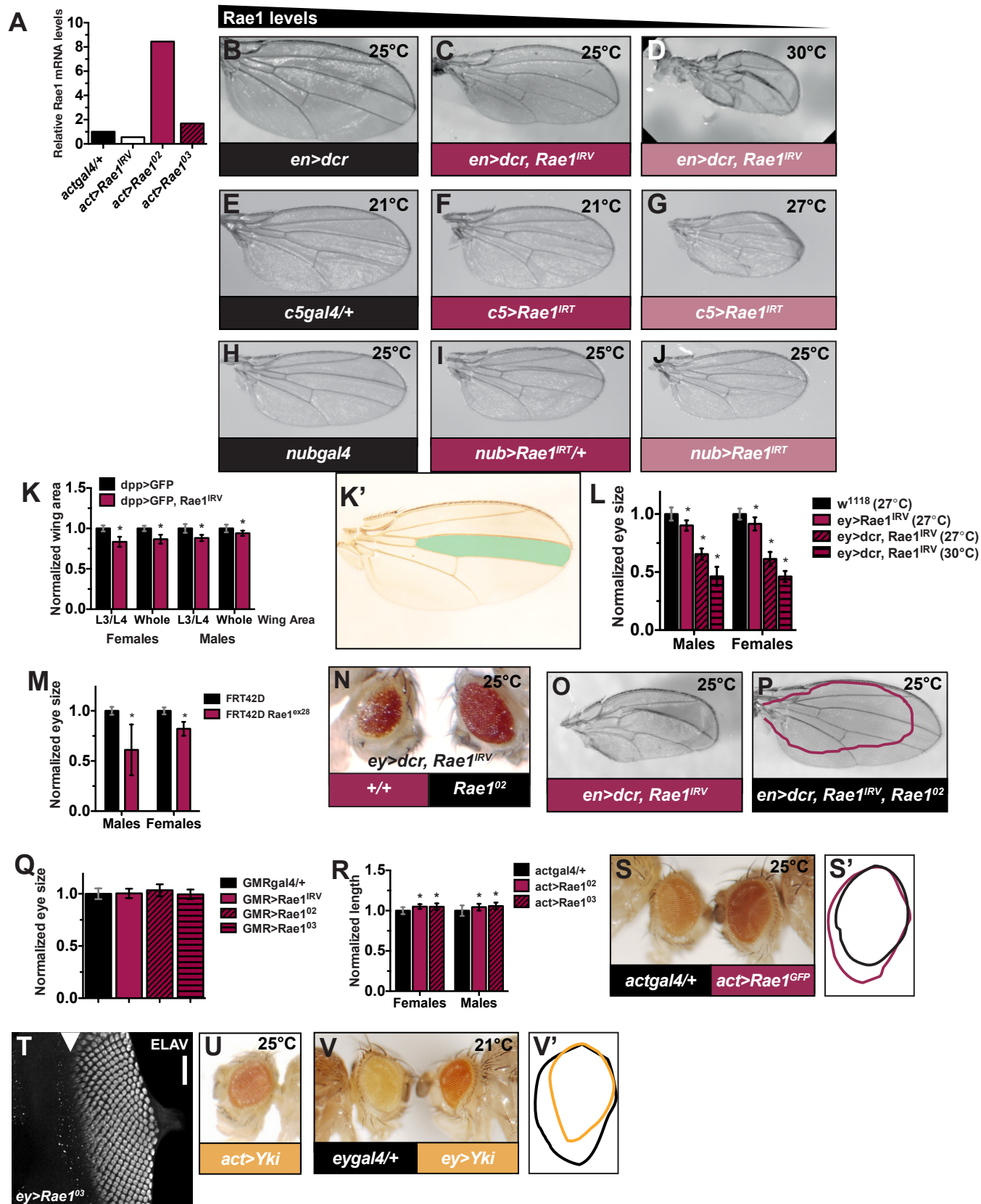

Supplement: S4 Fig — (A) qPCR indicates the reduction in relative mRNA levels of Rae1 upon RNAi using actgal4 and Rae1IRV, and the increase in Rae1 levels upon Rae1 over-expression using actgal4 and Rae102 and Rae103 transgenes. Low-level constitutive Rae1 RNAi with actgal4 led to approximately 50% reduction, whereas Rae102 over-expression increased levels to almost eight-fold over endogenous and Rae103 to two-fold over endogenous. (B-D) RNAi to Rae1 in the posterior compartment of the wing (C-D) causes a Rae1 dose-dependent reduction in wing size compared to controls (en>dcr, B). (E-G) RNAi to Rae1 in the whole wing (F-G) causes a Rae1 dose-dependent decrease in wing size compared to controls (c5gal4/+, E). (H) Control nubgal4 wing. (I-J) Decreasing Rae1 by RNAi (nub>Rae1IRT) reduces wing size. (K-K’) RNAi to Rae1 in a stripe in the developing wing using dppgal4 and Rae1IRV (dpp>dcr, Rae1IRV) reduces the area of the wing between the L3 and L4 wing veins (region highlighted in K’) in both males and females and also reduced overall wing area compared to controls. N = 13, 11 (females), N = 14, 14 (males). (L) Quantification of eyes shown in Fig 2G–2J. RNAi to Rae1 reduces eye size; increased reduction is seen in the presence of dcr and at higher temperatures. N = 20, 14, 14, 10. (M) Quantification of eyes shown in Fig 2K. Eyes containing primarily homozygous Rae1ex28 tissue are smaller than control eyes. Entire eye size was measured; in some cases, the eyes were composed primarily of unflipped tissue with little to no Rae1ex28 tissue. N = 16, 8 (males), N = 15, 14 (females). (N) The reduced eye size of Rae1 RNAi (ey>dcr, Rae1IRV, left eye in N) is suppressed by over-expressing Rae1 using transgenes Rae102, Rae103, Rae1GFP (ey>dcr, Rae1IRV, Rae102, right eye in N; male eyes are shown). (O-P) The reduced wing size due to Rae1 RNAi in the posterior wing (en>dcr, Rae1IRV, O and red tracing in P) is suppressed by Rae1 over-expression using transgenes Rae102, Rae103, Rae1GFP (en>dcr, Rae1IRV, R [file pgen.1006198.s004.pdf]

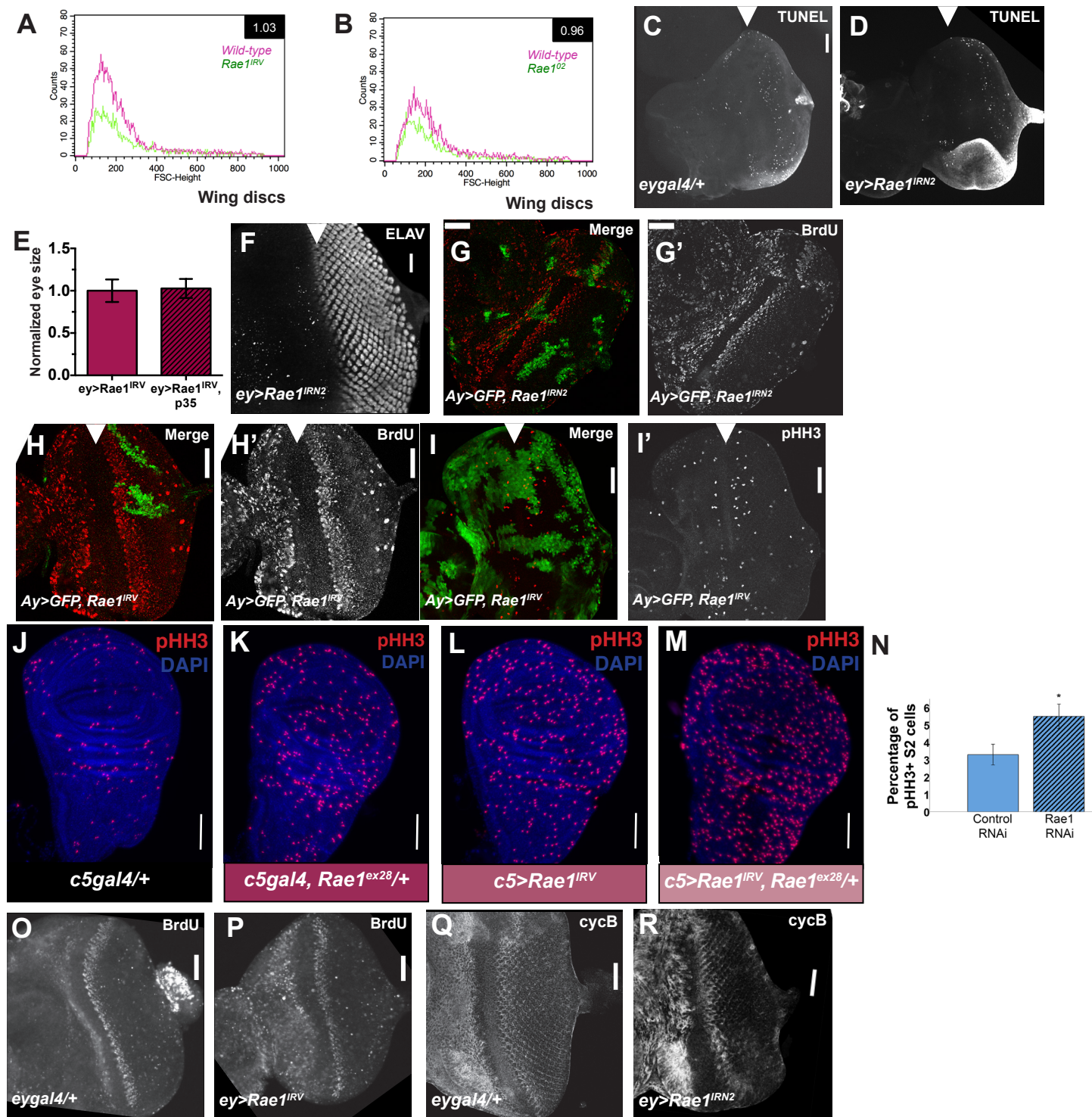

Supplement: S5 Fig — (A) Reduced organ size was not due to reduced cell size. Mosaic analysis using flip-out methods (hsFLP; Act>y+>gal4, UAS GFP/UAS Rae1IRV) positively labeled RNAi clones with GFP. Mosaic wing discs were dissected, dissociated, and subjected to FACS analysis. Forward scatter of GFP-positive Rae1 RNAi cells (Rae1IRV, green, normalized cell size was 1.03, indicated on the top right-hand corner box) showed no statistically different cell size compared to GFP-negative wild-type clones (pink, normalized cell size was 1.00) as obvious by the similar FSC-height peak (indicated on the X-axis). The difference in counts (y-axis) reflects the greater number of wild-type cells, not cell size. (B) Similar experiments using mosaic analysis and flip-out methods (UAS hsFLP; Act>y+>gal4, UAS GFP/UAS Rae102) positively labeled over-expression clones with GFP. Forward scatter of GFP-positive Rae1 over-expressing cells (Rae102, green, relative cell size of 0.96) showed no statistically different cell size compared to GFP-negative wild-type clones (pink, relative cell size of 1.00) as obvious by the similar FSC-height peak (indicated on the X-axis). (C-D) TUNEL assays indicating cell death showed no obvious change between eygal4/+ controls (C), ey>Rae1IRN2 (D), and ey>Rae1IRV in third instar larval eye discs. (E) Over-expressing the caspase inhibitor p35 did not suppress the reduced eye size upon Rae1 RNAi. N = 11, 16. Parallel experiments in the presence of dcr2 gave similar results. (F) RNAi of Rae1 in actively dividing cells in the early eye (shown for ey>Rae1IRN2) resulted in a normal pattern of ELAV staining, indicating photoreceptor differentiation progresses normally. (G-H’) Clones undergoing constitutive Rae1 RNAi (green in G, H) showed decreased BrdU incorporation (red in G, H panels in G’, H’), shown here in two examples of entire discs from two different RNAi lines. Reduced BrdU incorporation was most evident in clones in the SMW, possibly because division synchronizes in the S [file pgen.1006198.s005.pdf]

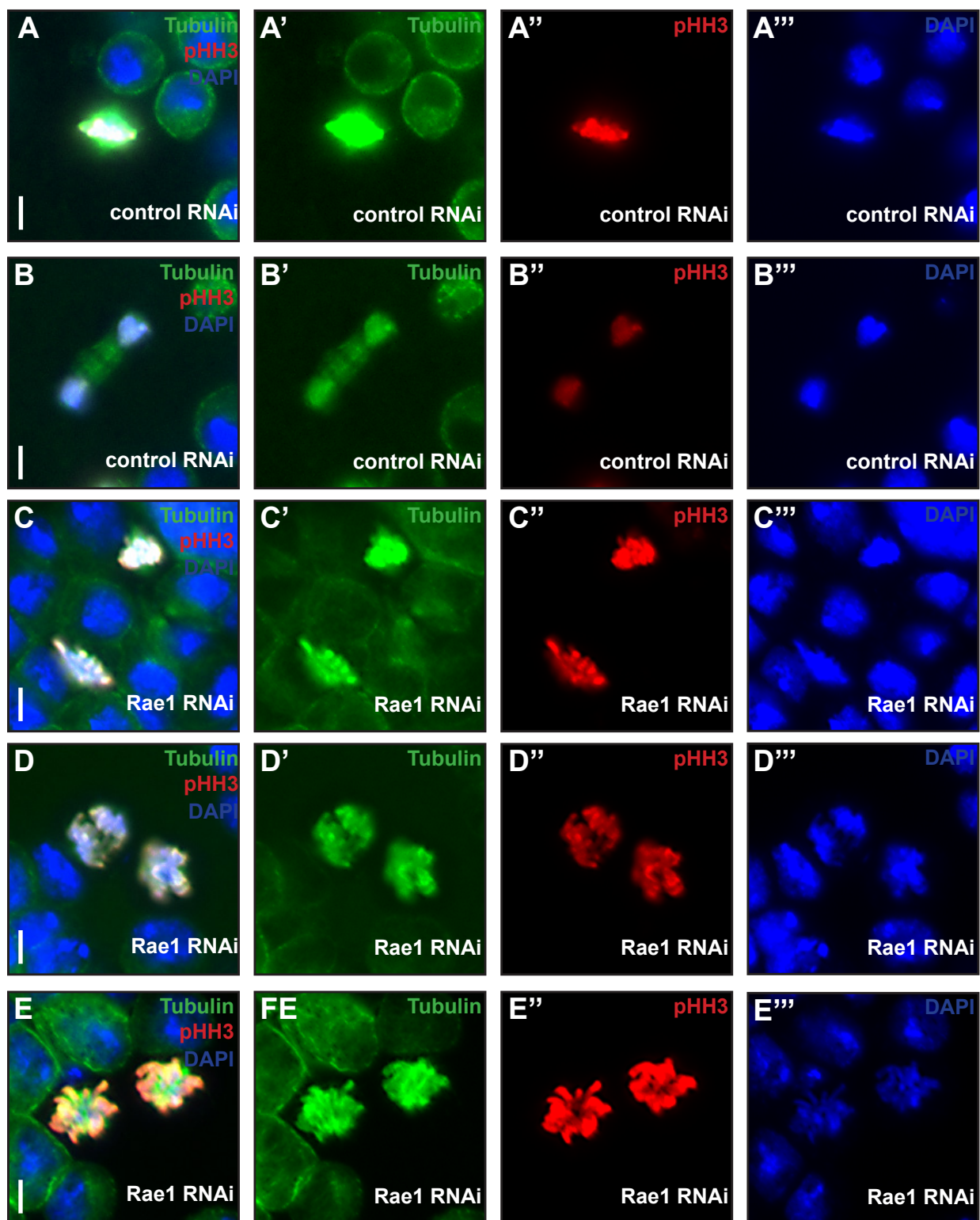

Supplement: S6 Fig — (A-B) Examples of S2 cells undergoing control RNAi stained for pHH3 (red), tubulin (green) and DAPI (blue). (C-E) S2 cells undergoing Rae1 RNAi stained for pHH3 (red), tubulin (green) and DAPI (blue). The pHH3 and tubulin staining (shown in both merge and individual channels) show significant abnormalities compared to control cells shown in A-B. Scale bars indicate 5 μm. (PDF) [file pgen.1006198.s006.pdf]

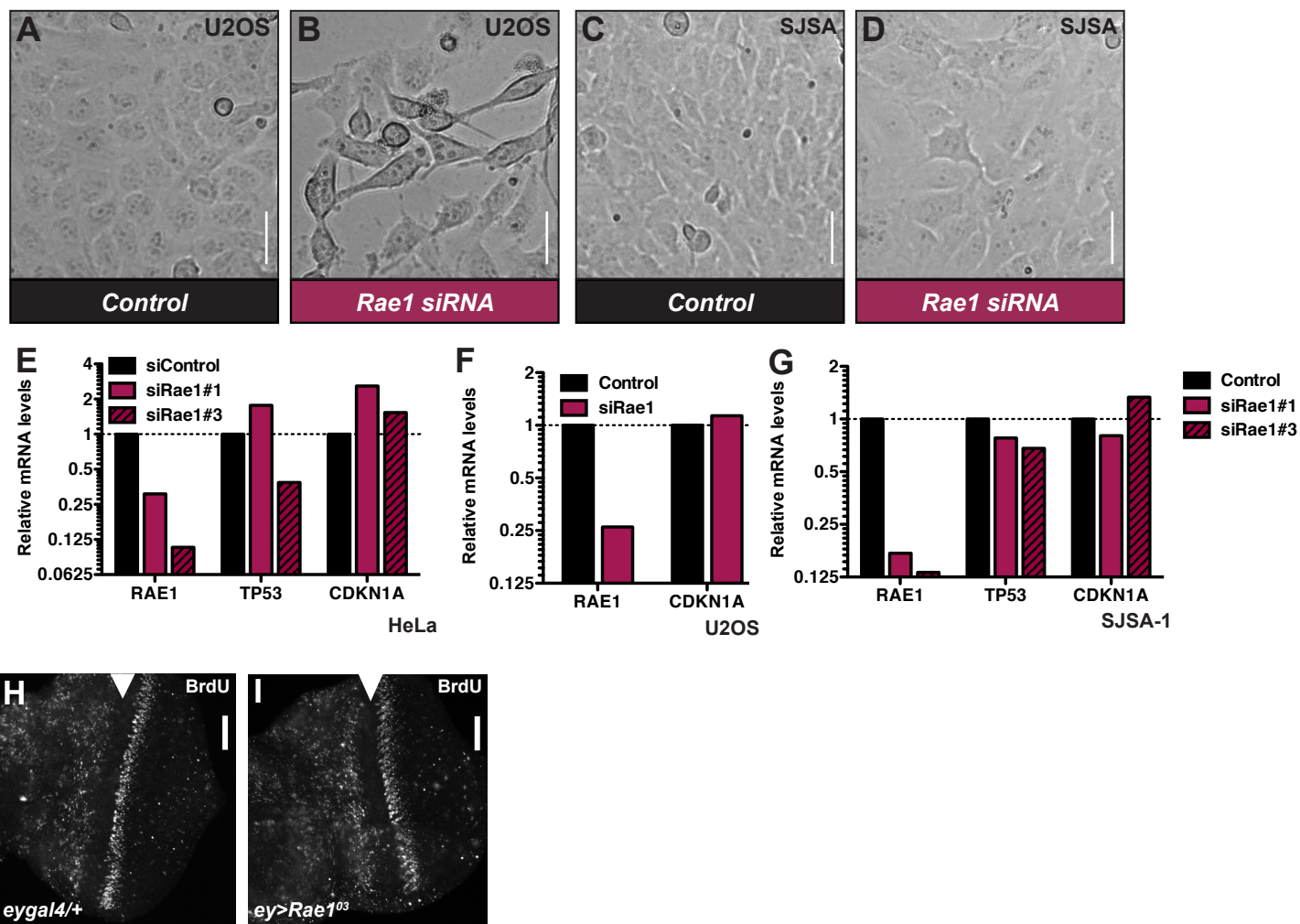

Supplement: S7 Fig — (A-B) Rae1 loss in the U2OS osteosarcoma cell line by siRNA transfection (B) reduces cell proliferation compared to controls (A). (C-D) Rae1 loss in the SJSA osteosarcoma cell line by siRNA transfection (D) reduces cell proliferation compared to controls (C). (E-G) Proliferative arrest induced by Rae1 knockdown in HeLa (E), U2OS (F) and SJSA (G) cells is not mediated by increased p21 (CDKN1A) (mRNA levels were normalized to GAPDH). (H) BrdU incorporation in a control eygal4/+ disc. (I) BrdU incorporation in a disc over-expressing Rae1 in actively dividing cells in the early eye, ey>Rae102. Staining anterior to the MF increased, and the width of the SMW increased. Scale bars in A-D indicate 75 μm, in H-I indicate 25 μm. (PDF) [file pgen.1006198.s007.pdf]

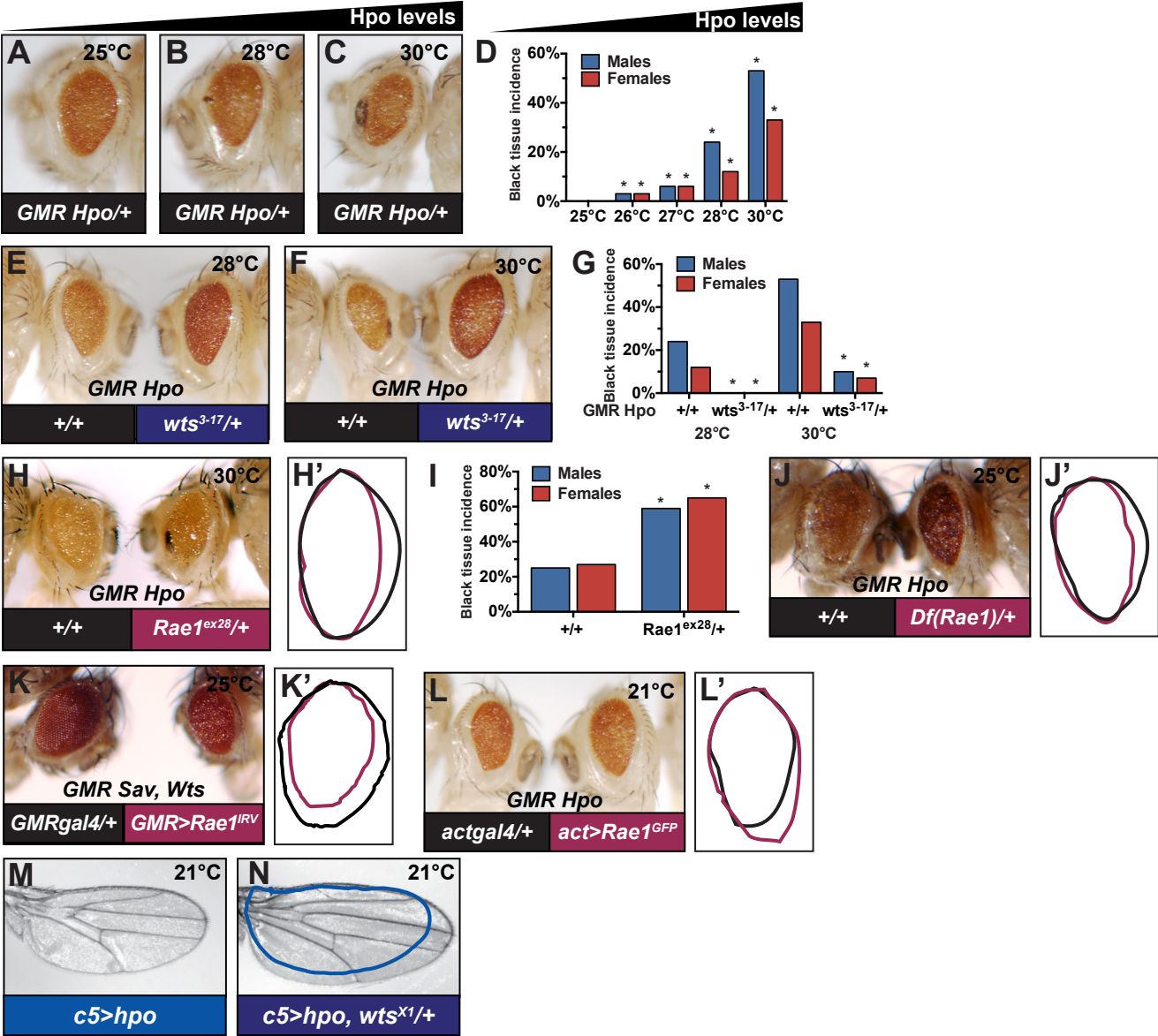

Supplement: S9 Fig — (A-C) Transgenic over-expression of hpo in differentiating eye cells (GMR Hpo) is responsive to temperature. Increasing the temperature increases expression levels of Hpo, and therefore increases the severity of the phenotype. GMR Hpo eyes were small and rough at 25°C (A), and became smaller, rougher, and showed increased appearance of black tissue at 28°C (B) and 30°C (C). (D) The presence of black tissue was quantified over a range of temperatures. N = 46, 60, 156, 38, 142 (males), N = 48, 58, 130, 60, 222 (females). (E-F) Removing one copy of wts by introducing the mutant allele wts3-17 suppressed the GMR Hpo eye size and black tissue phenotypes at 28°C (E) and 30°C (F). (G) Quantification of the black tissue showed modification of the GMR Hpo phenotype by adjusting wts gene dosage. This indicates that the black tissue phenotype can be used to reflect genetic modification of Hpo over-expression phenotypes. N = 38, 30, 142, 42 (males), N = 60, 34, 222, 44 (females). (H) Removing one copy of Rae1 by introducing the deletion allele Rae1ex28 (right eye, pink tracing in H’) further reduced GMR Hpo (left eye, black tracing in H’) eye size at 30°C (highlighted by tracings in H’) and enhanced the appearance of black tissue. (I) Quantification of black tissue appearance highlights the dominant enhancement by the Rae1ex28 allele at 28°C. (J-J’) Removing one copy of Rae1 using the deficiency Df(2R)ED3923 (right eye in J, pink tracing in J’), enhanced the phenotype of GMR Hpo (left eye in J, black tracing in J’) in terms of increasing eye roughness and further reducing eye size. (K-K’) Rae1 RNAi in differentiating eye cells using GMRgal4 and Rae1IRV (GMR>Rae1IRV) (right in in K, pink tracing in K’) enhanced the phenotype of over-expressing Sav and Wts in differentiating eye cells (GMR Sav, Wts) (left eye in K, black tracing in K’). (L-L’) Constitutively over-expressing Rae1 (act>Rae1GFP, right in L, pink tracing in L’) suppressed the phenotype of GMR Hpo (left in L, black tr [file pgen.1006198.s009.pdf]

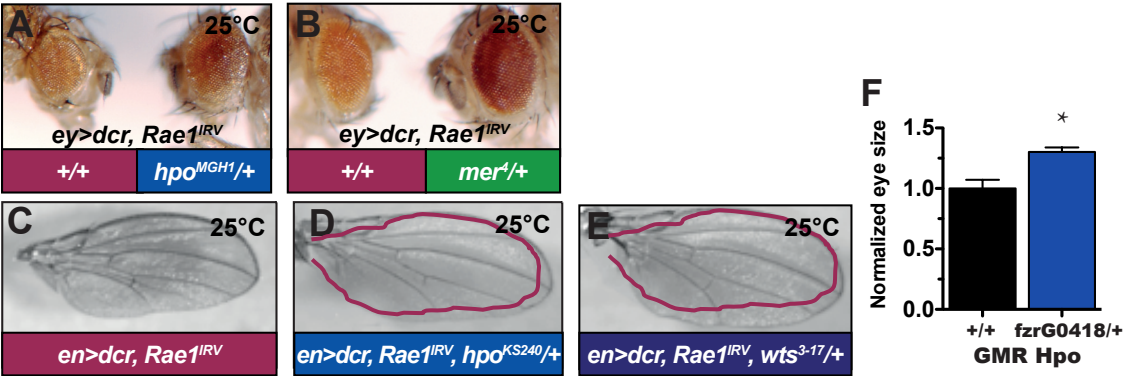

Supplement: S10 Fig — (A-B) Mutation in hpo (right eye in A) and Mer (right eye in B) dominantly suppressed the reduced eye size and eye roughness of RNAi to Rae1 in the early eye (left eyes in A, B). (C) Control en>dcr, Rae1IRV wing (and tracing in in D-E,). (D-E) Mutations in hpo (E) and wts (E) dominantly suppressed the reduced wing size of RNAi to Rae1 in the posterior wing, highlighted by overlay of tracing of the wing in (C) (pink). (F) Quantification of eye size indicating significant suppression of the reduced eye size of GMR Hpo by mutation in Cdh1/fzr (fzrG0418 allele shown) as seen in Fig 5H and 5H’. N = 29, 6. * indicates statistically significant difference p<0.05. (PDF) [file pgen.1006198.s010.pdf]

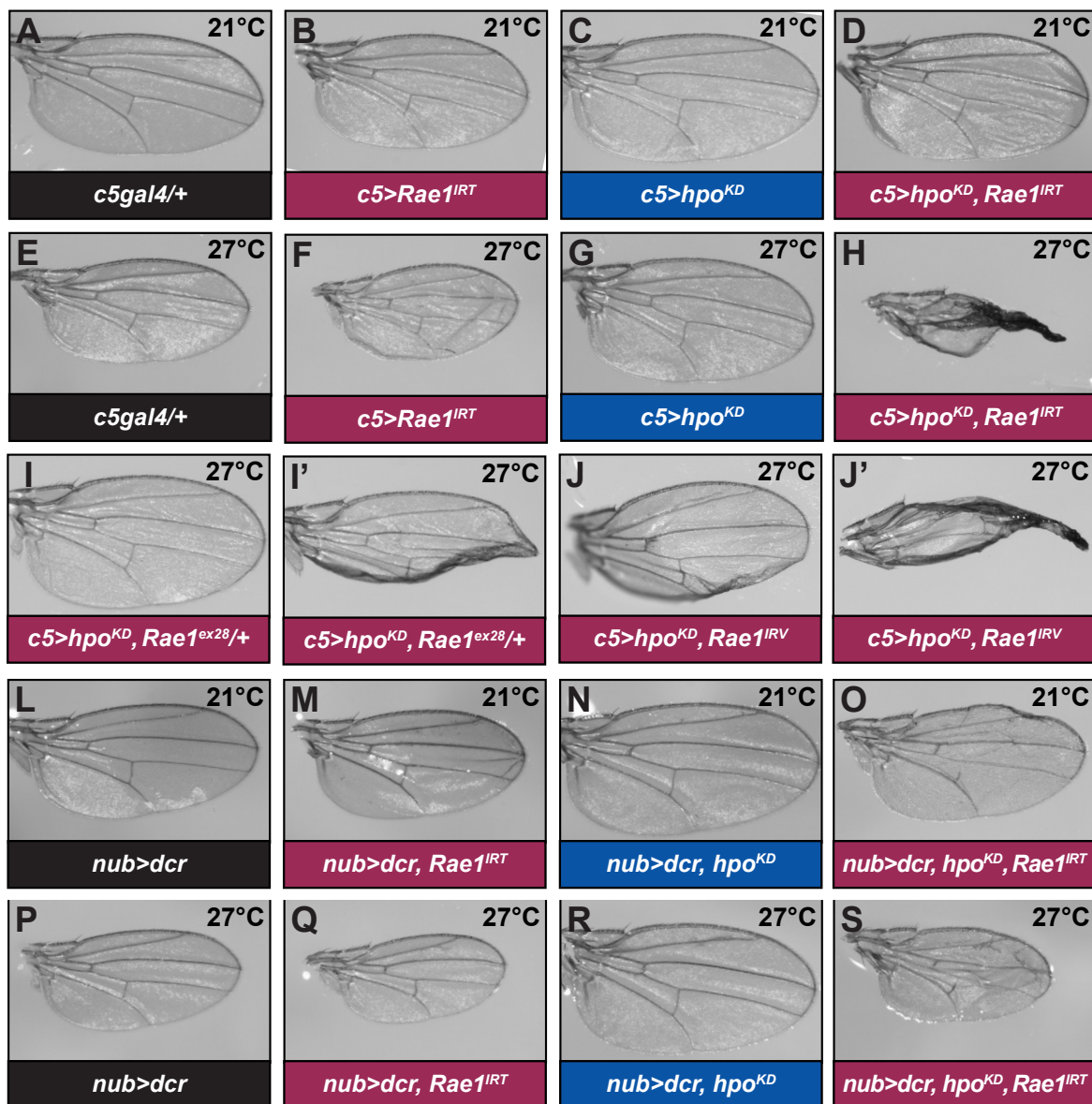

Supplement: S11 Fig — (A) Control wing (c5gal4/+). (B) RNAi to Rae1 (c5>Rae1IRT) causes a moderate reduction in wing size. (C) Over-expression of a kinase-dead Hpo transgene (c5>hpoKD) moderately increases wing size relative to controls (A). (D) This moderate overgrowth is suppressed by RNAi to Rae1. (c5>hpoKD, Rae1IRT). (E) Control wing (c5gal4/+). (F) RNAi to Rae1 (c5>Rae1IRT) causes a more dramatic reduction in wing size at 27°C. (G) Over-expression of a kinase-dead Hpo transgene (c5>hpoKD) promotes wing overgrowth relative to controls (E). (H) RNAi to Rae1 suppresses the dramatic overgrowth and also impairs the survival of overgrowing tissue caused by loss of Hippo activity (c5>hpoKD, Rae1IRT). (I-I’) Reducing Rae1 levels by introducing the Rae1ex28 allele caused a low penetrance of tissue collapse in the background of hippo signaling impairment (c5>hpoKD, Rae1ex28/+, I’). (J-J’) The Rae1IRV shows less dramatic phenotypes than the Rae1IRT transgene. Reducing Rae1 levels by RNAi caused a moderate penetrance of tissue collapse (c5>hpoKD, Rae1IRV, J’). (L) Control wing (nub>dcr). (M) RNAi to Rae1 (nub>dcr, Rae1IRT) causes a reduction in wing size. (N) Over-expression of a kinase-dead Hpo transgene (nub>dcr, hpoKD) increases wing size relative to controls (L). (O) This overgrowth is suppressed by RNAi to Rae1 (nub>dcr, hpoKD, Rae1IRT). (P) Control wing (nub>dcr) at 27°C. (Q) RNAi to Rae1 (nub>dcr, Rae1IRT) causes a more dramatic reduction in wing size at 27°C. (R) Over-expression of a kinase-dead Hpo (nub>dcr, hpoKD) increases wing size relative to controls at 27°C (R). (S) RNAi to Rae1 suppresses this overgrowth and promotes shriveling and blistering of wings (nub>dcr, hpoKD, Rae1IRT). (PDF) [file pgen.1006198.s011.pdf]

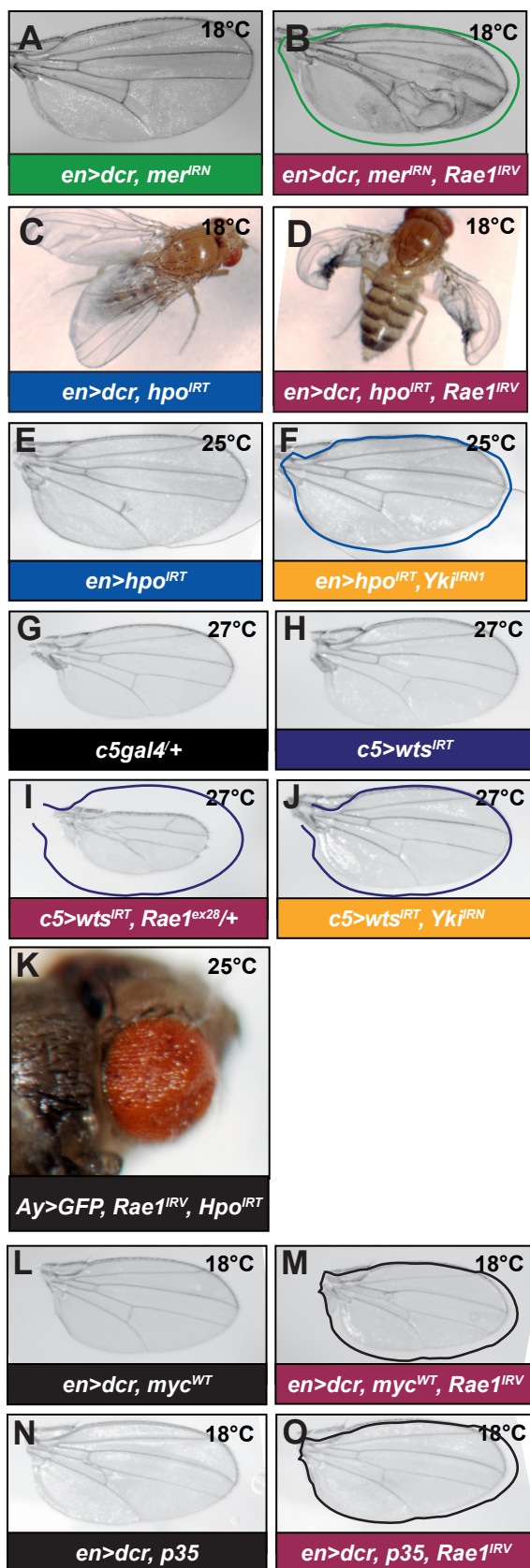

Supplement: S12 Fig — (A-B) Typically, RNAi to Mer (A, en>dcr, MerIRN) leads to less overgrowth than RNAi to the other tumor suppressor components ex, hpo, and wts. In cases where we saw less overgrowth, concurrently reducing Rae1 (B, en>dcr, MerIRN, Rae1IRV) suppressed overgrowth and often led to blistering and other mild phenotypes of tissue loss. This is consistent with the requirement for Rae1 changing depending on the extent of overgrowth. (C-D) Overgrowth due to loss of Hippo Pathway tumor components in the wing is often so dramatic that wings no longer lie flat; mounted wings thus wrongly appear smaller once they are flattened to be photographed. These images of flies with their wings still attached highlight how extensive the overgrowth is upon hpo RNAi (C, en>dcr, hpoIRT) and how effectively reducing Rae1 suppressed this overgrowth (D, en>dcr, hpoIRT, Rae1IRV). (E-F) Reducing yki levels did not cause the same tissue lethality we saw with Rae1; yki RNAi (F, en>hpoIRT, ykiIRN) suppressed the overgrowth but did not impair the survival of tissue overgrowing due to loss of hpo (E and blue tracing in F, en>hpoIRT). (G) Control c5gal4/+ wing. (H) RNAi to wts (c5>wtsIRT) caused wing overgrowth. (I) Removing one copy of Rae1 (c5>wtsIRT; Rae1ex28/+) caused tissue loss. (J) RNAi to yki (c5>wtsIRT; ykiIRN) suppressed overgrowth but did not cause tissue loss. (K) Creating random clones undergoing concurrent RNAi to both Rae1 and hpo causes patches of dying tissue throughout the fly. Image is of a dissected pharate adult showing black spots in the eye, and other large swaths of black tissue elsewhere. (L-O) The tissue lethality phenotype appears to be specific and did not occur for other overgrowth phenotypes we tested, shown here for over-expressing the myc oncogene and the caspase inhibitor p35. (L, black tracing in M) Control wing over-expressing the myc oncogene (en>dcr, mycWT). (M) Rae1 RNAi suppressed the overgrowth but did not cause tissue lethality in myc over-expressing wings (en>dcr [file pgen.1006198.s012.pdf]

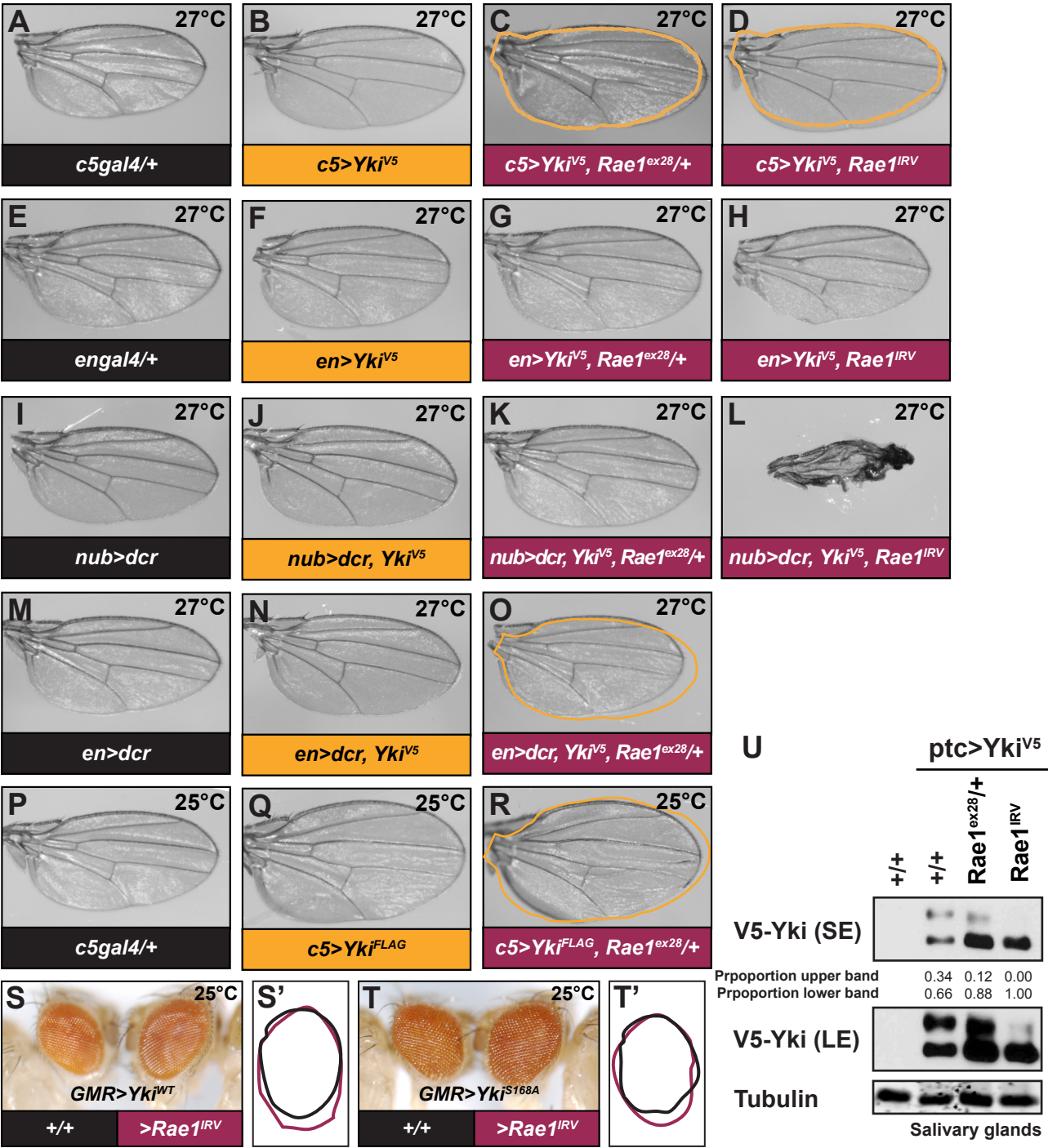

Supplement: S13 Fig — (A) Control c5gal4/+ wing at 27°C. (B) Yki over-expression (c5>YkiV5) caused wing overgrowth at 27°C. (C-D) Removing one copy of Rae1 by introducing Rae1ex28 (c5>YkiV5; Rae1ex28/+, C) or RNAi to Rae1 (c5>YkiV5; Rae1IRV/+, D) at 27°C subtly but reproducibly increased overgrowth. (E) Control engal4/+ wing at 27°C. (F) Yki over-expression (en>YkiV5) caused mild wing overgrowth in the posterior compartment at 27°C. (G) Removing one copy of Rae1 by introducing Rae1ex28 (en>YkiV5; Rae1ex28/+) increased wing overgrowth caused by Yki over-expression. (H) RNAi to Rae1 (en>YkiV5; Rae1IRV/+) at 27°C suppressed overgrowth. (I) Control nub>dcr wing at 27°C. (J) Yki over-expression (nub>dcr, YkiV5) caused mild wing overgrowth in wing at 27°C. (K) Removing one copy of Rae1 by introducing Rae1ex28 (nub>dcr, YkiV5; Rae1ex28/+) increased wing overgrowth caused by Yki over-expression. (L) RNAi to Rae1 (nub>dcr, YkiV5; Rae1IRV/+) at 27°C dramatically suppressed overgrowth. (M) Control wing (en>dcr). (N) Over-expressing Yki leads to wing overgrowth (N, and yellow overlay in O). (O) Reducing Rae1 levels slightly by removing one copy suppressed tissue overgrowth but did not cause tissue collapse. (P) Control wing (c5gal4/+). (Q) Over-expressing a different Yki transgene leads to wing overgrowth (E, and yellow overlay in F). (R) Reducing Rae1 levels slightly by removing one copy did not cause tissue collapse. Overgrowth in this context is quite dramatic such that wings no longer lie flat; mounted wings thus wrongly appear smaller once they are flattened to be photographed. (S-S’) Over-expressing Yki in the differentiating cells of the adult eye causes a larger eye (left in S, black tracing in S’). Reducing Rae1 levels by concurrent Rae1 RNAi (GMR>YkiV5, Rae1IRV, right in S, pink tracing in S’) further increases eye size. (T-T’) Over-expressing a constitutively active Yki transgene in the differentiating cells of the adult eye causes a larger eye (left in T, black tracing in T’). Reducing R [file pgen.1006198.s013.pdf]

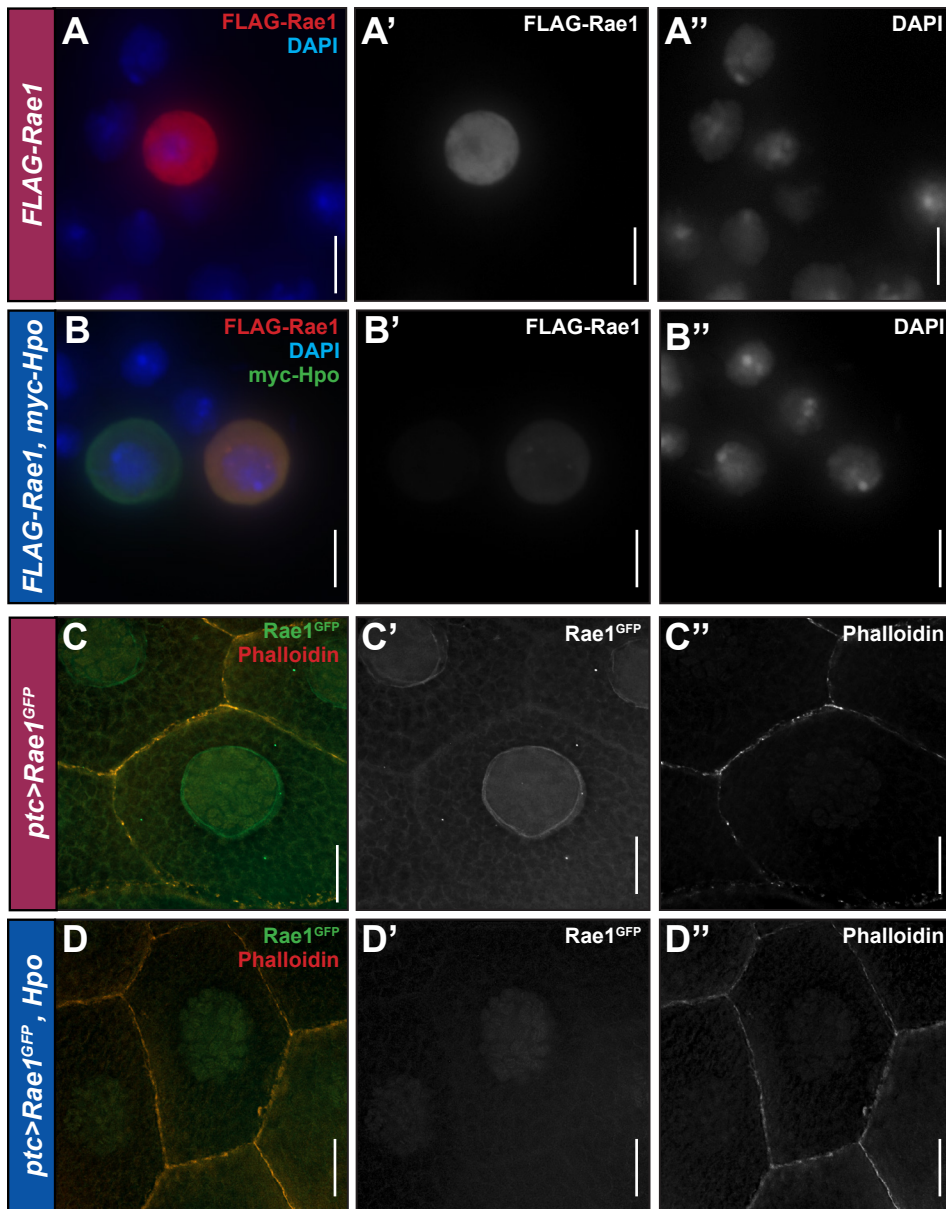

Supplement: S14 Fig — (A-A”) Transfecting FLAG-Rae1 into S2 cells shows Rae1 association with the cell periphery, cytoplasm, and nucleus. (B-B”) Co-transfecting FLAG-Rae1 with myc-Hpo into S2 cells reduced Rae1 levels, particularly at the cell periphery and cytoplasm. All images (A-B) were taken with identical exposure times and settings. Scale bars in A-B indicate 5 μm. (C-C”) Over-expressing a Rae1-GFP transgene in the salivary glands shows strong Rae1-GFP localization to the nuclear periphery but also localization to the cell membrane and cytoplasm. (D-D”) Co-over-expressing the Rae1-GFP with Hpo reduces the levels of Rae1 in salivary glands and specifically causes a reduction in the Rae1-GFP pools associated with the cell membrane and cytoplasm. There is also a reduction of Rae1 associated with the nuclear periphery and the pool of Rae1 in the nucleus appears to be associated with chromatin. All images (C-D) were taken with identical exposure times and settings. Scale bars in C-D indicate 20 μm. (PDF) [file pgen.1006198.s014.pdf]
