# Supplementary material for: The Hippo Pathway Targets Rae1 to Regulate Mitosis and Organ Size and to Feed Back to Regulate Upstream Components Merlin, Hippo, and Warts
Source: PLoS Genet. 2016 Aug 5;12(8):e1006198. doi: 10.1371/journal.pgen.1006198 (PMC4975479; doi:10.1371/journal.pgen.1006198)

Jahanshahi_Sup Fig 3

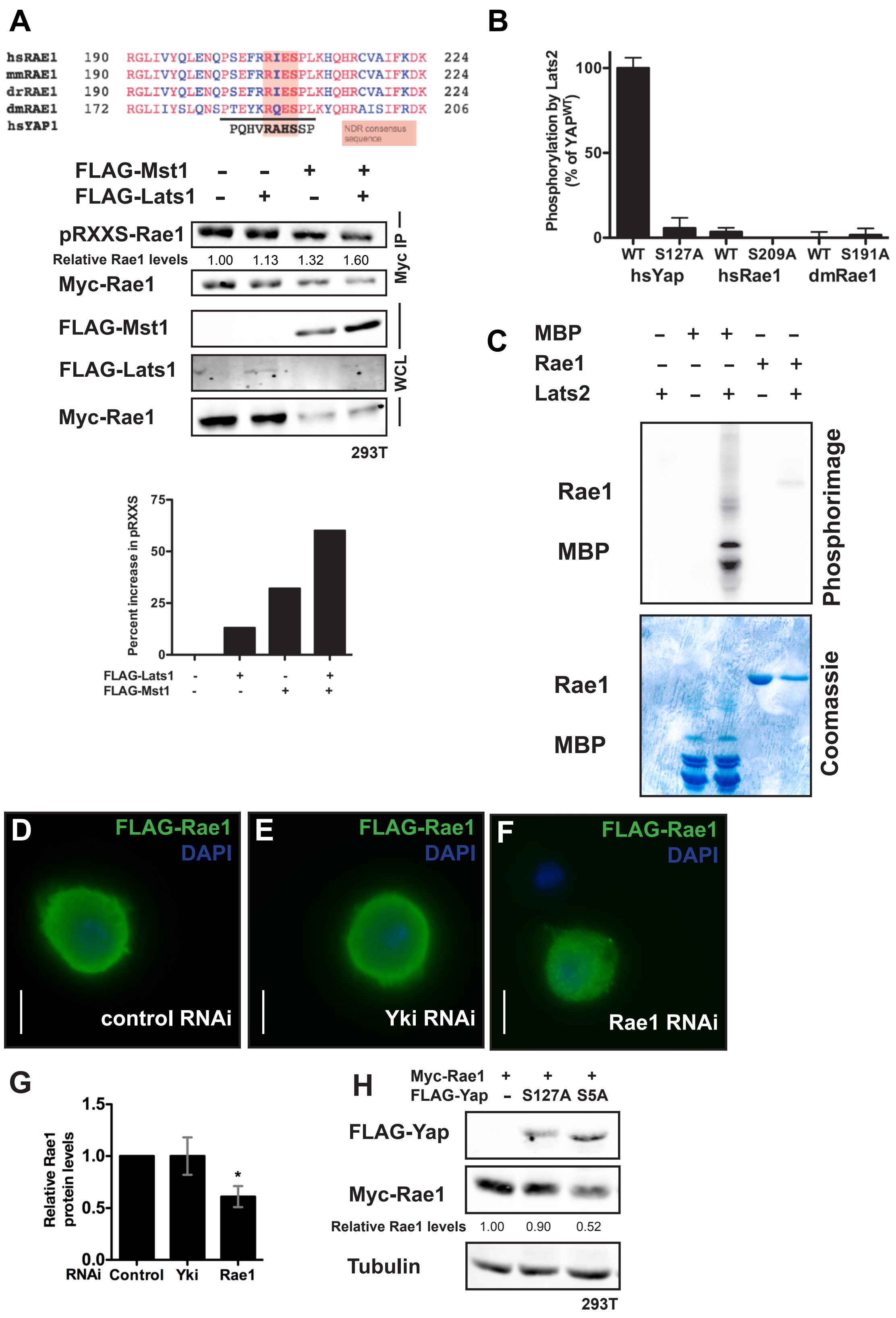

Supplement: S3 Fig — (A) The region surrounding the Lats1 consensus site (red box) in Rae1 is strongly conserved across species. Cells co-transfected with Mst1 and/or Lats1 and myc-Rae1 showed decreased Myc-Rae1 levels in the whole cell lysate (WCL) and also immunoprecipitated Rae1 (Myc-IP) as expected. Immunoprecipitated Rae1 was recognized by an anti-phospho-RXXS antibody (Lats1 consensus site), and the percentage of Rae1 phosphorylated at the Lats consensus motif increased with increased pathway activation. Relative levels of phosphorylated Rae1 are indicated. Quantification of anti-phospho-RXXS antibody (Lats1 consensus site) recognition of Myc-Rae1 immunoprecipitated from whole cell lysates of cells co-transfected with Mst1 and/or Lats1 are indicated as relative levels below the blot and in the graph below (normalized to the amount of immunoprecipitated total Rae1). (B) Peptides were generated using 11 amino acids (underlined in black in A) for Drosophila Rae1 (dmRae1) and human Rae1 (hsRae1) with alanine mutants that abolished the Wts consensus site (RXXA) Similar control and alanine mutant peptides were generated for YAP (hsYAP). A peptide kinase assay using Lats2 showed robust phosphorylation of wild-type YAP peptide but not of the S127A mutant peptide or of any of the Rae1 (wild-type or alanine mutant) peptides. (C) Kinase assays using Lats2, Myelin Basic Protein (MBP), and full length, purified baculovirus Rae1 [54] (a gift from Y. Ren and the Blobel lab). Coomassie gel shows levels of MBP and Rae1 protein used, and phosphorimage shows no significant phosphorylation of Rae1 in the presence of Lats2 compared to the MBP positive control. (D-F) Co-transfecting S2 cells with yki RNAi causes no change in Rae1 localization (E) compared to control-transfected cells (D). Rae1 RNAi causes a reduction of the membrane-bound pool of FLAG-Rae1 (F). Scale bars in D-F indicate 5 μm. (G) Rae1 protein levels over three independent experiments were quantified upon Yki or Rae1 RNAi. (H) Over-exp [file pgen.1006198.s003.pdf]
