# Supplementary material for: The Hippo Pathway Targets Rae1 to Regulate Mitosis and Organ Size and to Feed Back to Regulate Upstream Components Merlin, Hippo, and Warts
Source: PLoS Genet. 2016 Aug 5;12(8):e1006198. doi: 10.1371/journal.pgen.1006198 (PMC4975479; doi:10.1371/journal.pgen.1006198)
Supplement: S1 Table — (DOCX) [file pgen.1006198.s015.docx]

| Allele | *ey>dcr, Rae1^IRV^* |
| --- | --- |
| *mer^4^* | suppressed |
| *hpo^KS240^* | suppressed |
| *hpo^KC202^* | suppressed |
| *hpo^MGH1^* | suppressed |
| *wts^X1^* | suppressed |
| *wts^3-17^* | suppressed |

**Supplemental Table S1: Genetic interactions between Hippo Pathway components and Rae1 in the eye**.
