# Supplementary material for: The Hippo Pathway Targets Rae1 to Regulate Mitosis and Organ Size and to Feed Back to Regulate Upstream Components Merlin, Hippo, and Warts
Source: PLoS Genet. 2016 Aug 5;12(8):e1006198. doi: 10.1371/journal.pgen.1006198 (PMC4975479; doi:10.1371/journal.pgen.1006198)
Supplement: S2 Table — Table summarizes key similarities and differences of over-expressing or reducing Rae1 and Yorkie in a variety of contexts, highlighting the different mechanisms of feedback regulation exerted upon the Hippo pathway. (DOCX) [file pgen.1006198.s016.docx]

|  | **Rae1^WT^** | **Yki^WT^** | **Yki^S168A^** | **Rae1^RNAi^** | **Yki^RNAi^** |  |
| --- | --- | --- | --- | --- | --- | --- |
| **Early eye**  (eygal4) | Increase in size (10%),  normal patterning | Small/no eye | Small/no eye  Semi-lethal | Decrease in size (20%),  normal patterning | No obvious effect |  |
| **Differentiating eye cells**  (GMRgal4) | No obvious effect | Massive overgrowth | Massive overgrowth | No obvious effect | No obvious effect |  |
| **Posterior compartment**  (engal4) | Increase in size (10%),  normal patterning | Increase in size (10%),  normal patterning | Lethal | Decrease in size (20%),  normal patterning | Decrease in size (10%),  normal patterning |  |
| **Wing**  (c765gal4) | Increase in size (10%), normal patterning | Increase in size (20%), normal patterning | Lethal | Decrease in size (20%), normal patterning. | Decrease in size (10%),  normal patterning |  |
| **Constitutive**  (actgal4, tubgal4) | Increase in size (10%),  normal patterning | Increase in size (20%),  small/no eye | Lethal | Decrease in size (20%, lethal. | No obvious effect |  |
| **Salivary glands**  (ptcgal4, dppgal4) | No obvious effect | No obvious effect | Dramatic decrease  in size, lethal | No obvious effect | No obvious effect |  |
| **Cell cycle phenotype** | **Increased proliferation in actively dividing cells** | **Cell cycle re-entry, ectopic proliferation** | **Cell cycle re-entry, ectopic proliferation** | **Decreased proliferation, mitotic exit phenotypes** | **Decreased proliferation** |  |
| **Feedback circuit** | Post-translational | Transcriptional | Transcriptional | **✓** | **✓** | **Hippo knockdown overgrowth suppression** |
|  |  |  |  | **✓** | ✘ | **Tissue synthetic lethality** |

**Supplemental Table S2: Summary of comparisons and differences between roles and phenotypes of Rae1 and Yorkie.**
