# Supplementary material for: The Hippo Pathway Targets Rae1 to Regulate Mitosis and Organ Size and to Feed Back to Regulate Upstream Components Merlin, Hippo, and Warts
Source: PLoS Genet. 2016 Aug 5;12(8):e1006198. doi: 10.1371/journal.pgen.1006198 (PMC4975479; doi:10.1371/journal.pgen.1006198)
Supplement: S8 Fig — (A) wtsX1 MARCM eye discs (lane 2) show increased cyclin A and E protein levels compared to control MARCM discs (lane 1) consistent with previous reports. (B) wtsX1 MARCM eye discs (lane 2) show increased cycB protein levels compared to control MARCM discs (lane 1). Relative levels of cyclins (normalized to Tubulin) are indicated in A and B. (C-C’) Clones undergoing Rae1 RNAi (green in C, white tracing in C’) showed reduced cycA staining (red) in all regions of the eye disc with actively proliferating cells. Quantifying staining intensity suggests a decrease of more than 35% in the Rae1 RNAi clone in the SMW. (D-F) Mutation in cycA (E) or cycB (F) enhances the eye size reduction of RNAi to Rae1 in the early eye (ey>dcr, Rae1IRV, D). (G) Quantification of eyes in D-F. N = 16, 13, 12 (males), N = 17, 14, 16 (females). (H) Mutation in cycA or in cycB does not dominantly reduce wing size. N = 13, 14, 12 (males), N = 12, 13, 16 (females). (I-L) Over-expressing cycE (J), cycA (K), or cycB3 (L) partially suppresses the eye size reduction of RNAi to Rae1 in the early eye (ey>dcr, Rae1IRV, I). (M) Quantification of eyes from I-L except male eyes over-expressing cycA. Increased lethality of cycA over-expression in this context resulted in few males for quantification. N = 14, 13, 13 (males), N = 12, 12,1 8, 15 (females). (N-Q) Increased expression of cycE (O), cycA (P), or cycB3 (Q) in the early eye does not increase eye size compared to controls (N). (R) Quantification of eyes in N-Q. Increased lethality of cycA over-expression in this context resulted in few males for quantification. CycA over-expression on its own reduced eye size. N = 12, 17, 12 (males), N = 16, 17, 13, 17 (females). (S-S’) Rae1 over-expressing clones (green in S, tracing in S’) show increased cycA staining (red). Arrow in D’ indicates more intense cycA staining. Quantifying staining intensity suggests cycA staining increases more than 50% in the SMW. Arrowheads in S-S’ indicate the MF. (T-U) Over-express [file pgen.1006198.s008.pdf]

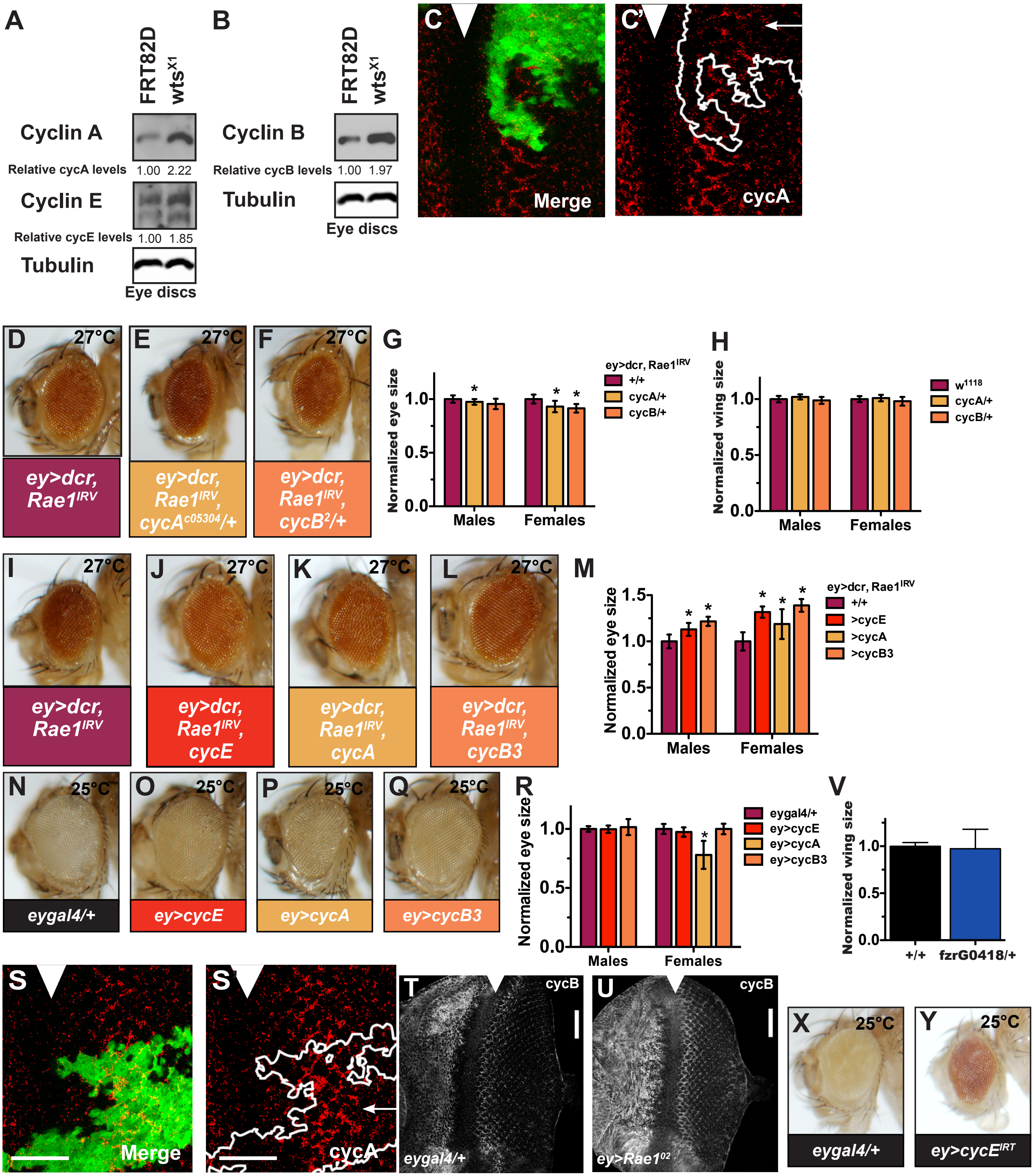
A
FRT82D
wtsX1
Cyclin A
Relative cycA levels 1.00 2.22
Cyclin E
Relative cycE levels 1.00 1.85
Tubulin
Eye discs
B
FRT82D
wtsX1
Cyclin B
Relative cycB levels 1.00 1.97
Tubulin
Eye discs
C
Merge
C'
cycA
D 27°C
ey>dcr, Rae1IRV
E 27°C
ey>dcr, Rae1IRV, cycAc05304/+
F 27°C
ey>dcr, Rae1IRV, cycB2/+
G
Normalized eye size
ey>dcr, Rae1IRV
+/+
cycA/+
cycB/+
Males
Females
H
Normalized wing size
w1118
cycA/+
cycB/+
Males
Females
I 27°C
ey>dcr, Rae1IRV
J 27°C
ey>dcr, Rae1IRV, cycE
K 27°C
ey>dcr, Rae1IRV, cycA
L 27°C
ey>dcr, Rae1IRV, cycB3
M
Normalized eye size
ey>dcr, Rae1IRV
+/+
>cycE
>cycA
>cycB3
Males
Females
N 25°C
eygal4/+
O 25°C
ey>cycE
P 25°C
ey>cycA
Q 25°C
ey>cycB3
R
Normalized eye size
eygal4/+
ey>cycE
ey>cycA
ey>cycB3
Males
Females
V
Normalized wing size
+/+
fzrG0418/+
S
Merge
S'
cycA
T
cycB
eygal4/+
U
cycB
ey>Rae102
X 25°C
eygal4/+
Y 25°C
ey>cycEIRT
